# Targeting loop3 of sclerostin preserves its cardiovascular protective action and promotes bone formation

**DOI:** 10.1038/s41467-022-31997-8

**Published:** 2022-07-22

**Authors:** Yuanyuan Yu, Luyao Wang, Shuaijian Ni, Dijie Li, Jin Liu, Hang Yin Chu, Ning Zhang, Meiheng Sun, Nanxi Li, Qing Ren, Zhenjian Zhuo, Chuanxin Zhong, Duoli Xie, Yongshu Li, Zong-Kang Zhang, Huarui Zhang, Mei Li, Zhenlin Zhang, Lin Chen, Xiaohua Pan, Weibo Xia, Shu Zhang, Aiping Lu, Bao-Ting Zhang, Ge Zhang

**Affiliations:** 1 Law Sau Fai Institute for Advancing Translational Medicine in Bone and Joint Diseases (TMBJ), School of Chinese Medicine, Hong Kong Baptist University, Hong Kong SAR, China; 2 Guangdong-Hong Kong-Macao Greater Bay Area International Research Platform for Aptamer-based Translational Medicine and Drug Discovery (HKAP), Hong Kong SAR, China; 3 Institute of Precision Medicine and Innovative Drug Discovery (PMID), School of Chinese Medicine, Hong Kong Baptist University, Hong Kong SAR, China; 4 Institute of Integrated Bioinformedicine and Translational Science (IBTS), School of Chinese Medicine, Hong Kong Baptist University, Hong Kong SAR, China; 5 School of Chinese Medicine, Faculty of Medicine, The Chinese University of Hong Kong, Hong Kong SAR, China; 6 Department of Materials Science and Engineering, Southern University of Science and Technology, Shenzhen, China; 7 Department of Endocrinology, National Health Commission Key Laboratory of Endocrinology, Peking Union Medical College Hospital, Chinese Academy of Medical Sciences and Peking Union Medical College, Beijing, China; 8 Shanghai Clinical Research Center of Bone Disease, Department of Osteoporosis and Bone Disease, Shanghai Jiao Tong University Affiliated Sixth People’s Hospital, Shanghai, China; 9 Department of Wound Repair and Rehabilitation Medicine, State Key Laboratory of Trauma, Burns and Combined Injury, Trauma Center, Research Institute of Surgery, Daping Hospital, Army Medical University, Chongqing, China; 10 Orthopedic Center, The Second Affiliated Hospital of Shenzhen University (People’s Hospital of Shenzhen Baoan District), Shenzhen, China; 11 The Key Laboratory of Aerospace Medicine, Ministry of Education, Air Force Medical University, Xi’an, Shaanxi China

**Keywords:** Bone, Cardiovascular biology, Target identification, Translational research

## Abstract

Sclerostin negatively regulates bone formation by antagonizing Wnt signalling. An antibody targeting sclerostin for the treatment of postmenopausal osteoporosis was approved by the U.S. Food and Drug Administration, with a boxed warning for cardiovascular risk. Here we demonstrate that sclerostin participates in protecting cardiovascular system and inhibiting bone formation via different loops. Loop3 deficiency by genetic truncation could maintain sclerostin’s protective effect on the cardiovascular system while attenuating its inhibitory effect on bone formation. We identify an aptamer, named aptscl56, which specifically targets sclerostin loop3 and use a modified aptscl56 version, called Apc001PE, as specific in vivo pharmacologic tool to validate the above effect of loop3. Apc001PE has no effect on aortic aneurysm and atherosclerotic development in *ApoE*^*−/−*^ mice and *hSOST*^*ki*^*.ApoE*^*−/−*^ mice with angiotensin II infusion. Apc001PE can promote bone formation in *hSOST*^*ki*^ mice and ovariectomy-induced osteoporotic rats. In summary, sclerostin loop3 cannot participate in protecting the cardiovascular system, but participates in inhibiting bone formation.

## Abstract (web summary)

Antibodies targeting sclerostin can ameliorate postmenopausal osteoporosis but present some cardiovascular risk. Here the authors show that the cardiovascular and skeletal effects of sclerostin are mediated by different loops, suggesting ways to preserve the positive effects on bone formation while avoiding the negative cardiovascular consequences.

## Introduction

Sclerostin can bind to low-density lipoprotein receptor-related protein 5/6 (LRP5/6) and inhibit bone anabolic Wnt signalling^1^. Loss of function mutations in the sclerostin-encoding gene *SOST* resulted in increased bone mass and bone strength^2–4^. Therefore, sclerostin is a target for bone anabolic therapy to reverse established osteoporosis^5^. Humanized antibody against sclerostin for postmenopausal osteoporosis therapy was approved by the U.S. Food and Drug Administration (FDA) in April 2019, with a boxed warning on its labelling stating that this treatment may increase the risk of heart attack, stroke and cardiovascular death and should not be used in patients who have had a heart attack or stroke within one year. Therefore, it is desirable to develop next-generation sclerostin inhibitors without cardiovascular risks. Meta-analysis of 25 cardiac ischaemic events in 4298 individuals from phase III clinical trials showed that therapeutic antibody (210 mg monthly) led to a higher risk of diseases. Estimates from a meta-analysis of ‘serious cardiovascular events’ were directionally concordant with increased vascular risk arising from therapeutic antibody treatment^6^. To date, the mechanism for the cardiovascular risk of the therapeutic antibody remains unclear.

**Fig. 1:**
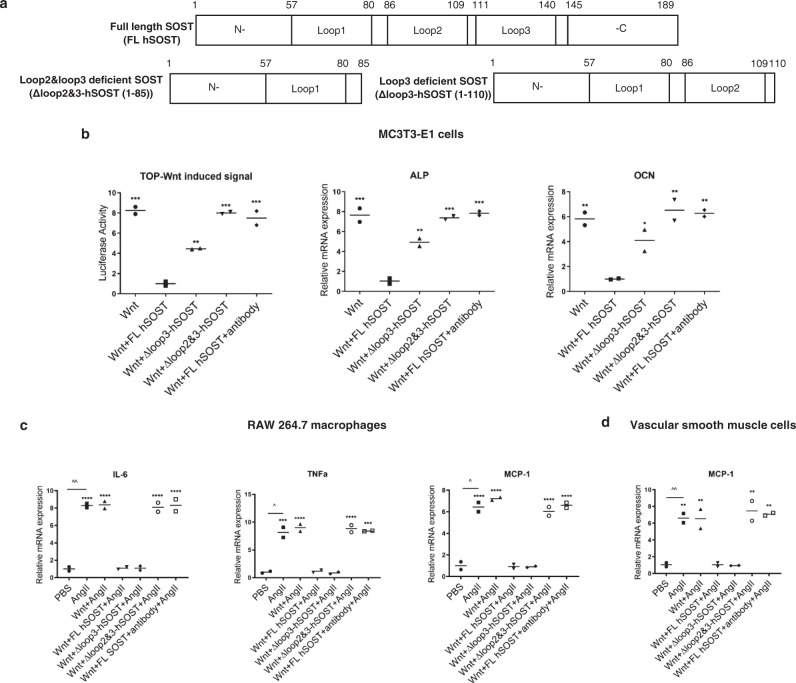
Effects of sclerostin loops on regulating Wnt signalling and osteogenic potential in MC3T3-E1 cells, and on regulating inflammatory cytokines/chemokines in macrophages and vascular smooth muscle cells (VSMCs). **a** Schematic diagram of the primary structures of full-length sclerostin and sclerostin truncations. Full-length human sclerostin (FL hSOST), sclerostin with loop2&3 deficiency by genetic truncation (Δloop2&3-hSOST(1-85)) and sclerostin with loop3 deficiency by genetic truncation (Δloop3-hSOST(1-110)). **b** The effect of sclerostin on regulating Wnt signalling and osteogenic potential in MC3T3-E1 cells. *p < 0.05; **p < 0.01 and ***p < 0.005 for a comparison *vs*. Wnt+FL hSOST group by one-way ANOVA with Tukey’s post-hoc test. TOP-Wnt induced signal: *p* = 0.0001 (Wnt), *p* = 0.0034 (Wnt + Δloo*p*3-hSOST), *p* = 0.0001 (Wnt + Δloop2&3-hSOST), *p* = 0.0002 (Wnt + FL hSOST+antibody); ALP: *p* = 0.0002 (Wnt), *p* = 0.0026 (Wnt + Δloo*p*3-hSOST), *p* = 0.0003 (Wnt + Δloop2&3-hSOST), *p* = 0.0002 (Wnt + FL hSOST+antibody); OCN: *p* = 0.0066 (Wnt), *p* = 0.0404 (Wnt + Δloo*p*3-hSOST), *p* = 0.0037 (Wnt + Δloop2&3-hSOST), *p* = 0.0045 (Wnt + FL hSOST+antibody). **c** The effect of sclerostin on regulating the mRNA levels of cytokines/chemokines in RAW264.7 macrophages. ^*p* < 0.05 and ^^*p* < 0.01 for a comparison between the PBS and AngII groups by a two-tailed unpaired t test. *p* = 0.0022 (IL-6), *p* = 0.0164 (TNF-α), *p* = 0.0103 (MCP-1). ***p < 0.005 and ****p < 0.0001 for a comparison *vs*. the FL hSOST+AngII group by one-way ANOVA with Tukey’s post-hoc test. IL-6*: p* < 0.0001 (AngII, Wnt+AngII, Wnt + Δloop2&3-hSOST+AngII, Wnt+FL hSOST+antibody+AngII); TNF-α*: p* = 0.0001 (AngII, Wnt+AngII, Wnt + Δloop2&3-hSOST+AngII), *p* = 0.0001 (Wnt + FL hSOST+antibody+AngII); MCP-1*: p* < 0.0001 (AngII, Wnt+AngII, Wnt + Δloop2&3-hSOST+AngII, Wnt+FL hSOST+antibody+AngII). **d** The effect of sclerostin on regulating the mRNA level of chemokines in VSMCs. ^^*P* < 0.01 for a comparison between the PBS and AngII groups by an unpaired *t* test (two-tailed). *p* = 0.0116. **p < 0.01 for a comparison of with FL hSOST +AngII group by one-way ANOVA with Tukey’s post-hoc test. *p* = 0.0028 (AngII), *p* = 0.0030 *(*Wnt + AngII), *p* = 0.0012 (Wnt + Δloop2&3-hSOST+AngII), *p* = 0.0017 (Wnt + FL hSOST+antibody+AngII). For (**b**, **c** and **d**), *n* = 2 per group. All data were expressed as the mean ± standard deviation. Note: ALP: alkaline phosphatase*;* OCN: osteocalcin; AngII: angiotensin II; IL-6: interleukin 6; MCP-1: monocyte chemoattractant protein-1; TNF-α: tumour necrosis factor alpha. Source data are provided as a Source Data file.

Sclerostin demonstrated a protective role in the cardiovascular system and could inhibit inflammatory cytokines and chemokines to prevent both aortic aneurysm (AA) and atherosclerotic development induced by angiotensin II (AngII) in Apolipoprotein E deficient (*ApoE*^*−/−*^) mice with transgenic introduction of sclerostin or administration of recombinant sclerostin^7^. Therefore, the potential cardiovascular risk should be carefully examined to inhibit sclerostin in bone anabolic therapy. Sclerostin has long N- and C-terminal regions that are highly flexible and completely disordered. The remaining central residues form three loops, loop1, loop2 and loop3 (Fig. 1a)^8^. It was reported that therapeutic sclerostin antibody, which showed cardiovascular risks, could target both sclerostin loop2 and loop3^8^. It is desirable to understand the roles of different sclerostin loops in regulating inflammatory cytokines for cardiovascular diseases and bone formation for osteoporosis and to strategically identify the functional loops in sclerostin, which cannot participate in protecting the cardiovascular system but is involved in inhibiting bone formation.

In this work, we investigated the roles of sclerostin loops using both genetic and pharmacologic approaches in vitro. For osteogenic potential, we found that loop2 and/or loop3 played important roles in the inhibitory effect of sclerostin on Wnt signalling and osteogenic potential in vitro. For cardiovascular system, we found that either loop2&3 deficiency by genetic truncation or loop 2&3 inhibition by sclerostin antibody could attenuate the suppressive effects of sclerostin on the expression of inflammatory cytokines and chemokines, whereas loop3 deficiency by genetic truncation maintained the above suppressive effects of sclerostin in vitro. Our in vivo studies further demonstrated that loop 3 deficiency by genetic truncation could maintain the protective effect of sclerostin on the cardiovascular system but attenuate the inhibitory effect of sclerostin on bone formation. Furthermore, we confirmed that specifically targeting sclerostin loop3 by the pharmacologic approach could promote bone formation and maintain the sclerostin loop3-independent cardiovascular protective effect. With a combination of genetic approaches and pharmacologic approaches, we validated that, as a candidate functional loop in sclerostin, loop3 could participate in inhibiting bone formation but not in protecting the cardiovascular system.

## Results

### The role of sclerostin loop2 and loop3 in Wnt signalling and osteogenic potential in vitro

Osteogenic MC3T3-E1 cells were transfected with plasmids bearing Wnt and either full-length human sclerostin (FL hSOST) or sclerostin truncations (Supplementary Fig. 1). Compared to that in the cells transfected with FL hSOST, Wnt signalling and osteogenic potential, including alkaline phosphate (*ALP*) and osteocalcin (*OCN*), were significantly higher in the cells transfected with loop2 and loop3 deficient sclerostin (Δloop2&3-hSOST(1-85)) and loop3-deficient sclerostin (Δloop3-hSOST(1-110)), respectively (Fig. 1b). Furthermore, in the cells transfected with FL SOST, Wnt signalling and osteogenic potential were significantly higher after treatment with sclerostin antibody, which targeted both loop 2 and loop3 (Fig. 1b and Supplementary Fig. 2). The above data demonstrated that loop 2 and/or loop3 played important roles in sclerostin’s inhibitory effect on Wnt signalling and osteogenic potential in MC3T3-E1 cells.

### The role of sclerostin loop2 and loop3 in suppressing the expression of inflammatory cytokines and chemokines in vitro

RAW 264.7 macrophages were treated with AngII or vehicle for 24 h, followed by examination of the mRNA levels of inflammatory cytokines (*IL-6*, *TNFα*) and chemokines (*MCP-1*)^9–11^. All mRNA levels were significantly higher after AngII treatment than after vehicle treatment, indicating that AngII treatment could induce the elevation of inflammatory cytokines and chemokines in macrophages. RAW 264.7 macrophages were then treated with conditioned media from the MC3T3-E1 cells transfected with Wnt and either FL hSOST or SOST truncations and treated with AngII for 24 h. All mRNA levels were significantly lower after treatment with FL hSOST, indicating that FL hSOST could suppress the expression of inflammatory cytokines and chemokines. Furthermore, compared to those with FL hSOST, all mRNA levels were significantly higher with Δloop2&3-hSOST(1-85) but were not altered with Δloop3-hSOST(1-110) treatment. In the RAW 264.7 cells treated with FL hSOST, all mRNA levels were significantly higher after treatment with sclerostin antibody (Fig. 1c). Consistent results were shown in VSMCs (Fig. 1d). Thus, either loop2&3 deficiency by genetic truncation or loop2&3 inhibition by sclerostin antibody could attenuate the suppressive effects of sclerostin on the expression of inflammatory cytokines and chemokines, whereas loop3 deficiency by genetic truncation maintained the above suppressive effects of sclerostin in macrophages and VSMCs with AngII treatment in vitro.

### The effect of sclerostin loop3 deficiency by genetic truncation on the cardiovascular system in vivo

**Fig. 2:**
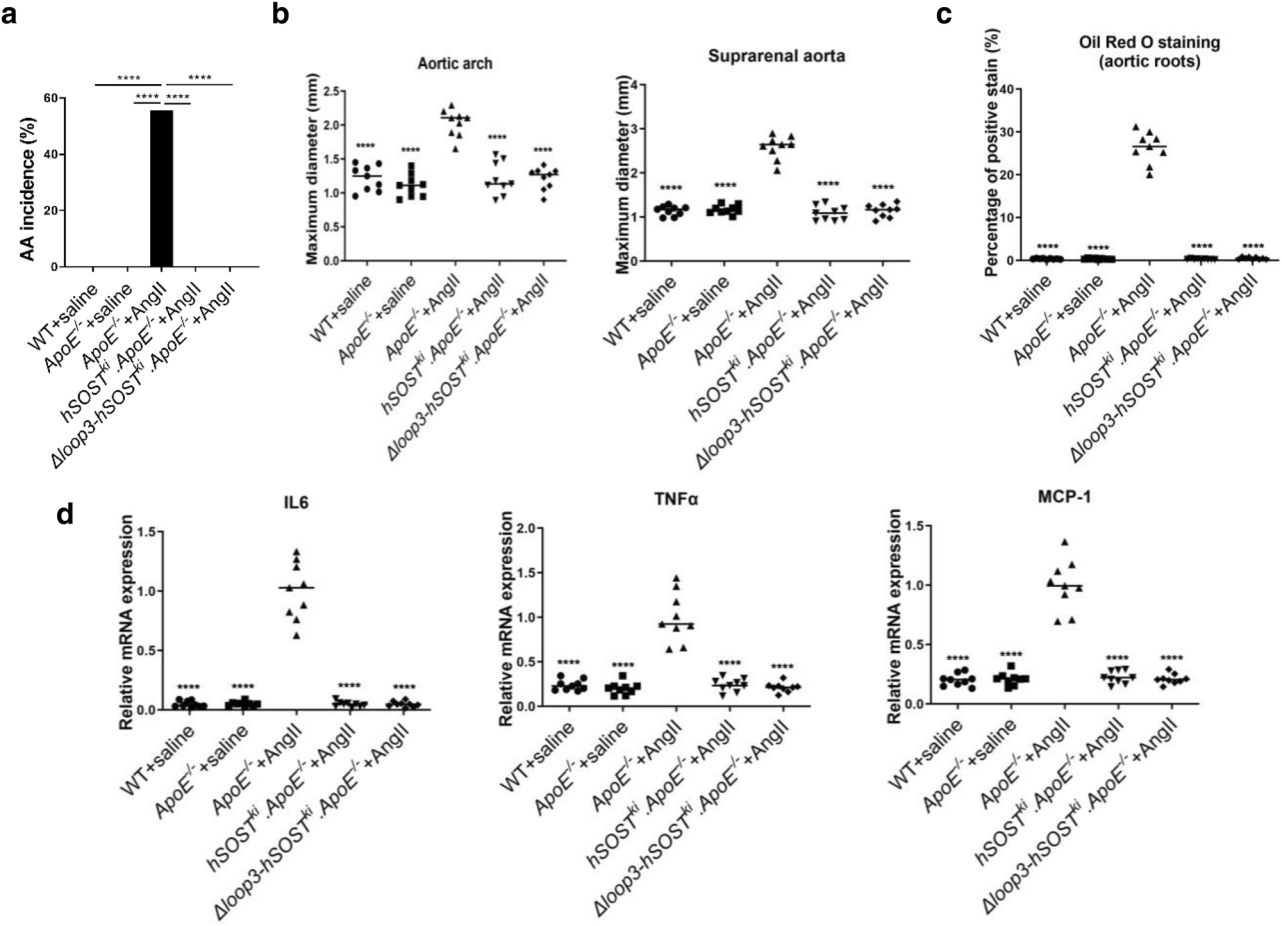
Determination of whether loop3-deficient sclerostin by genetic truncation could maintain the protective effect of sclerostin on the cardiovascular system in *ApoE*^*−/−*^ mice with loop3-deficient human sclerostin knock-in (*Δloop3-SOST*^*ki*^*.ApoE*^*−/−*^). Comparisons were performed among wide-type mice with saline infusion (WT + saline), *ApoE*^*−/−*^ mice with saline infusion (*ApoE*^*−/−*^+saline), *ApoE*^*−/−*^ mice with AngII infusion (*ApoE*^*−/−*^+AngII), *ApoE*^*−/−*^ mice with full-length human sclerostin knock-in and AngII infusion (*hSOST*^*ki*^*. ApoE*^*−/−*^*+*AngII) and *ApoE*^*−/−*^ mice with loop3-deficient human sclerostin (1-110) knock-in and AngII infusion (*Δloop3-SOST*^*ki*^*.ApoE*^*−/−*^+AngII). **a** The incidence of AA formation. A two-sided chi-square test was performed to determine the difference between two groups. ****p < 0.0001. *p* < 0.0001 for all comparisons. **b** Ex vivo measurement of the maximum diameters of the aortic arches (left) and suprarenal aortas (right). Aortic arch: *p* < 0.0001 for all grou*p*s; suprarenal aortas: *p* < 0.0001 for all groups. **c** Quantification of positive Oil Red O staining per cryosection indicating the ratio of atherosclerotic plaque area to total cross cryosection area (%) of the aortic roots. *p* < 0.0001 for all groups. **d** Serum levels of inflammatory cytokines (IL-6, TNF-α) and chemokines (MCP-1). IL-6: *p* < 0.0001 for all groups; TNF- α: *p* < 0.0001 for all grou*p*s; MCP-1: *p* < 0.0001 for all groups. For (**b** to **e**), data were expressed as the mean ± standard deviation. *n* = 9 per group. One-way ANOVA with Tukey’s post-hoc test vs. *ApoE*^*−/−*^+AngII was used to determine the intergroup differences. ****p < 0.0001. Note: AA: aortic aneurysm; AngII: angiotensin II; IL-6: interleukin 6; MCP-1: monocyte chemoattractant protein-1; TNF-α: tumour necrosis factor alpha. Source data are provided as a Source Data file.

Full-length sclerostin knock-in (*hSOST*^*ki*^) and loop3-deficient sclerostin knock-in *(Δloop3-hSOST*^*ki*^) mice were generated and crossed with *ApoE*^*−/−*^ mice to construct *hSOST*^*ki*^*. ApoE*^*−/−*^ and *Δloop3-SOST*^*ki*^*. ApoE*^*−/−*^ mice, respectively (Supplementary Fig. 3a–h). To investigate whether loop3 deficiency by genetic truncation could maintain the protective effect of sclerostin on the cardiovascular system in vivo, we determined parameters for AA and atherosclerotic development after saline or AngII infusion for four weeks. Compared to those in the *ApoE*^*−/−*^+AngII mice, the AA incidence for AA development (Fig. 2a and Supplementary Fig. 4a), the maximum ex vivo diameters of aortic arches and suprarenal aortas (Fig. 2b), and the atherosclerotic plaque area/total aortic root cross area ratios for atherosclerotic development (Fig. 2c & Supplementary Fig. 4b), as well as the serum levels of the inflammatory cytokines and chemokines, were significantly reduced in both the *hSOST*^*ki*^*.ApoE*^*−/−*^+AngII and *Δloop3-SOST*^*ki*^*.ApoE*^*−/−*^+AngII groups (Fig. 2d). There were no significant differences in the above parameters among the other groups. These data consistently demonstrated that loop3 deficiency by genetic truncation maintained the protective effect of sclerostin on the cardiovascular system in vivo.

### The effect of sclerostin loop3 deficiency by genetic truncation on bone formation in vivo

**Fig. 3:**
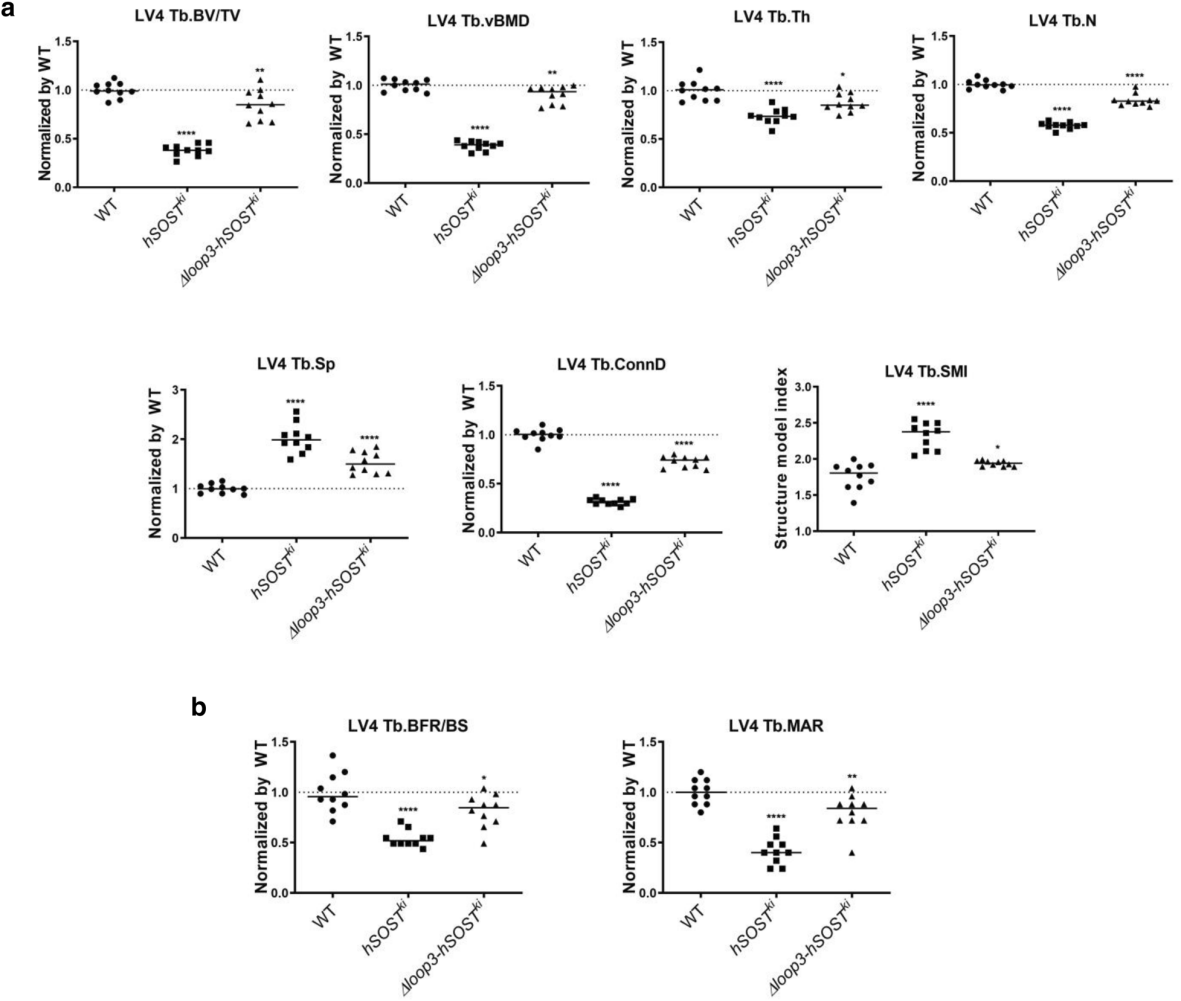
Determination of whether loop3 deficiency by genetic truncation could attenuate the inhibitory effect of sclerostin on bone formation in loop3-deficient sclerostin (*Δloop3-hSOST*^*ki*^*(1-110)*) knock-in mice compared to full-length sclerostin (*hSOST*^*ki*^) knock-in and WT mice. Mice were injected intraperitoneally with calcein green (20 mg/kg) at 10 and 2 days before euthanasia. After euthanasia, the fourth vertebrae of all mice were collected for analysis. **a** Bar charts of the structural parameters of Tb.BV/TV, Tb.vBMD, Tb.Th, Tb.N, Tb.Sp, Tb.conn.D and Tb.SMI from ex vivo micro-CT examination at the fourth vertebrae. Tb.BV/TV: *p* < 0.0001 (*hSOST*^*ki*^), *p* = 0.0076 (*Δloop3-hSOST*^*ki*^); Tb.vBMD: *p* < 0.0001 (*hSOST*^*ki*^), *p* = 0.0067 (*Δloop3-hSOST*^*ki*^); Tb.Th: *p* < 0.0001 (*hSOST*^*ki*^), *p* = 0.0134 (*Δloop3-hSOST*^*ki*^); Tb.N: *p* < 0.0001 (*hSOST*^*ki*^), *p* < 0.0001 (*Δloop3-hSOST*^*ki*^); Tb.Sp: *p* < 0.0001 (*hSOST*^*ki*^), *p* < 0.0001 (*Δloop3-hSOST*^*ki*^); Tb.ConnD: *p* < 0.0001 (*hSOST*^*ki*^), *p* < 0.0001 (*Δloop3-hSOST*^*ki*^); Tb.SMI: *p* < 0.0001 (*hSOST*^*ki*^), *p* = 0.0276 (*Δloop3-hSOST*^*ki*^). **b** Analysis of dynamic bone histomorphometric parameters of Tb.BFR/BS and Tb.MAR at the fourth vertebrae. Tb.BFR/BS: *p* < 0.0001 (*hSOST*^*ki*^), *p* = 0.0227 (*Δloop3-hSOST*^*ki*^); Tb.MAR: *p* < 0.0001 (*hSOST*^*ki*^), *p* = 0.0094 (*Δloop3-hSOST*^*ki*^). For (**a**) and (**b**), data were expressed as the mean ± standard deviation. *n* = 10 *p*er group. One-way ANOVA with Tukey’s post-hoc test vs. the WT group was used to determine the intergroup differences. *p < 0.05*;* **p < 0.01; ***p < 0.005; ****p < 0.0001. Note: micro-CT: microcomputed tomography; Tb.BV/TV: trabecular relative bone volume; Tb.vBMD: trabecular volumetric mineral density; Tb.Th: trabecular thickness; Tb.N: trabecular number; Tb.Sp: trabecular spacing; Tb.conn.D: trabecular connection density; Tb.SMI: trabecular structure model index; Tb.BFR/BS: trabecular bone formation rate; Tb.MAR: trabecular mineral apposition rate. Source data are provided as a Source Data file.

To investigate whether loop3 deficiency by genetic truncation could attenuate the inhibitory effect of sclerostin on bone formation in vivo, we evaluated bone mass, bone microstructure and bone formation in the WT, *hSOST*^*ki*^ and *Δloop3-hSOST*^*ki*^ mice. Microcomputed tomography (micro-CT) was used for the measurement of trabecular bone (below the growth plate) at the metaphysis of the fourth vertebrae (Fig. 3a and Supplementary Fig. 5a). Compared to those in the WT mice, the trabecular bone volume ratio (Tb.BV/TV), trabecular bone mineral density (Tb.vBMD), trabecular thickness (Tb.Th), trabecular number (Tb.N), and trabecular connectivity density (Tb.Conn.D) were significantly lower, but the trabecular spacing (Tb.Sp) was significantly higher in the *hSOST*^*ki*^ mice. The average absolute value of the trabecular structure model index (Tb.SMI) was close to 0 in the WT mice and close to 3 in the *hSOST*^*ki*^ mice, indicating the plate-like shape of the trabecular bone in the WT mice and the rod-like shape of the trabecular bone in the *hSOST*^*ki*^ mice at the fourth vertebrae. There were no significant differences between the *Δloop3-hSOST*^*ki*^ mice and the WT mice in any of the above parameters.

The bone histomorphometric analysis was used for measurement of the trabecular bone (below the growth plate) at the metaphysis of the fourth vertebrae. Compared to those in the WT mice, the trabecular bone formation rate (Tb.BFR/BS) and the trabecular bone mineral apposition rate (Tb.MAR) were notably lower in the *hSOST*^*ki*^ mice (Fig. 3b and Supplementary Fig. 5b). There were no significant differences between the *Δloop3-hSOST*^*ki*^ and WT groups in any of the above parameters. Both micro-CT and bone histomorphometric data consistently indicated that loop3 deficiency by genetic truncation could attenuate the inhibitory effect of sclerostin on bone formation in vivo.

### Selection and characterization of aptamer candidates against sclerostin loop3

The above in vivo data demonstrated that loop3 deficiency by genetic truncation could maintain the protective effect of sclerostin on the cardiovascular system while attenuating the inhibitory effect of sclerostin on bone formation. Therefore, whether specifically targeting sclerostin loop3 by pharmacologic approaches could promote bone formation and maintain sclerostin loop3-independent cardiovascular protective effects should be examined.

**Fig. 4:**
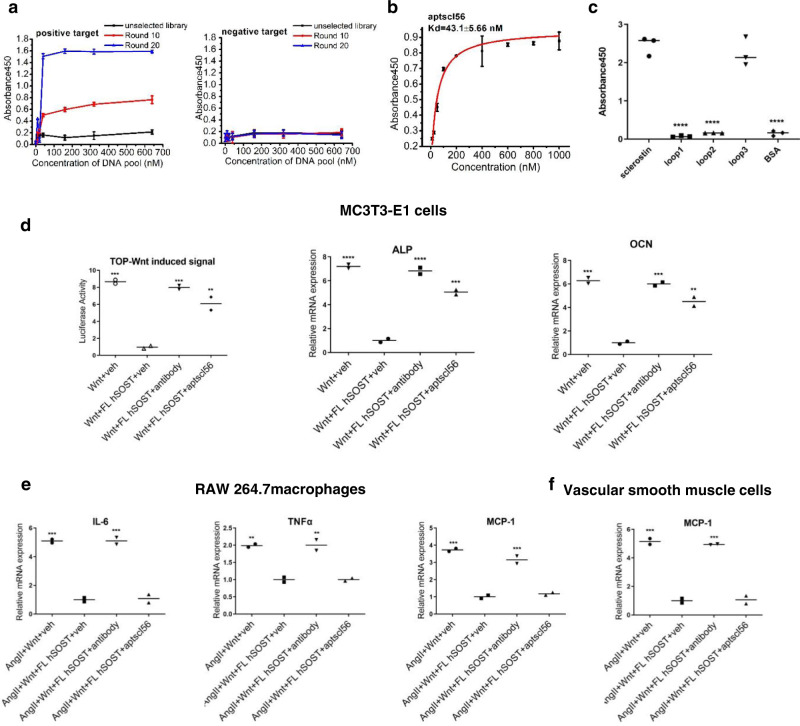
Selection and characterization of aptamer candidates against sclerostin loop3. **a** The binding ability of the enriched ssDNA pools and unselected library to full-length sclerostin (left) and loop3-deficient sclerostin (right). **b** The binding affinity of aptscl56 to full-length sclerostin. **c** The binding ability of aptscl56 to full-length sclerostin and sclerostin loops. One-way ANOVA with Tukey’s post-hoc test *vs*. FL hSOST was used to determine the intergroup differences. ****p < 0.0001*. p* < 0.0001 (loop1, loop2, BSA). For (**a** to **c**), data were expressed as the mean ± standard deviation. *n* = 3 per group. **d** The effect of aptscl56 on regulating Wnt signalling and osteogenic potential in MC3T3-E1 cells. One-way ANOVA with Tukey’s post-hoc test *vs*. Wnt+FL hSOST+veh (TOP-Wnt-induced signal) or vs. Wnt+FL hSOST+veh (ALP and OCN) was used to determine the intergroup differences. TOP-Wnt-induced signal: *p* = 0.0005 (Wnt + veh), *p* = 0.0007 (Wnt + FL SOST + antibody), *p* = 0.0024 (Wnt + FL SOST + aptscl56); ALP: *p* < 0.0001 (Wnt + veh, Wnt+FL SOST + antibody), *p* = 0.0003 (Wnt + FL SOST + aptscl56); OCN: *p* = 0.0003 (Wnt + veh), *p* = 0.0003 (Wnt + FL SOST + antibody), *p* = 0.0012 (Wnt + FL SOST + aptscl56). **e** The effect of aptscl56 on regulating mRNA levels of IL-6, TNFα and MCP-1 in RAW264.7 macrophages with AngII treatment. IL-6: *p* = 0.0004 (AngII + Wnt+veh), *p* = 0.0004 (AngII + Wnt+FL SOST + antibody); TNF-α: *p* = 0.0036 (AngII + Wnt+veh), *p* = 0.0034 (AngII + Wnt+FL SOST + antibody); MCP-1: *p* = 0.0003 (AngII + Wnt+veh), *p* = 0.0007 (AngII + Wnt+FL SOST + antibody). **f** The effect of aptscl56 on regulating the mRNA level of MCP-1 in VSMCs with AngII treatment. *p* = 0.0002 (AngII + Wnt+veh), *p* = 0.0003 (AngII + Wnt+FL SOST + antibody). For (**e**, **f**), one-way ANOVA with Tukey’s post**-**hoc test vs. FL hSOST+AngII+veh was used to determine the intergroup differences. For (**d**–**f**), data were expressed as the mean ± standard deviation. *n* = 2 per group. *p < 0.05; **p < 0.01; ***p < 0.005; ****p < 0.0001. Note: ALP: alkaline phosphatase; OCN: osteocalcin; AngII: angiotensin II; IL-6: interleukin 6; MCP-1: monocyte chemoattractant protein-1; TNF-α: tumour necrosis factor alpha. Source data are provided as a Source Data file.

Oligonucleotide aptamers are single-stranded DNA/RNAs that bind to their targets by 3D complementarity. Aptamers can be tailored selected against specific domains of the protein target by introducing positive and negative selections through a process called Systematic Evolution of Ligands by EXponential enrichment (SELEX)^12–14^. To identify aptamer candidates that target loop3 as a specific in vitro pharmacologic tool, we used full-length sclerostin as the positive target, and loop3-deficient sclerostin as the negative target for the identification of aptamers from a random ssDNA library composed of ~10^15^ different sequences. With increasing rounds of selection, the enrichment of high-affinity aptamers through SELEX was monitored (Fig. 4a). The representative candidate sequences from the final round were identified, synthesized and characterized (Supplementary Table 1). A promising aptamer candidate, aptscl56, was finally identified (Kd = 43.1 nM, IC_50_ = 19.7 µg/ml) as a specific in vitro pharmacologic tool (Fig. 4b). Aptscl56 showed a high binding ability to sclerostin loop3 but no binding to sclerostin loop1 and loop2 (Fig. 4c).

### The effect of targeting sclerostin loop3 on the expression of inflammatory cytokines and chemokines in vitro

MC3T3-E1 cells were transfected with Wnt and FL hSOST, followed by treatment with vehicle, aptscl56 and antibody. It was demonstrated that both Wnt signalling and osteogenic potential were significantly higher in the cells treated with aptscl56 or antibody than in cells treated with vehicle (Fig. 4d). These data demonstrated that targeting sclerostin loop3 with aptscl56 could attenuate the antagonistic effect of sclerostin on Wnt signalling to promote osteogenic potential in MC3T3-E1 cells.

RAW264.7 macrophages and VSMCs were treated with conditioned media from the MC3T3-E1 cells transfected with Wnt and FL hSOST, followed by treatment with vehicle, aptscl56 and antibody and then cultured in medium with AngII for 24 h. Compared to those in the macrophages treated with vehicle, the mRNA levels of *IL-6*, *TNFα* and *MCP-1* were significantly higher in the macrophages treated with antibody but were not altered in the macrophages treated with aptscl56 (Fig. 4e). Consistent results were shown in VSMCs (Fig. 4f). These data consistently suggested that sclerostin loop3 inhibition by the specific in vitro pharmacologic tool did not affect the suppressive effect of sclerostin on the expression of inflammatory cytokines and chemokines in macrophages or the expression of inflammatory chemokines in the VSMCs treated with AngII.

### Functional sites on sclerostin loop2 and loop3

The functional sites on sclerostin loop2 and loop3 were determined by using mutational methods. Three mutants were identified for the following studies: the sclerostin loop3 mutant (loop3m: IPDAAAAAAAQLLCPGAAAPRAAAARLVAS), which could bind to aptscl56 but not antagonize Wnt signalling (Supplementary Fig. 6); the aptscl56 mutant (aptscl56m: CGGGGTGTGGGTAAATCGTTAGAAAGATTAAACAGCTGCC), which could not bind to sclerostin (Supplementary Fig. 7); and the loop2 mutant (loop2m: GPARLLPNAIGRAAAWRPSGPDFR), which could bind to the sclerostin antibody but not suppress the expression of proinflammatory cytokines (Supplementary Fig. 8).

### Chemical modifications on aptscl56

Chemical modifications were performed to improve the serum stability of aptscl56 (Supplementary Fig. 9a). The unmodified aptscl56 was fully degraded at 48 h in 10% serum and 8 h in 100% serum. The modified aptscl56 remained undegraded for 72 h in 10% serum and started to be degraded at 12 h in 100% serum. At 72 h, a small amount of modified aptscl56 remained in 100% serum (Supplementary Fig. 9b). Compared to that of the unmodified aptscl56, the binding affinity and in vitro inhibitory potency of the modified aptscl56 were both improved (Supplementary Fig. 9c, d).

### Pharmacokinetics and distribution of Apc001PE in vivo

For a specific in vivo pharmacologic tool, the modified aptscl56 was conjugated to polyethylene glycol (PEG40k-aptscl56, named Apc001PE) to protect against renal filtration in vivo. The tissue distribution of Apc001PE was determined in mice (Supplementary Fig. 10). Apc001PE mainly accumulated in the liver and kidney rather than other tissues, indicating that Apc001PE was metabolized through the liver and kidney. Furthermore, the pharmacokinetic parameters of aptscl56 and Apc001PE were analysed by determining the plasma concentrations in Sprague-Dawley (SD) rats (Supplementary Fig. 11). It was demonstrated that nonconjugated aptscl56 had a short half-life (Elimination T_1/2_ = 1.8 h) and a rapid clearance rate in the circulation (apparent clearance, CL/F = 0.004 L/h/kg; area under the curve, AUC = 1205 (µg h)/ml). In comparison, Apc001PE showed a 37-fold longer half-life (Elimination T_1/2_ = 66.9 h) and a much lower clearance rate (CL/F = 0.001 L/h/kg; AUC = 11802 (µg*h)/ml) in vivo. Therefore, PEG40k conjugation dramatically increased the half-life and decreased the clearance rate of aptscl56 in vivo. Apc001PE could be used as a specific in vivo pharmacologic tool.

### Immunogenicity and toxicity of Apc001PE in rats

To evaluate the immunogenicity of the specific in vivo pharmacologic tool Apc001PE in rats, we determined the serum cytokine levels in healthy SD rats after Apc001PE administration (12 mg/kg, twice/week for six weeks). There were no significant differences in the serum levels of cytokines among the groups (Supplementary Fig. 12).

To evaluate the toxicity of Apc001PE in healthy SD rats, we determined the liver and kidney function indices and haematologic parameters by biochemical and haematological assays in rats with a single (3, 6, 12, and 24 mg/kg, respectively) and multiple administration(s) (12 mg/kg, twice/week for six weeks) of Apc001PE. There were no significant differences in the serum levels of the liver/kidney function indices or haematologic parameters among the groups (Supplementary Fig. 13).

### The effect of targeting sclerostin loop3 on cardiovascular events in *ApoE*^*−/−*^ mice with AngII infusion

**Fig. 5:**
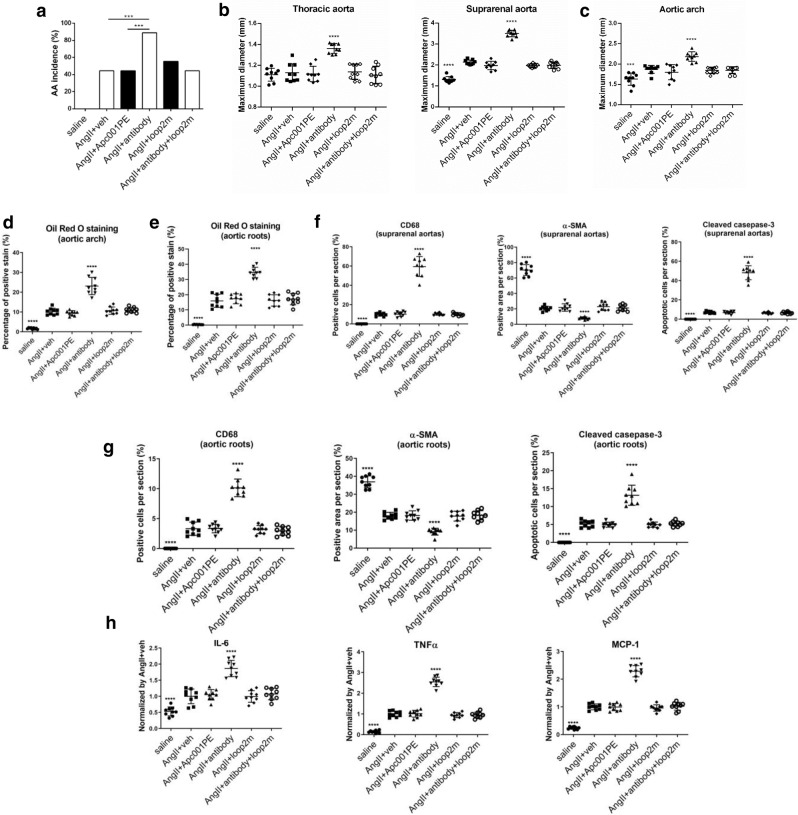
Evaluation of whether targeting sclerostin loop3 by the specific in vivo pharmacologic tool Apc001PE had an effect on cardiovascular events in *ApoE*^*−/−*^ mice with AngII infusion. *ApoE*^*−/−*^ mice with AngII infusion were subcutaneously administered vehicle, Apc001PE (12 mg/kg), humanized therapeutic sclerostin antibody (25 mg/kg), fatty acid-loop2m (6 mg/kg), or humanized therapeutic sclerostin antibody and fatty acid-loop2m (6 mg/kg) twice weekly for four weeks. **a** The incidence of AA formation. A two-sided chi-square test was performed to determine the difference between groups. ***p < 0.005*. p* = 0.0002 for all comparisons. **b** Ex vivo measurement of the maximum diameters of thoracic aortas and suprarenal aortas. Thoracic aorta: *p* < 0.0001 (AngII + antibody); suprarenal aorta: *p* < 0.0001 (saline), *p* < 0.0001 (AngII + antibody). **c** Ex vivo measurement of the maximum diameters of the aortic arches. *p* = 0.0007 (saline), *p* < 0.0001 (AngII + antibody). **d** Oil Red O staining of the aortic arch to quantify atherosclerosis. *p* < 0.0001 (saline), *p* < 0.0001 (AngII + antibody). **e** Quantification of positive staining per cryosection of aortic roots stained with Oil Red O. *p* < 0.0001 (saline), *p* < 0.0001 (AngII + antibody). **f**, **g** Quantification of the expression of CD68, α-SMA and cleaved caspase-3 in suprarenal aortas (**f**) and aortic roots (**g**) by immunohistochemical analysis. For (**f**): CD68: *p* = 0.0001 (saline), *p* < 0.0001 (AngII + antibody); α-SMA: *p* = 0.0001 (saline), *p* < 0.0001 (AngII + antibody); cleaved caspase-3: *p* < 0.0001 (saline), *p* < 0.0001 (AngII + antibody). For (**g**): CD68: *p* < 0.0001 (saline), *p* < 0.0001 (AngII + antibody); α-SMA: *p* < 0.0001 (saline), *p* < 0.0001 (AngII + antibody); cleaved caspase-3: *p* < 0.0001 (saline), *p* < 0.0001 (AngII + antibody). **h** Serum levels of inflammatory cytokines and chemokines. IL-6: *p* < 0.0001 (saline), *p* < 0.0001 (AngII + antibody); TNF-α: *p* < 0.0001 (saline), *p* < 0.0001 (AngII + antibody); MCP-1: *p* < 0.0001 (saline), *p* < 0.0001 (AngII + antibody). For (**b**–**h**), data were expressed as the mean ± standard deviation. *n* = 9 per group. *p < 0.05, **p < 0.01, ***p < 0.005, and ****p < 0.0001 for a comparison vs. AngII+veh by one-way ANOVA with Tukey’s post-hoc test. Note: AngII: angiotensin II; IL-6: interleukin 6; MCP-1: monocyte chemoattractant protein-1; TNF-α: tumour necrosis factor alpha; CD68: macrophage biomarker; α-SMA: contractile cell biomarker; cleaved caspase-3: apoptotic cell biomarker; loop2m: the loop2 mutant (GPARLLPNAIGRAAAWRPSGPDFR). Source data are provided as a Source Data file.

To evaluate whether targeting sclerostin loop3 by Apc001PE could influence the cardiovascular system, we determined the parameters for both AA and atherosclerotic development in the *ApoE*^*−/−*^ mice with AngII infusion after administration of vehicle (veh), Apc001PE (25 mg/kg, twice/week), or sclerostin antibody (25 mg/kg, twice/week) for four weeks^7^. Compared to those in the veh group, the AA incidences, the maximum ex vivo diameters of thoracic aortas and suprarenal aortas, the maximum ex vivo diameters of aortic arches, the atherosclerotic plaque area/total aortic arch area, and the atherosclerotic lesion area/total cross cryosection area of the aortic root were not altered in the AngII+Apc001PE group but were higher in the AngII+antibody group (Fig. 5a–e, Supplementary Fig. 14a–c).

Immune cell infiltration^11,15^, contractile phenotype loss of aortic VSMCs and cell apoptosis^11,16^ were involved in AA and atherosclerotic development. Compared to those in the AngII+veh group, the numbers of CD68-positive macrophages, α-SMA-positive contractile VSMCs, and cleaved caspase-3-positive apoptotic cells in the suprarenal aortas and aortic roots were not altered in the AngII+Apc001PE group but were significantly higher in the AngII+antibody group (Fig. 5f, g, Supplementary Fig. 14d-e). Furthermore, compared to those in the AngII+veh group, the serum levels of IL-6, TNF-α and MCP-1 were not altered in the AngII+Apc001PE group but were significantly higher in the AngII+antibody group (Fig. 5h).

Mechanistically, to further explore how therapeutic antibody targeting both sclerostin loop2 and loop3 increased cardiovascular risk, we determined all of the above parameters in the *ApoE*^*−/−*^ mice with AngII infusion after administration of therapeutic antibody with and without pretreatment with loop2m (6 mg/kg, once weekly). It was found that the increased cardiovascular risk of the therapeutic antibody could be abolished by supplementation with exogenous loop2m (Fig. 5, Supplementary Fig. 14). These data further indicated that sclerostin loop2, rather than loop3, could participate in sclerostin’s protective effect on the cardiovascular system. Thus, targeting sclerostin loop3 by the specific in vivo pharmacologic tool Apc001PE had no effect on cardiovascular events in the *ApoE*^*−/−*^ mice with AngII infusion.

### The effect of targeting sclerostin loop3 by Apc001PE on the cardiovascular system in the *hSOST*^*ki*^*.ApoE*^*−/−*^ mice with AngII infusion

**Fig. 6:**
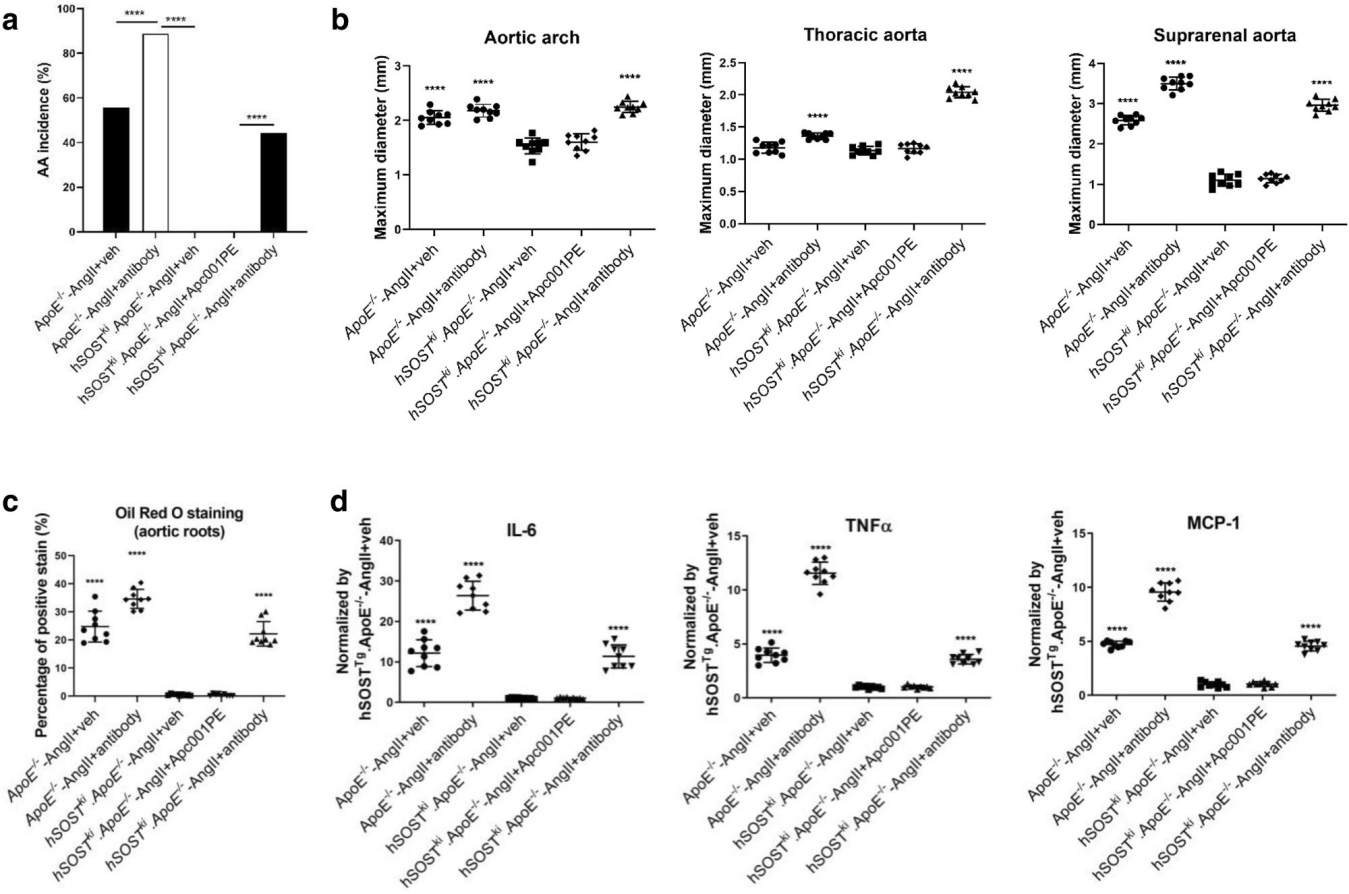
Determination of whether targeting sclerostin loop3 by the specific in vivo pharmacologic tool Apc001PE influenced the protective effect of overexpressed human sclerostin on the cardiovascular system by evaluating cardiovascular events in *hSOST*^*ki*^*.ApoE*^*−/−*^ mice with AngII infusion. *ApoE*^*−/−*^ mice or *hSOST*^*ki*^*. ApoE*^*−/−*^ mice with AngII infusion were subcutaneously administered vehicle, Apc001PE (12 mg/kg) or humanized therapeutic sclerostin antibody (25 mg/kg) twice weekly for four weeks. After administration, the aortas were harvested for analysis. **a** The incidence of AA formation. A two-sided chi-square test was performed to determine the difference between groups. ****p < 0.0001*. p* < 0.0001 for all comparisons. **b** Ex vivo measurement of the maximum diameters of the aortic arches (left), thoracic aortas (middle) and suprarenal aortas (right). Aortic arch: *p* < 0.0001 (*ApoE*^*−/−*^-AngII+veh), *p* < 0.0001 (*ApoE*^*−/−*^-AngII+antibody), *p* < 0.0001 (*hSOST*^*ki*^*. ApoE*^*−/−*^-AngII+antibody); thoracic aorta:, *p* < 0.0001 (*ApoE*^*−/−*^-AngII+antibody), *p* < 0.0001 (*hSOST*^*ki*^*. ApoE*^*−/−*^-AngII+antibody); suprarenal aorta: *p* < 0.0001 (*ApoE*^*−/−*^-AngII+veh), *p* < 0.0001 (*ApoE*^*−/−*^-AngII+antibody), *p* < 0.0001 (*hSOST*^*ki*^*. ApoE*^*−/−*^-AngII+antibody). **c** Quantification of positive staining per cryo-section of aortic roots stained with Oil Red O. *p* < 0.0001 (*ApoE*^*−/−*^-AngII+veh), *p* < 0.0001 (*ApoE*^*−/−*^-AngII+antibody), *p* < 0.0001 (*hSOST*^*ki*^*. ApoE*^*−/−*^-AngII+antibody). **d** Serum levels of inflammatory cytokines (IL-6, TNF-α) and chemokines (MCP-1). IL = 6: *p* < 0.0001 (*ApoE*^*−/−*^-AngII+veh), *p* < 0.0001 (*ApoE*^*−/−*^-AngII+antibody, *p* < 0.0001 (*hSOST*^*ki*^*. ApoE*^*−/−*^-AngII+antibody); TNF-α: *p* < 0.0001 (*ApoE*^*−/−*^-AngII+veh), *p* < 0.0001 (*ApoE*^*−/−*^-AngII+antibody), *p* < 0.0001 (*hSOST*^*ki*^*. ApoE*^*−/−*^-AngII+antibody); MCP-1: *p* < 0.0001 (*ApoE*^*−/−*^-AngII+veh), *p* < 0.0001 (*ApoE*^*−/−*^-AngII+antibody), *p* < 0.0001 (*hSOST*^*ki*^*. ApoE*^*−/−*^-AngII+antibody). For (**b** to **d**), data were expressed as the mean ± standard deviation. *n* = 9 per group. ****p < 0.0001 for a comparison *vs. hSOST*^*ki*^*. ApoE*^*−/−*^-AngII+veh by one-way ANOVA with Tukey’s post-hoc test. Note: AngII: Angiotensin II; IL-6: interleukin 6; MCP-1: monocyte chemoattractant protein-1; TNF-α: tumour necrosis factor alpha. Source data are provided as a Source Data file.

Transgenic introduction of sclerostin protected against AngII-induced AA and atherosclerotic progression^7^. The *ApoE*^*−/−*^ or *hSOST*^*ki*^*. ApoE*^*−/−*^ mice were treated with vehicle (veh), Apc001PE (25 mg/kg, twice/week) or sclerostin antibody (25 mg/kg, twice/week) for four weeks during AngII infusion. It was demonstrated that the AA incidence, the maximum ex vivo diameters of aortic arches, thoracic aortas and suprarenal aortas, atherosclerotic plaque area/total *en face* aortic arch area, atherosclerotic lesion area/total cross cryosection area of the aortic root, and serum levels of inflammatory cytokines and chemokines were significantly reduced in the *hSOST*^*ki*^*.ApoE*^*−/−*^-AngII+veh group compared to the *ApoE*^*−/−*^-AngII+veh group, validating the protective effect of sclerostin on the cardiovascular system (Fig. 6, Supplementary Fig. 15). Compared to those in the *hSOST*^*ki*^*.ApoE*^*−/−*^-AngII+veh group, the above parameters were not altered in the *hSOST*^*ki*^*.ApoE*^*−/−*^-AngII+Apc001PE group and were significantly elevated in the *hSOST*^*ki*^*.ApoE*^*−/−*^-AngII+antibody group. These data demonstrated that targeting sclerostin loop3 by the specific in vivo pharmacologic tool Apc001PE could maintain the protective effect of sclerostin on the cardiovascular system in the *hSOST*^*ki*^*.ApoE*^*−/−*^ mice with AngII infusion.

### The effect of targeting sclerostin loop3 with Apc001PE on bone formation in the *hSOST*^*ki*^ mice in vivo

To examine whether targeting sclerostin loop3 by the specific in vivo pharmacologic tool Apc001PE could attenuate the inhibitory effect of sclerostin on bone formation in *hSOST*^*ki*^ mice, mice were grouped and treated accordingly (Supplementary Fig. 16a).

**Fig. 7:**
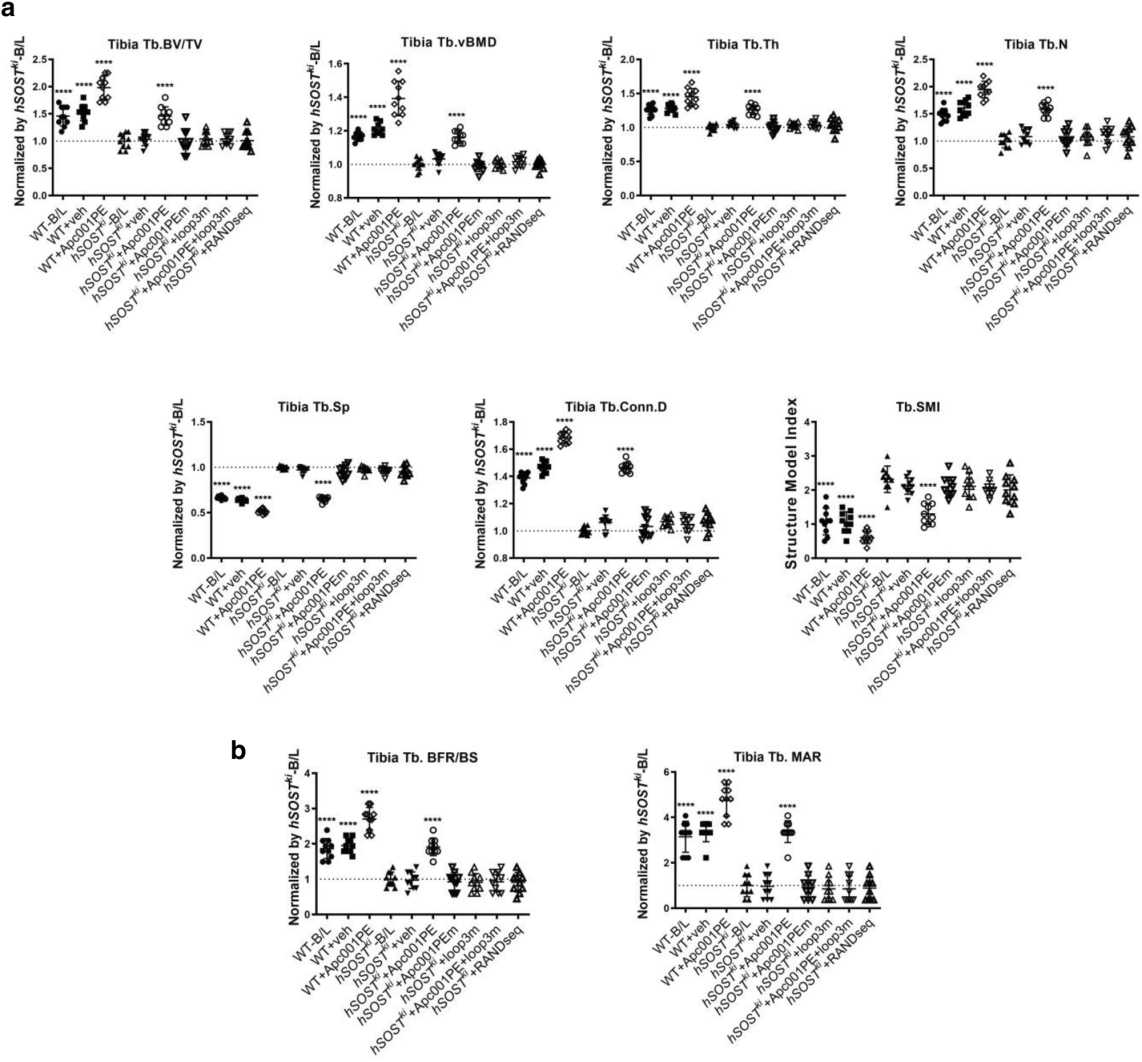
Evaluation of whether targeting sclerostin loop3 by the specific in vivo pharmacologic tool Apc001PE could attenuate the inhibitory effect of sclerostin on bone formation in *hSOST*^*ki*^ mice. **a** Bar charts of the structural parameters from ex vivo micro-CT examination at the proximal tibia. Tb.BV/TV: *p* < 0.0001 (WT-B/L, WT + veh, WT + Apc001PE, *hSOST*^*ki*^ + Apc001PE); Tb.vBMD: *p* < 0.0001 (WT-B/L, WT + veh, WT + Apc001PE, *hSOST*^*ki*^ + Apc001PE); Tb.Th: *p* < 0.0001 (WT-B/L, WT + veh, WT + Apc001PE, *hSOST*^*ki*^ + Apc001PE), Tb.N: *p* < 0.0001 (WT-B/L, WT + veh, WT + Apc001PE, *hSOST*^*ki*^ + Apc001PE); Tb.Sp: *p* < 0.0001 (WT-B/L, WT + veh, WT + Apc001PE, *hSOST*^*ki*^ + Apc001PE); Tb.ConnD: *p* < 0.0001 (WT-B/L, WT + veh, WT + Apc001PE, *hSOST*^*ki*^ + Apc001PE); Tb.SMI: *p* < 0.0001 (WT-B/L, WT + veh, WT + Apc001PE, *hSOST*^*ki*^ + Apc001PE). **b** Analysis of dynamic bone histomorphometric parameters at the proximal tibia. Tb.BFR/BS: *p* < 0.0001 (WT-B/L, WT + veh, WT + Apc001PE, *hSOST*^*ki*^ + Apc001PE); Tb.MAR: *p* < 0.0001 (WT-B/L, WT + veh, WT + Apc001PE, *hSOST*^*ki*^ + Apc001PE). For (**a** and **b**), data were expressed as the mean ± standard deviation followed by one-way ANOVA with Tukey’s post-hoc test *vs*. *hSOST*^*ki*^-B/L, *n* = 10 per group. *p < 0.05; **p < 0.01; ***p < 0.005; ****p < 0.0001. Note: Apc001PEm: PEGylated aptscl56 mutant with T13A, C14A, G15A, C23A, T24A, T25A, T30A, G31A and G32A mutations; Tb.BV/TV: trabecular relative bone volume; Tb.vBMD: trabecular volumetric mineral density; Tb.Th: trabecular thickness; Tb.N: trabecular number; Tb.Sp: trabecular spacing; Tb.conn.D: trabecular connection density; Tb.SMI: trabecular structure model index; Tb.BFR/BS: bone formation rate; Tb.MAR: the mineral apposition rate. Source data are provided as a Source Data file.

After treatment, the micro-CT data showed that the *hSOST*^*ki*^ + Apc001PE group had significantly higher Tb.BV/TV, Tb.vBMD, Tb.Th, Tb.N, and Tb.conn.D values and lower Tb.Sp values than those in the *hSOST*^*ki*^-B/L group. The Tb.SMI value was close to 0 in the *hSOST*^*ki*^ + Apc001PE group but close to 3 in the *hSOST*^*ki*^-B/L group, indicating the plate-like shape of the trabecular bone in the *hSOST*^*ki*^ + Apc001PE group and the rod-like shape of the trabecular bone in the *hSOST*^*ki*^-B/L group at the proximal tibia. There were no significant differences among the *hSOST*^*ki*^-B/L, *hSOST*^*ki*^ + Veh and *hSOST*^*ki*^ + RANDseq groups in any of the parameters (Fig. 7a, Supplementary Fig. 16b).

In bone histomorphometric analysis of the trabecular bone at the proximal tibia, the BFR/BS and MAR/BS were notably higher in the *hSOST*^*ki*^ + Apc001PE group than in the *hSOST*^*ki*^-B/L group. There were no significant differences among the *hSOST*^*ki*^-B/L, *hSOST*^*ki*^ + Veh and *hSOST*^*ki*^ + RANDseq groups in any of the parameters (Fig. 7b and Supplementary Fig. 16c). Therefore, targeting sclerostin loop3 by the specific in vivo pharmacologic tool Apc001PE could attenuate the inhibitory effect of sclerostin on bone formation in *hSOST*^*ki*^ mice.

Mechanistically, to further test how the specific in vivo pharmacologic tool Apc001PE attenuated the inhibitory effect of sclerostin on bone formation, we determined the bone anabolic potential of Apc001PE in the *hSOST*^*ki*^ mice with and without pretreatment with loop3m. The Apc001PE-mediated attenuation of sclerostin’s inhibition on bone formation could be abolished by supplementation with exogenous loop3m (Fig. 7, Supplementary Fig. 16). In contrast, the Apc001PE mutant (Apc001PEm: PEG40k-aptscl56m) was administered to *hSOST*^*ki*^ mice. There were no significant differences between the *hSOST*^*ki*^ + Apc001PEm group and the *hSOST*^*ki*^ + veh group in any of the above parameters for both micro-CT and bone histomorphometric analysis. Thus, targeting sclerostin loop3 by the specific in vivo pharmacologic tool Apc001PE attenuated the inhibitory effect of sclerostin on bone formation by neutralizing circulating sclerostin loop3 in *hSOST*^*ki*^ mice in vivo.

### The effect of targeting sclerostin loop3 by Apc001PE on bone formation in osteoporotic rats induced by ovariectomy (OVX)

To examine whether targeting sclerostin loop3 by the specific in vivo pharmacologic tool Apc001PE could promote bone formation, increase bone mass, and improve bone microarchitecture integrity in rats with established osteoporosis induced by OVX, SD rats were grouped and treated accordingly (Supplementary Figs. 17a and 18).

**Fig. 8:**
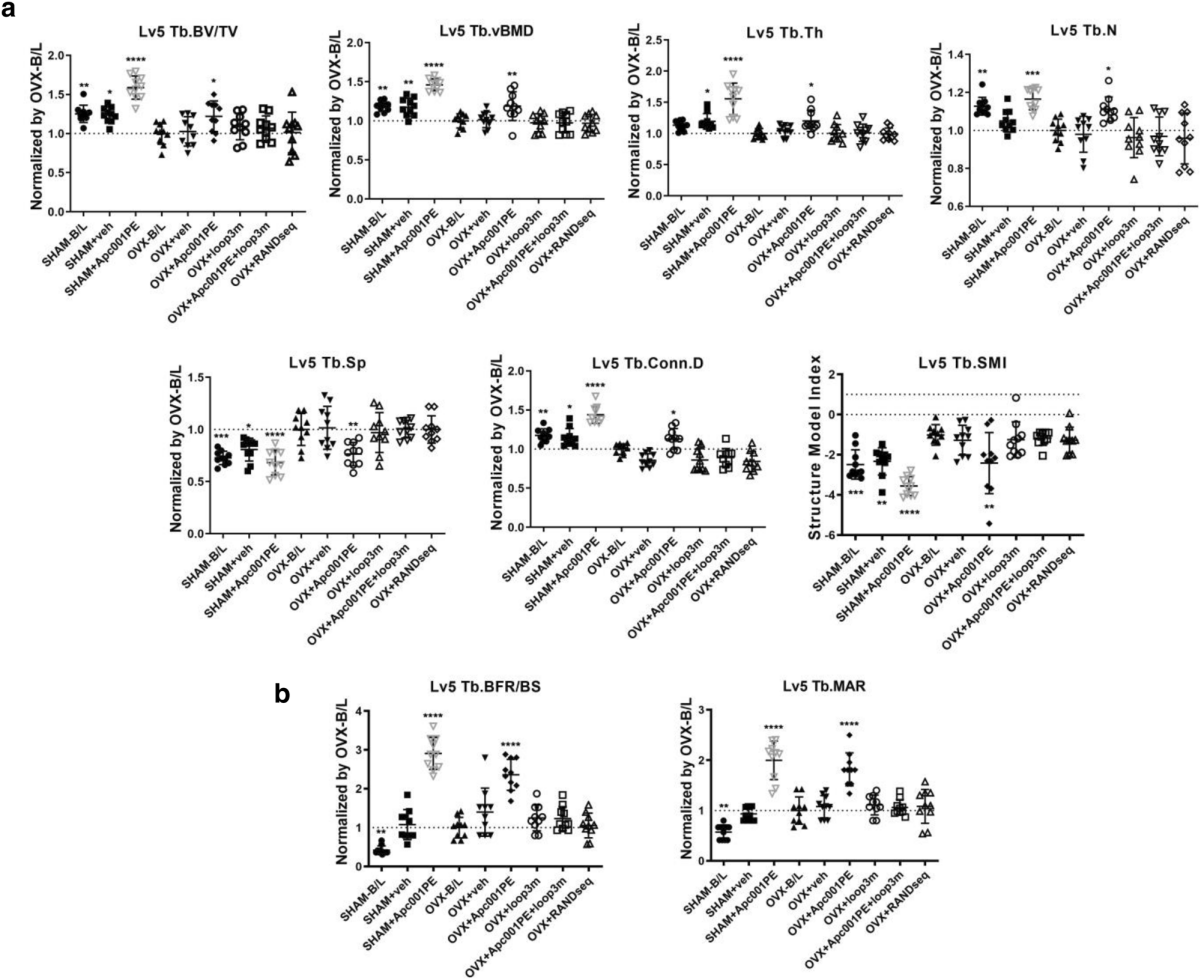
Determination of whether targeting sclerostin loop3 by the specific in vivo pharmacologic tool Apc001PE could exert bone anabolic potential by neutralizing circulating sclerostin loop3 in ovariectomy-induced (OVX) osteoporotic rats. **a** Bar charts of the structural parameters from ex vivo micro-CT at the fifth vertebrae. Tb.BV/TV: *p* = 0.0086 (SHAM-B/L), *p* = 0.0138 (SHAM + veh), *p* < 0.0001 (SHAM + Apc001PE), *p* = 0.0277 (OVX + Apc001PE); Tb.vBMD: *p* = 0.0033 (SHAM-B/L), *p* = 0.0066 (SHAM + veh), *p* < 0.0001 (SHAM + Apc001PE), *p* = 0.0028 (OVX + Apc001PE); Tb.Th: *p* = 0.0169 (SHAM + veh), *p* < 0.0001 (SHAM + Apc001PE), *p* = 0.0116 (OVX + Apc001PE); Tb.N: *p* = 0.0084 (SHAM-B/L), *p* = 0.0003 (SHAM + Apc001PE), *p* = 0.0269 (OVX + Apc001PE); Tb.Sp: *p* = 0.0002 (SHAM-B/L), *p* = 0.0161 (SHAM + veh), *p* < 0.0001 (SHAM + Apc001PE), *p* = 0.0017 (OVX + Apc001PE); Tb.ConnD: *p* = 0.0050 (SHAM-B/L), *p* = 0.0225 (SHAM + veh), *p* < 0.0001 (SHAM + Apc001PE), *p* = 0.0328 (OVX + Apc001PE); Tb.SMI: *p* = 0.0006 (SHAM-B/L), *p* = 0.0028 (SHAM + veh), *p* < 0.0001 (SHAM + Apc001PE), *p* = 0.0012 (OVX + Apc001PE). **b** Analysis of dynamic bone histomorphometric parameters at the fifth vertebrae. Tb.BFR/BS: *p* = 0.0056 (SHAM-B/L), *p* < 0.0001 (SHAM + Apc001PE), *p* < 0.0001 (OVX + Apc001PE); Tb.MAR: *p* = 0.0024 (SHAM-B/L), *p* < 0.0001 (SHAM + Apc001PE), *p* < 0.0001 (OVX + Apc001PE). For (**a** and **b**), data were expressed as the mean ± standard deviation followed by one-way ANOVA with Tukey’s post-hoc test vs. OVX-B/L, *n* = 10 per group. *p < 0.05; **p < 0.01; ***p < 0.005; ****p < 0.0001. Note: Loop3m: the loop3 mutant (IPDAAAAAAAQLLCPGAAAPRAAAARLVAS); Tb.BV/TV: trabecular relative bone volume; Tb.vBMD: trabecular volumetric mineral density; Tb.Th: trabecular thickness; Tb.N: trabecular number; Tb.Sp: trabecular spacing; Tb.conn.D: trabecular connection density; Tb.SMI: trabecular structure model index; Tb.BFR/BS: trabecular bone formation rate; Tb.MAR: trabecular mineral apposition rate. Source data are provided as a Source Data file.

Micro-CT analysis was performed for the trabecular bone (below the growth plate) at the metaphysis of the distal femur, the proximal tibia and the fifth lumbar vertebrae, as well as the cortical bone at the femoral mid-shaft. After treatment, the OVX + Apc001PE group had significantly higher Tb.BV/TV, Tb.vBMD, Tb.Th, Tb.N and Tb.Conn.D values but lower Tb.Sp values than the OVX-B/L group, indicating a notably increased trabecular bone mass in the OVX rats treated with Apc001PE. The Tb.SMI was close to 0 in the OVX + Apc001PE group but close to 3 in the OVX-B/L group, indicating the plate-like shape of trabecular bone in the OVX + Apc001PE group and the rod-like shape of trabecular bone in the OVX-B/L group at the fifth lumbar vertebrae (Fig. 8a and Supplementary Fig. 17b). Micro-CT data for the distal femur (Supplementary Fig. 19a) and the proximal tibia (Supplementary Fig. 19b) showed consistent results. Furthermore, micro-CT of the trabecular region of the above three sites showed no significant differences among the OVX + veh, OVX-B/L and OVX + RANDseq groups in any of the parameters. For the femoral mid-shaft, the OVX + Apc001PE group had higher cortical thickness (Ct.Th) than the OVX-B/L group. There were no significant differences among the OVX + veh, OVX-B/L and OVX + RANDseq groups for the above parameters (Supplementary Fig. 19c).

Bone histomorphometric analysis was performed on the trabecular bone at the above three sites to further examine whether targeting sclerostin loop3 by the specific in vivo pharmacologic tool Apc001PE could promote bone formation in OVX rats. For the fifth lumbar vertebrae, the bone histomorphometric analysis showed that Tb.BFR/BS and Tb.MAR were notably higher in the OVX + Apc001PE group than in the OVX-B/L group (Fig. 8b and Supplementary Fig. 17c). The bone histomorphometric data of the distal femur (Supplementary Fig. 20a) and the proximal tibia (Supplementary Fig. 20b) showed consistent results. The bone histomorphometric analysis of the trabecular region at the above three sites showed no significant differences among the OVX + veh, OVX-B/L and OVX + RANDseq groups in any of the parameters. For the femoral mid-shaft, the bone histomorphometric analysis showed that Ct.BFR/BS and Ct.MAR were notably higher in the OVX + Apc001PE group than in the OVX-B/L group (Supplementary Fig. 20c).

To further examine whether targeting sclerostin loop3 by the specific in vivo pharmacologic tool Apc001PE could enhance the bone mechanical properties in rats with established osteoporosis induced by OVX, we used the compression test for measurement of the fourth lumbar vertebrae. The failure force and ultimate strength were significantly higher in the OVX + Apc001PE group than in the OVX-B/L group. There were no significant differences among the OVX + veh, OVX-B/L and OVX + RANDseq groups in any of the above parameters (Supplementary Fig. 21a). Furthermore, the three-point bending test was used for measurement of the femoral midshaft. The stiffness, failure force and fracture energy were significantly higher in the OVX + Apc001PE group than in the OVX-B/L group. There were no significant differences among the OVX + veh, OVX-B/L and OVX + RANDseq groups in any of the above parameters (Supplementary Fig. 21b).

The serum levels of the bone formation biomarker procollagen type 1 N-terminal propeptide (P1NP) were determined. The serum level of P1NP was significantly higher in the OVX + Apc001PE group than in the OVX-B/L group. There were no significant differences among the OVX + veh, OVX-B/L and OVX + RANDseq groups (Supplementary Fig. 21c). Furthermore, there were no significant differences in the serum levels of sclerostin among all groups after treatments (Supplementary Fig. 21d).

For molecular analysis, OVX-induced osteoporotic rats were treated with Apc001PE with and without pretreatment with loop3m. The above bone anabolic effects of Apc001PE could be abolished by supplementation with exogenous loop3m (Fig. 8 and Supplementary Fig. 17–21). Thus, targeting sclerostin loop3 by the in vivo pharmacologic tool Apc001PE promoted bone formation, increased bone mass, improved bone microarchitecture integrity and enhanced bone mechanical properties by neutralizing circulating sclerostin loop3 in osteoporotic rats induced by OVX.

## Discussion

This study found that sclerostin loop3 cannot participate in protecting the cardiovascular system but is involved in inhibiting bone formation by genetic and pharmacologic approaches both in vitro and in vivo.

In our in vitro studies, either loop3 deficiency by genetic truncation or targeting sclerostin loop3 by the specific in vitro pharmacologic tool aptacl56 maintained the suppressive effect of sclerostin on the expression of inflammatory cytokines and chemokines in AngII-treated macrophages and VSMCs. In our in vivo studies, either loop3 deficiency by genetic truncation or targeting sclerostin loop3 by the specific in vivo pharmacologic tool Apc001PE maintained the protective effect of sclerostin on the cardiovascular system in *ApoE*^*−/−*^ mice with AngII infusion. The above data consistently indicated that loop3 deficiency by genetic truncation or targeting sclerostin loop3 by the specific pharmacologic tool had no influence on cardiovascular events both in vitro and in vivo.

In our in vitro studies, either loop2&3 deficiency by genetic truncation or loop2&3 inhibition by sclerostin antibody attenuated the suppressive effect of sclerostin on the expression of inflammatory cytokines and chemokines in AngII-treated macrophages and VSMCs. In our in vivo studies, loop2&3 inhibition by sclerostin antibody significantly accelerated the development of cardiovascular events in *ApoE*^*−/−*^ mice with AngII infusion. In a phase III clinical trial (BRIDGE), the total incidence of treatment-emergent cardiovascular serious adverse events was reported to be significantly higher in the antibody group than in the placebo group during the first 12-month period^17^. Furthermore, in a phase III clinical trial (ARCH), the incidence of treatment-emergent cardiac ischaemic events was significantly higher in the antibody group than in the alendronate (ALN) group during the 12-month double-blind period^18^. Meta-analysis showed that ALN did not have a protective effect on the cardiovascular system^19^, suggesting that the cardiovascular risk of therapeutic antibody against sclerostin still needs to be addressed. Moreover, a meta-analysis for phase III clinical trials further confirmed the higher risk of cardiac ischaemic events in patients randomized to antibody treatment^6,20^. It was also found that two independent BMD-increasing SOST variants (rs7209826 and rs188810925), whose SOST expression levels were lower than those of the wild-type groups, were associated with a higher cardiovascular risk. Therefore, both genetically and therapeutically lowered sclerostin led to a higher risk of cardiovascular events^6,20^. These data from clinical trials and our data consistently indicated that loop2&3 inhibition by sclerostin antibody could attenuate the protective effect of sclerostin on the cardiovascular system.

In one nonclinical evaluation of cardiovascular events, *ApoE*^*−/−*^ mice were fed a high-fat diet for 2 weeks, followed by OVX. Two weeks after OVX, mice were subcutaneously administered a sclerostin antibody (r13C7 [Amgen, Inc.]) once weekly at a dose of 10 mg/kg (Multi-Discipline Review, Amgen Study No. 124609). After treatment, inflammatory cytokines and chemokines, including IL-6 and MCP-1, were locally upregulated in the aorta. In addition, sclerostin antibody enhanced the incidence of atherosclerotic plaques of types 2–5 with necrosis, indicating a potential concern of increasing plaque instability. Consistently, in our studies in *ApoE*^*−/−*^ mice with AngII infusion, the serum levels of inflammatory cytokines and chemokines were significantly higher in the sclerostin antibody group (25 mg/kg, twice/week) than in the vehicle group. Similarly, the ratio of atherosclerotic plaques in the aortic arches and aortic roots was significantly higher in the sclerostin antibody group than in the vehicle group. Moreover, our data showed that targeting sclerostin loop2&3 with a sclerostin antibody increased AA incidence and accelerated AA development in the *ApoE*^*−/−*^ mice with AngII infusion. Nevertheless, another group reported that sclerostin antibody (10 mg/kg, once weekly) did not influence the total atherosclerotic plaque volume or the circulating concentrations of IL-6, TNF-α and MCP-1 in *ApoE*^*−/−*^ mice with AngII infusion^21^. The lack of effect on cardiovascular events could be explained by the lower dose employed in the literature (10 mg/kg per week)^21^ than the higher dose employed in our study (50 mg/kg per week).

In our genetic truncation studies, loop3 deficiency by genetic truncation attenuated the inhibitory effect of sclerostin on Wnt signalling and osteogenic potential in MC3T3-E1 cells in vitro. In studies of knock-in mice, loop3 deficiency by genetic truncation attenuated the inhibitory effect of human sclerostin on bone formation in vivo. Targeting sclerostin loop3 by the specific in vitro pharmacologic tool aptscl56 inhibited sclerostin’s antagonistic effect on Wnt signalling and osteogenic potential in MC3T3-E1 cells in vitro. Moreover, targeting sclerostin loop3 by the specific in vivo pharmacologic tool Apc001PE attenuated the inhibitory effect of sclerostin on bone formation in the *hSOST*^*ki*^ mice and promoted bone formation in the OVX-induced osteoporotic rats in vivo. The above in vitro and in vivo data consistently demonstrated the promising bone anabolic potential of targeting sclerostin loop 3 with a specific pharmacologic tool.

In our in vitro mutation studies, sclerostin loop3m failed to inhibit Wnt signalling but remained bound to aptscl56. The aptscl56m failed to bind to sclerostin. In our in vivo studies, the attenuation of the specific in vivo pharmacologic tool Apc001PE on sclerostin’s bone formation inhibition could be completely abolished by supplementation with exogenous loop3m in *hSOST*^*ki*^ mice. Moreover, the bone anabolic potential of the specific in vivo pharmacologic Apc001PE could be completely abolished by supplementation with exogenous loop3m in the OVX-induced osteoporotic rats. Furthermore, Apc001PEm did not attenuate sclerostin’s bone formation inhibition in *hSOST*^*ki*^ mice or the bone anabolic potential in OVX-induced osteoporotic rats. These data consistently demonstrated that targeting sclerostin loop3 by the specific in vivo pharmacologic tool Apc001PE attenuated the inhibitory effect of sclerostin on bone formation in the *hSOST*^*ki*^ mice and promoted bone formation in the OVX-induced osteoporotic rats by neutralizing the circulating sclerostin loop3.

It was reported that sclerostin loop2, especially Asn92 and Ile94, is important for antagonizing the Wnt signalling pathway^22^, which was mainly focused on the residues of sclerostin loop2 in regulating Wnt signalling. In our studies, we found that sclerostin loop3 could also participate in inhibiting Wnt signalling but not in protecting the cardiovascular system, which mainly focuses on the residues of loop3 in regulating Wnt signalling. Furthermore, this study is an attempt to study the key sites of loop3 in regulating Wnt signalling and in interacting with aptamer aptscl56. Extensive studies will be necessary to further confirm and validate the roles of these sites.

Taken together, this study validated that targeting sclerostin loop3 by genetic and pharmacologic approaches could maintain the protective effect of sclerostin on the cardiovascular system and promote bone formation. This study could provide a target for the development of next-generation sclerostin inhibitors that specifically target sclerostin loop3 for bone anabolic therapy, with a low cardiovascular risk.

## Methods

### Study approval

All procedures in the present study were approved by the Research Ethics Committee of Hong Kong Baptist University.

### Cell culture

MC3T3-E1 subclone 4 (mouse preosteoblast, ATCC CRL-2593) was cultured in α-MEM with 10% FBS and 100 units/ml penicillin-streptomycin. RAW 264.7 cells (macrophages, ATCC TIB-71) were cultured in DMEM with 10% FBS and 100 units/ml penicillin-streptomycin. Human VSMCs (aorta/smooth muscle, ATCC CRL-1999) were cultured in DMEM with 10% FBS, 0.2 mg/ml G418 and 100 units/ml penicillin-streptomycin. No mycoplasma infection was found for any of the cell cultures.

### TOP-Wnt-induced luciferase reporter assay

To assess the inhibitory potency of aptamers against sclerostin’s antagonistic effect on Wnt signalling, we performed a TOP-Wnt-induced luciferase reporter assay in preosteoblast MC3T3-E1 cells^23,24^. Cells were seeded in 24-well plates and transfected with the corresponding reporter plasmids, Wnt3a plasmid and sclerostin plasmid as necessary the following day. Six hours after transfection, the culture medium was changed to fresh medium, and the cells were treated with aptamers or the corresponding reagents. Twenty-four hours after treatment, the cells were lysed with 100 μl/well passive lysis buffer, and 20 μl was taken for analysis. Luciferase assays were performed using the Dual-Luciferase Reporter system with parameter settings according to the manufacturer’s protocol^25,26^.

### Quantitative PCR

Total RNA from cultured cells was isolated by homogenization using TRIzol (Invitrogen) and reverse transcribed into cDNA using a high-capacity cDNA reverse transcription kit (Applied Biosystems). Quantitative PCRs were performed using TaqMan Universal PCR Master Mix on the 7900 HT Sequence Detection System (Applied Biosystems). Primers were purchased from Applied Biosystems, including those for *GAPDH* (*GAPDH*: Hs99999905_m1, Mm99999915_g1), *interleukin 6* (*IL-6*: Hs00174131_m1), *CCL-2 (MCP-1*: Hs00234140_m1), *TNFα* (*TNFα*: Hs00174128_m1), *ALPL* (*ALP*: Mm00475834_m1) and *Bglap* (*OCN*: Mm03413826_mH). Relative RNA expression of genes was determined using the 2-ΔΔCt method with *GAPDH* as the endogenous normalizer^27^.

### Mice and genotyping

The procedures of all animal studies have gained ethics approval by the Research Ethics Committee of the Hong Kong Baptist University. The animals were grouped randomly and blindly to researchers. All animal experiments were carried on in compliance to the relevant ethical regulations of Hong Kong Baptist University. The animals in poor body condition were excluded. Transgenic C57BL/6 mice expressing human sclerostin (B6. *hSOST*^*ki*^), 3-month, male and female; loop3 deficient human sclerostin (B6. *Δloop3-hSOST*^*ki*^), 3-month, male and female, and wide-type C57BL/6 mice, 3-month, male and female were used to evaluate the bone anabolic potential (*n* = 10 per group). Sprague Dawley rats, 4-month, female, were used evaluate the bone anabolic potential, serum levels immune factors and toxicities (*n* = 10 per group), and to perform the pharmacokinetic analysis (*n* = 6 per group). Apolipoprotein E null (B6. *ApoE*^*−/−*^) mice (C57BL/6 J background), 3-month, male; wide-type C57BL/6 J, 3-month, male and female; *hSOST*^*ki*^*. ApoE*^*−/−*^ mice (B6.*hSOST*^*ki*^ mice crossed to B6. *ApoE*^*−/−*^ mice), 3-month, male and female; *Δloop3-hSOST*^*ki*^*. ApoE*^*−/−*^ mice (B6. *Δloop3-hSOST*^*ki*^ mice crossed to B6. *ApoE*^*−/−*^ mice), 3-month, male and female, and wide-type C57BL/6 mice, 3-month, male and female were used to evaluate the cardiovascular events (n = 9 per group). Wide-type C57BL/6 mice, 3-month, male and female were used to determine the aptamer distribution in organs (n = 6 per group).

All animals were maintained under a 12 h light-12 h dark cycle in a temperature-controlled room with ad libitum access to water and food throughout the experimental period. 3-month-old male apolipoprotein E null (B6.*ApoE*^−/−^) mice were purchased from the Laboratory Animal Services Centre, The Chinese University of Hong Kong. Full-length human sclerostin knock-in (B6.*hSOST*^*ki*^) and loop3-deficient human sclerostin knock-in (B6.*Δloop3-hSOST*^*ki*^) mice were constructed in collaboration with GemPharmatech Co., Ltd, China. Briefly, the *CAG-LSL-hSOST/Δloop3-hSOST(1-110)-tdTomato* gene fragment was inserted into the mouse (C57BL/6 J) H11 site on chromosome 11 by CRISPR/Cas9 with the CAG promoter. The F0-positive mice mated with C57BL/6 J mice were verified by PCR and sequencing to obtain stable F1-positive mouse models^28,29^. The *hSOST* gene was genotyped using DNA extracted from mouse tail-clippings, amplified using the 5ʹ arm forward primer (FP) 5-ATGCCCACCAAAGTCATCAGTGTAG-3 the 5ʹ arm reverse primer (RP) 5ʹ-AGGCGGGCCATTTACCGTAAGTTA-3ʹ, 3ʹ arm FP 5’-CCTCCTCTCCTGACTACTCCCAGTC-3ʹ, and 3ʹ arm RP 5ʹ-TCACAGAAACCATATGGCGCTCC-3ʹ to generate 1465 bp (5ʹ arm) and 1229 bp (3ʹ arm) amplicons. The Δloop3-hSOST gene was genotyped using DNA extracted from mouse tail clippings and amplified using the 5ʹ arm FP 5ʹ-ATGCCCACCAAAGTCATCAGTGTAG-3ʹ, the 5ʹ RP 5ʹ-AGGCGGGCCATTTACCGTAAGTTA-3ʹ, the 3ʹ arm FP 5ʹ-CCTCCTCTCCTGACTACTCCCAGTC-3ʹ, and the 3ʹ arm RP 5ʹ-TCACAGAAACCATATGGCGCTCC-3ʹ to generate 1465 bp (5ʹ arm) and 1229 bp (3ʹ arm) amplicons. The *hSOST*^*ki*^*. ApoE*^*−/−*^ mice were generated by crossing B6.*hSOST*^*ki*^ mice with B6. *ApoE*^*−/−*^ mice. The *Δloop3-SOST*^*ki*^*.ApoE*^*−/−*^ mice were generated by crossing B6.*Δloop3-SOST*^*ki*^ mice with B6.*ApoE*^*−/−*^ mice. The *ApoE*^−/−^ allele was genotyped using DNA extracted from mouse tail-clippings and amplified using FP 5ʹ-GCCTAGCCGAGGGAGAGCCG-3ʹ and RP 5ʹ-TGTGACTTGGGAGCTCTGCAGC & GCCGCCCCGACTGCATCT-3ʹ to generate 155 bp (wild type) or 245 bp (homozygous) amplicons. Western blot and IHC were performed to determine the expression of sclerostin in tissues using anti-sclerostin antibody (Abcam, ab85799/ab63097, 1:1000).

### AA and atherosclerosis evaluation

For infusion, osmotic minipumps (Model 1004, ALZET, Durect Corporation, USA) were implanted into the subcutaneous space along the dorsal midline on the right flank via an incision in the scapular region under anaesthesia (4% isoflurane)^30,31^ to deliver AngII (500 ng/kg/min, Sigma–Aldrich) or vehicle in 3-month mice for four weeks. Two days after minipump implantation, mice were subcutaneously administered saline, therapeutic antibody against sclerostin (Hongmed-Infagen/Creative Biolabs, 25 mg/kg, twice/week) or Apc001PE (25 mg/kg, twice/week). The sequence of the humanized therapeutic sclerostin antibody employed in this study was the same as the sequence of romosozumab (EVENITY™ [romosozumab-aqqg in the US]). The administration dose of Apc001PE referred to the mass of aptscl56. The body weight of each mouse was recorded. For AA evaluation, the aortas were perfused via the left ventricle with ice-cold saline immediately after sacrifice. The aortas were then isolated from the fat and connective tissues under a Zeiss Stemi 305 stereomicroscope with an AxioCam 208 Color Camera and then fixed in 4% PFA. The aorta with or without aneurysm formation was defined according to Daugherty’s modified classification^32^. The incidence of AA was determined as follows: mouse number with aortic aneurysm/group mice*100%. The maximum outer diameters of the aortic arch, thoracic aorta and suprarenal aorta were determined by Zeiss software (Carl Zeiss Far East Co., Ltd., Germany)^31^. For atherosclerosis assessment, the atherosclerotic plaque was quantified by measuring the surface area of the Oil Red O-positive lesions on *en face* preparations of aortic arches. The saline-perfused upper half of the heart, including the aortic root, was directly embedded in an optimal cutting temperature compound (O.C.T., Sakura Finetek, Co. Ltd., Tokyo, Japan), frozen in liquid nitrogen, and cryosectioned (10 μm). The ratio of atherosclerotic plaque area to total cross cryosection area of the aortic root was examined by Oil Red O staining (10 μm) and quantified by colorimetric analysis using Image J^33,34^.

### Serum levels determination of inflammatory cytokines, chemokines, bone formation markers, immune factors, biochemical and haematological parameters, and sclerostin

The serum of mice/rats was collected after treatment. ELISA kits for IL-6 (BMS603-2), TNF-α (BMS607-3), MCP-1 (BMS6005)^9^ from ThermoFisher, PINP (LS-F23905) from LifeSpan, sclersotin (DSST00) from R&D Systems were used. Serum levels of immune factors (TNF-α, IL-1β, IL-10, IL-12, IL-18 and IL-4) were determined using a tailored MILLIPLEX MAP rat cytokine kit (Millipore, Billerica, MA, USA)^35^. Biochemical parameters (ALT, AST and BUN) and haematological parameters (RBCs, haemoglobin, WBCs and PLTs) were analysed using a clinical chemistry analyser (Cruinn Diagnostics, Ltd., Dublin, Ireland) and an Auto Hematology Analyzer (Mindray International, Ltd., Shenzhen, China), respectively^13^.

### Immunohistochemistry (IHC) analysis

Paraffin cross-sections (5 μm) from suprarenal aortas and cross cryosections (10 μm) from aortic roots were obtained for immunohistochemistry analysis. Deparaffinized sections were rehydrated, boiled to retrieve antigens (10 mM citrate buffer, pH 6) and blocked with 5% BSA, while cryosections were directly blocked with 5% BSA. Suprarenal aorta and aortic root sections were incubated with rabbit anti-CD68 antibody (Abcam, ab125212, 1 μg/ml), rabbit anti-α-SMA antibody (Abcam, ab5694, 1:200), and rabbit anti-cleaved caspase-3 antibody (Abcam, ab2302, 10 μg/ml), followed by incubation with goat anti-rabbit IgG (Abcam, ab205718, 1:1000). The colour reaction was then developed by adding 3,3ʹ-diaminobenzidine (DAB). Positive staining areas were quantified by colorimetric analysis using ImageJ^36^.

### Micro-CT analysis

Micro-CT (version 6.5, vivaCT40, SCANCO Medical AG, Bassersdorf, Switzerland) was employed for analysis of the bone mass and bone architecture. For mice, the trabecular bone at the right proximal tibia metaphysis and the fourth lumbar vertebrae (Lv4) were analysed. For rats, the trabecular bone at the right distal femoral metaphysis, the right proximal tibia metaphysis and the fifth lumbar vertebrae, as well as the cortical bone at the right femoral mid-shaft, were analysed. Briefly, a total of 424 slices with a voxel size of 10 µm (for mice)/21 µm (for rats) were scanned at the region of the proximal tibia beginning at the growth plate and extending distally along the tibial diaphysis, the entire region of secondary spongiosa between proximal and distal aspects from the fourth (for mice)/fifth (for rats) vertebrae, and the region of femoral mid-shaft (for rats). Regions of interest (ROIs) were defined for trabecular (for both mice and rats) and cortical parameters (for rats) using Scanco evaluation software. Images of femurs, tibias and vertebrae were reconstructed and segmented (200 ms integration time, 0.8 sigma, 1 support, 260 thresholds). All measurements used the same filtering and segmentation values. For the proximal tibia and distal femur of mice, 100 sequential slices beginning at 0.1 mm from the most proximal aspect of the growth plate in which both condyles were no longer visible were selected for analysis. The trabeculae were analysed by manual contouring excluding the cortical bone. For distal femoral metaphysis and proximal tibia metaphysis of rats, the entire femora or tibia was reoriented with the mid-diaphysis parallel to the z-axis, and bone length was measured as the distance between the most proximal and distal transverse plans containing the femur. Beginning from the most proximal aspect of the growth plate, the trabeculae region on 100 consecutive slices at a distance of 1.4 mm away from the growth plate was selected. The trabeculae were analysed by manual contouring excluding the cortical bone. For the fourth (mice)/fifth (rats) lumbar vertebrae, a central region was selected equivalent to 70% of the vertebral body height, beginning at the distal growth plate and extending proximally along the vertebral body. The freehand trabeculae ROI on 100 sequential slices was drawn to ensure that it was within the endosteal envelope. Trabecular bone parameters, including trabecular volume per total volume (Tb.BV/TV), trabecular volumetric bone mineral density (Tb.vBMD), trabecular thickness (Tb.Th), trabecular number (Tb.N), trabecular spacing (Tb.Sp), trabecular structure model index (Tb.SMI) and trabecular connectivity density (Tb.conn.D) were calculated^37,38^. For the femoral mid-shaft of rats, 100 slices were measured at the exact centre and at the distal 50% of femur length using the automated thresholding algorithm. Trabeculae in contact with cortical bone were manually removed from the ROI. Cortical bone parameters, including cortical volumetric bone mineral density (Ct.vBMD) and cortical thickness (Ct. Th) were calculated^37,39–44^.

### Bone histomorphometric analysis

Calcein green (20 mg/kg) was injected intraperitoneally at 10 and 2 days (for mice) or 13 and 3 days (for rats) before euthanasia. After micro-CT analysis, the right proximal tibias (both mice and rats), the right distal femurs (rats) and the fifth lumbar vertebrae (rats), as well as the right femoral mid-shaft (rats), were dehydrated in increasing concentrations of sucrose (10%, 20%, 30% in PBS) for 24 h at each concentration and embedded without decalcification in an optimal cutting temperature compound (O.C.T., Sakura Finetek, Co., Ltd., Tokyo, Japan). Frontal sections (thickness: 7 μm) of trabecular bone were obtained from the proximal tibia (both mice and rats), distal femurs (rats) and fifth lumbar vertebrae (rats) by longitudinal cryosection with CryoStar NX50 (Thermo Fisher Scientific, Waltham, MA, USA). Cross sections (thickness: 7 μm) of cortical bone (rats) were obtained from the femoral mid-shaft with CryoStar NX50. Fluorescence micrographs of the bone sections with calcein green labels were captured by a Q500MC fluorescence microscope (Leica, Bensheim, Germany). The parameters of bone dynamic histomorphometric analysis, including the bone formation rate (BFR/BS) and mineral apposition rate (MAR), were analysed using a histomorphometric analysis system (BIOQUANT OSTEO, Nashville, TN, USA) and calculated and expressed according to the ASBMR standardized nomenclature for bone histomorphometry^37,42^.

### Bone mechanical test

The fourth lumbar vertebrae (Lv4) and the left femurs of the rats were directly stored at −80 °C after sacrifice. The compression test and three-point test were performed by a universal testing machine (H25KS Series, Hounsfield Test Equipment Ltd, Redhill, UK) with a 2.5 kN load cell^45^. For the compression test, Lv4s were isolated from vertebral columns and constructed into a cylinder with two parallel planes (5–7 cm) before the test. The Lv4s were positioned horizontally to the base. Load was applied constantly with a displacement rate of 1 mm/min. After failure, the load vs. displacement curves were recorded, and the failure force (N) and ultimate strength (MPa) were calculated for statistical analysis. For the three-point test, femurs were loaded in the anterior-posterior direction with the span set as 17 mm. Load was applied with a constant displacement rate of 1 mm/min at the femur mid-shaft. After failure, the load *vs*. displacement curves were recorded, and the failure force (N), stiffness and fracture energy (J) were calculated for statistical analysis.

### Random library, primers and buffers for SELEX

The ssDNA library used in SELEX contained a 40-nucleotide (nt) randomized region and an 18 nt conserved region at each end for primer annealing: 5ʹ- CGTACGGTCGACGCTAGC-(N)40-CACGTGGAGCTCGGATCC-3ʹ. FP 5ʹ-CGTACGGTCGACGCTAGC-3ʹ and biotinylated RP 5ʹ-biotin-GGATCCGAGCTCCACGTG-3ʹ were used for the amplification of ssDNA during selection. All oligos were synthesized and purified by HPLC^46–49^. Binding and washing buffers were prepared with phosphate buffered saline (PBS) pH 7.4 with 1 mM MgCl_2_, 5 mM imidazole and 0.05% Tween 20.

### Protein SELEX

Recombinant His_6_-human sclerostin was immobilized on NTA magnetic beads at 4 °C for 1 h. The ssDNA library was denatured at 95 °C for 10 min and rapidly cooled to 0 °C for 10 min followed by incubation with immobilized sclerostin at room temperature (RT) for 0.5–1 h. The total incubation volume was 1 ml. BSA (10 µg in the 1st round and 1 µg in the following rounds) was included to avoid nonspecific binding. Unbound sequences were removed with washing buffer. After washing, the bound sequence-protein beads were collected and subjected to PCR amplification with FP and Bio-RP (step 1: 95 °C for 1 min for initial denaturation; step 2: 12 cycles of 95 °C for 30 s, 56 °C for 30 s, and 72 °C for 30 s; and step 3: 72 °C for 2 min). PCR products were applied to streptavidin magnetic beads through biotin-streptavidin binding according to the manufacturer’s instructions. Single-stranded sequences were regenerated with 0.1 M NaOH^49^. Negative selection was performed against recombinant loop3-deficient sclerostin, blank NTA beads and other His_6_-tagged proteins. After 20 rounds of selection, the identified sequences were sent for next-generation sequencing by Illumina MiSeq (PCT No: PCT/CN2019/074764, PCT Pub No.: WO2019/15440).

### Enzyme-Linked OligoNucleotide Assay (ELONA)

Target protein (160 ng) was coated into a 96-well microtiter plate in 100 µl of PBS and incubated at 4 °C overnight. The plate was then blocked with blocking buffer (PBS, 0.1% Tween 20 and 1% BSA) for 1 h at RT and washed with washing buffer (PBS, 1 mM MgCl_2_, 0.1% Tween 20 and 0.1% BSA) 4 times. The aptamer candidates were denatured at 95 °C for 10 min and rapidly cooled on ice for 10 min before use. Appropriate concentrations of biotinylated aptamers were added to each well (100 µl) and incubated for 45 min at RT with continuous gentle shaking. For cells, adherent cells were used directly for binding to aptamers. After binding, the plate was washed with SELEX B&W buffer 4 times. Then, 100 µl of streptavidin-HRP (1:10000 dilute into PBST + 0.1% BSA) was added to each well and incubated for 30 min and washed with PBST + 0.1% BSA 4 times. Then, 50 µl of TMB was added to each well and incubated for 20 min. The reaction was stopped by adding 50 µl of 2 M H_2_SO_4_. The absorbance at 450 nm was measured with a microplate reader^50^. Data were analysed with Origin (OriginLab, Northampton, MA, USA). A nonlinear curve fitting model Hyperbl was used to plot the binding curve. The equation of the Hyperbl model is y = P1*x/(P2 + x), and P2 is the Kd value.

### Pharmacokinetic analysis of the modified aptamer after administration in vivo

After a single subcutaneous (S.C.) administration of aptscl56 or Apc001PE in rats, blood samples (~200 μl) were collected at different time points (aptscl56: 5 min, 15 min, 30 min, 1 h, 2 h, 4 h, 8 h, 12 h, 24 h; Apc001PE: 5 min, 15 min, 30 min, 1 h, 2 h, 4 h, 8 h, 12 h, 24 h, 30 h, 36 h, 48 h, 54 h, 62 h, 70 h, 76 h, 84 h, 96 h, 107 h, *n* = 6 in each group) via the orbital vein^51,52^. The plasma was isolated within 1 h and stored at −80 °C^51,53,54^. Prior to analysis, plasma samples were incubated with proteinase K solution (1 mg/ml proteinase K in 10 mM Tris HCl, pH 7.5, 20 mM CaCl_2_, 10% glycerol v/v) in digestion buffer (60 mM Tris-HCl, pH 8.0, 100 mM EDTA and 0.5% SDS) at 55 °C overnight with shaking. The samples were then centrifuged (16,900 g, 4 °C, 15 min), and the supernatant was taken for analysis^54^.

The HPLC system was equipped with a C4 column to quantify Apc001PE in plasma samples, while a C18 column was utilized for the quantification of aptscl56. Both methods used a mobile phase elution gradient made from phase A (TEAA, pH 7.0) and phase B (acetonitrile). The column oven temperature was 50 °C. Standards were prepared in blank rat plasma containing sodium heparin with different concentrations of aptscl56/Apc001PE^53^. All reported concentrations of aptscl56 and Apc001PE were based on the mass of aptscl56. The aptscl56 and Apc001PE concentrations versus time profile were plotted and analysed by DAS 3.0 (BioGuider Co., Shanghai, China)^55,56^.

The dosing interval for Apc001PE administration could be defined based on the elimination half-life (T_1/2_) and the dosing ratio of the loading dosage to the maintenance dosage of Apc001PE^55,56^. In the following in vivo studies, as the loading dosage and maintenance dosage of Apc001PE were set to the same dose (dosing ratio <2), the dosing interval was twice weekly, which was slightly longer than T_1/2_ (66.9 h).

### Statistical analysis

All variables are expressed as the mean ± standard deviation. All statistical data were analysed by GraphPad Prism (version 8; GraphPad Software, Inc., San Diego, CA, USA), and *P* < 0.05 was considered statistically significant. For the in vivo experiments, the sample size was predetermined by a power calculation and our previously published paper^44^. The animals were grouped randomly and blinded to the researchers. Animals in poor body condition were excluded.

### Reporting summary

Further information on research design is available in the Nature Research Reporting Summary linked to this article.

## Supplementary information

Supplementary Information

Reporting Summary

## Data Availability

The authors declare that the data supporting the findings of this study are available within the paper and its supplementary information files. Source data are provided with this paper.

## Source data

Source Data
