# Supplementary material for: Targeting loop3 of sclerostin preserves its cardiovascular protective action and promotes bone formation
Source: Nat Commun. 2022 Jul 22;13:4241. doi: 10.1038/s41467-022-31997-8 (PMC9307627; doi:10.1038/s41467-022-31997-8)
Supplement: Supplementary file 1 — Supplementary Information [file 41467_2022_31997_MOESM1_ESM.pdf]

Targeting loop3 of sclerostin preserves its cardiovascular protective action and promotes bone formation

Yuanyuan Yu[1,2,3,4#*], Luyao Wang[1,2,3,4#], Shuaijian Ni[1,2,3,4#], Dijie Li[1,2,3,4], Jin Liu[1,2,3,4], Hang Yin Chu[2,5], Ning Zhang[2,5], Meiheng Sun[1,2,3,4], Nanxi Li[1,2,3,4], Qing Ren[1,2,3,4], Zhenjian Zhuo[2,5], Chuanxin Zhong[1,2,3,4,6], Duoli Xie[1,2,3,4], Yongshu Li[1,2,3,4], Zong-Kang Zhang[2,5], Huarui Zhang[1,2,3,4], Mei Li[7], Zhenlin Zhang[8], Lin Chen[9], Xiaohua Pan[10], Weibo Xia[7], Shu Zhang[11], Aiping Lu[1,2,3,4*], Bao-Ting Zhang[2,5*] & Ge Zhang[1,2,3,4*]

[1] Law Sau Fai Institute for Advancing Translational Medicine in Bone and Joint Diseases (TMBJ), School of Chinese Medicine, Hong Kong Baptist University, Hong Kong SAR, China

[2] Guangdong-Hong Kong-Macao Greater Bay Area International Research Platform for Aptamer-based Translational Medicine and Drug Discovery (HKAP), Hong Kong SAR, China

[3] Institute of Precision Medicine and Innovative Drug Discovery (PMID), School of Chinese Medicine, Hong Kong Baptist University, Hong Kong SAR, China

[4] Institute of Integrated Bioinformedicine and Translational Science (IBTS), School of Chinese Medicine, Hong Kong Baptist University, Hong Kong SAR, China

[5] School of Chinese Medicine, Faculty of Medicine, The Chinese University of Hong Kong, Hong Kong SAR, China

[6] Department of Materials Science and Engineering, Southern University of Science and Technology, Shenzhen, China

[7] Department of Endocrinology, National Health Commission Key Laboratory of Endocrinology, Peking Union Medical College Hospital, Chinese Academy of Medical Sciences and Peking Union Medical College, Beijing, China

[8] Shanghai Clinical Research Center of Bone Disease, Department of Osteoporosis and Bone Disease, Shanghai Jiao Tong University Affiliated Sixth People's Hospital, Shanghai, China

[9] Department of Wound Repair and Rehabilitation Medicine, State Key Laboratory of Trauma, Burns and Combined Injury, Trauma Center, Research Institute of Surgery, Daping Hospital, Army Medical University, Chongqing, China

[10] Orthopedic Center, Shenzhen University Affiliated Second Hospital (Shenzhen Baoan People's Hospital), Shenzhen, China

[11] The Key Laboratory of Aerospace Medicine, Ministry of Education, Air Force Medical University, Xi'an, Shaanxi, China,

[#] These authors contributed equally: Yuanyuan Yu, Luyao Wang and Shuaijian Ni.

* These authors are corresponding authors who jointly supervised this work: Yuanyuan Yu (email: yuyuanyuan@hkbu.edu.hk); Aiping Lu (email: aipinglu@hkbu.edu.hk); Bao-Ting Zhang (email: zhangbaoting@cuhk.edu.hk) and Ge Zhang (email: zhangge@hkbu.edu.hk).

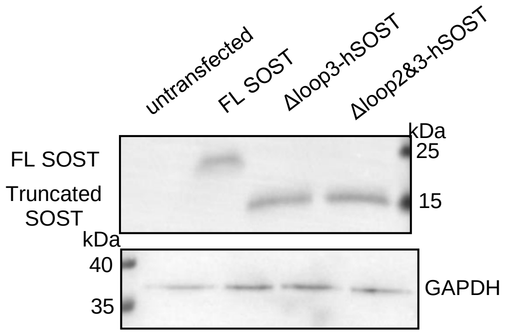

Supplementary Fig. 1 The expression levels of full length sclerostin and sclerostin truncations were determined after transfected into MC3T3-E1 cells. FL hSOST: Full length human sclerostin, MW: 22.3 kDa; Δloop3-hSOST: sclerostin with loop3 deficiency by genetic truncation (1-110), MW: 13 kDa and Δloop2&3-hSOST: sclerostin with loop2&3 deficiency by genetic truncation (1-85), MW: 10.5 kDa. All plasmids were constructed with a sequence encoding His6-tag at the C-terminal of the target gene. Anti-6x His tag primary antibody was used for detecting sclerostin. Each experiment was repeated independently for three times with similar results.

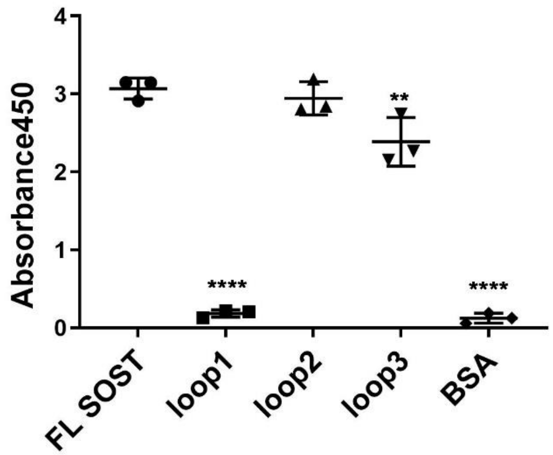

Supplementary Fig. 2 Binding ability of sclerostin antibody to full length sclerostin (FL SOST), sclerostin loop1, loop2 and loop3, respectively. **Note:** FL SOST: full length sclerostin (1-189), loop1 (57-80), loop2 (86-109) and loop3 (111-140). $p<0.0001$ (loop1), $p=0.0035$ (loop3), $p<0.0001$ (BSA). Data were expressed as mean ± standard deviation. One-way ANOVA with Tukey's post-hoc test *vs* FL SOST was used to determine the inter-group differences, respectively. n = 3 per group. **** $p < 0.0001$. **Note**: BSA: Bovine Serum Albumin. Source data are provided as a Source Data file.

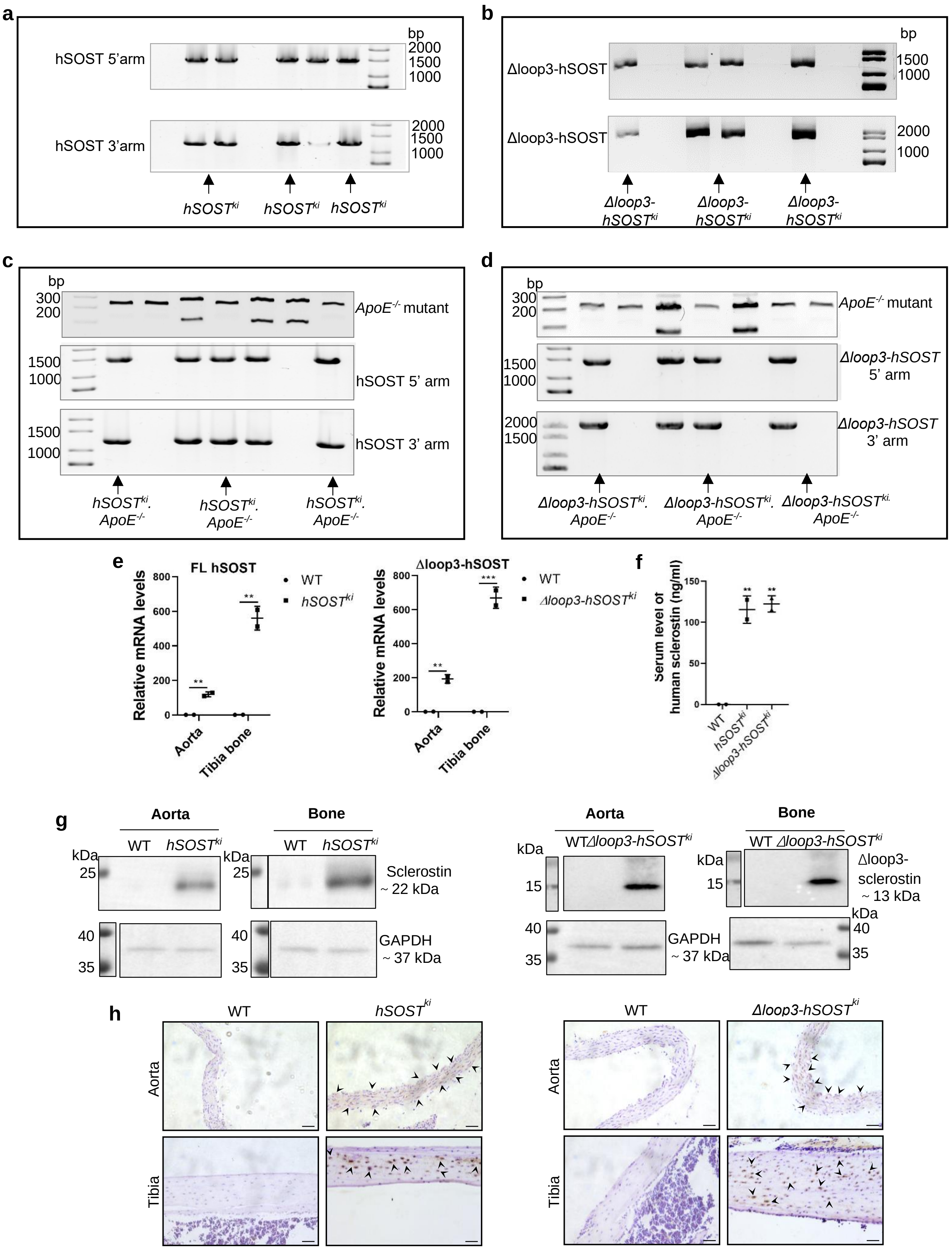
a
bp
2000
1500
1000
hSOST 5'arm
hSOST 3'arm
hSOST^ki
b
Δloop3-hSOST
Δloop3-hSOST^ki
c
300
200
ApoE^-/- mutant
hSOST 5' arm
hSOST 3' arm
hSOST^ki. ApoE^-/-
d
Δloop3-hSOST 5' arm
Δloop3-hSOST 3' arm
Δloop3-hSOST^ki. ApoE^-/-
e
FL hSOST
Δloop3-hSOST
Relative mRNA levels
WT
hSOST^ki
Δloop3-hSOST^ki
Aorta
Tibia bone
f
Serum level of human sclerostin (ng/ml)
g
Aorta
Bone
kDa
Sclerostin ~ 22 kDa
GAPDH ~ 37 kDa
Δloop3-sclerostin ~ 13 kDa
h
WT
Aorta
Tibia

Supplementary Fig. 3 Genotyping and phenotyping for mice models. a Representative agarose gel electrophoretic images for genotyping of *hSOST*^{ki} mice. **b** Representative agarose gel electrophoretic images for genotyping of *Δloop3-hSOST*^{ki}mice. **c** Representative agarose gel electrophoretic images for genotyping of *hSOST*^{ki}*.ApoE*$^{-/-}$ mice. **d** Representative agarose gel electrophoretic images for genotyping of *Δloop3-hSOST*^{ki}*.ApoE*$^{-/-}$ mice. *ApoE*$^{-/-}$ mutant: ~ 245 bp (*homozygous*); *hSOST*^{ki}: 5'arm ~ 1465 bp, 3'arm ~ 1229 bp; *Δloop3-hSOST*^{ki}: 5'arm ~ 1465 bp, 3'arm ~ 2149 bp. **e** qPCR for determination of the mRNA expression levels of FL SOST and Δloop3-hSOST in the aorta and bone of *hSOST*^{ki} and *Δloop3-hSOST*^{ki} mice, respectively. *hSOST*^{ki}: p=0.0069 (aorta), p=0.0074 (tibia bone); *Δloop3-hSOST*^{ki}: p=0.0081 (aorta), p=0.0043 (tibia bone). Data were expressed as the mean ± standard deviation. n=2 per group. ** $p < 0.01$ and *** $p < 0.005$ for a comparison between two groups with a paired *t* test. **f** ELISA for determination of the protein expression levels of FL SOST and Δloop3-hSOST in the serum of *hSOST*^{ki} and *Δloop3-hSOST*^{ki} mice, respectively. p=0.0033 (WT *vs. hSOST*^{ki}), p=0.0028 (WT *vs. Δloop3-hSOST*^{ki}). Data were expressed as the mean ± standard deviation. n=2 per group. ** $p < 0.01$ for a comparison *vs*. WT by one-way ANOVA with Tukey's post-hoc test. **g** Western blot for determining the expression levels of FL SOST and Δloop3-hSOST in the aorta and bone of *hSOST*^{ki} and *Δloop3-hSOST*^{ki} mice, respectively. **h** IHC for determination of the expression levels of FL SOST and Δloop3-hSOST in the aorta and bone of *hSOST*^{ki} and *Δloop3-hSOST*^{ki} mice, respectively. Scale bars:50 µm. For **a-d, g** and **h**, each experiment was repeated independently for three times with similar results. Source data are provided as a Source Data file.

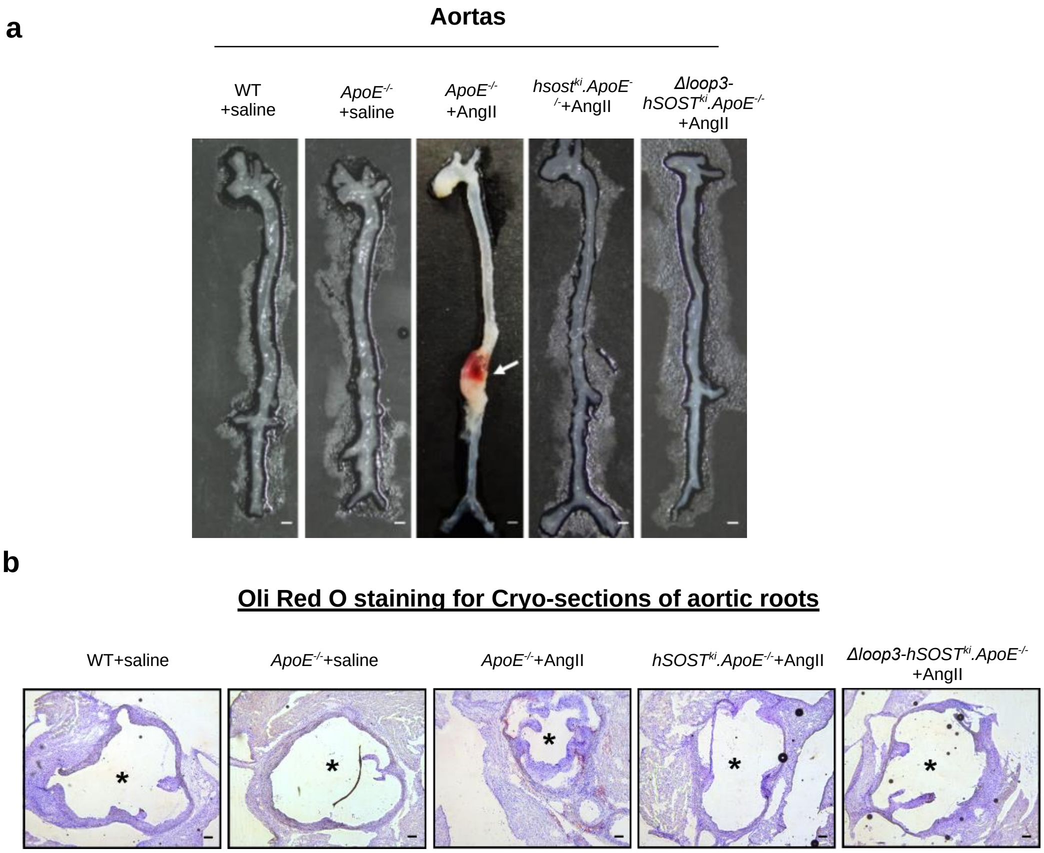

Supplementary Fig. 4 Determination of whether loop3 deficient sclerostin by genetic truncation could maintain the protective effect of sclerostin on cardiovascular system in *ApoE$^{-/-}$* mice with loop3 deficient human sclerostin knock-in (*Δloop3-SOST^{ki}.ApoE$^{-/-}$*) mice. Comparisons were performed among *ApoE$^{-/-}$* mice, *ApoE$^{-/-}$* mice with full-length human sclerostin knock-in (*hSOST^{ki}.ApoE$^{-/-}$*) and *ApoE$^{-/-}$* mice with loop3 deficient human sclerostin knock-in (*Δloop3-SOST^{ki}.ApoE$^{-/-}$*) and *ApoE$^{-/-}$* mice with AngII infusion. **a** Representative image of aortas from each group of mice. The white arrows indicated locations of aortic aneurysm (AA). Scale bars, 1 mm. **b** Representative micrographs of aortic roots stained with Oil Red O. Scale bar, 100 μm (*lumen). For a and b, each experiment was repeated independently for three times with similar results.

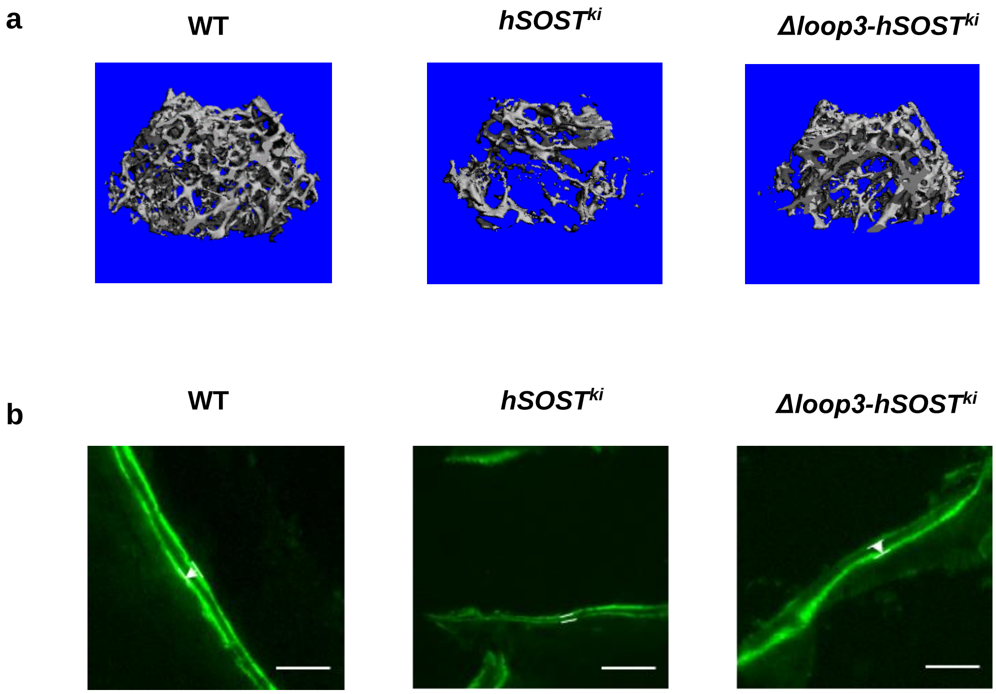

Supplementary Fig. 5 Determination of whether loop3 deficiency by genetic truncation could attenuate the inhibitory effect of sclerostin on bone formation in *Δloop3-hSOST*^{ki}, compared to *hSOST*^{ki} and WT mice, respectively. (a) Representative images showing the three-dimensional trabecular architecture by micro-CT reconstruction at the fourth vertebrae. Scale bars, 200 µm. **(b)** Representative fluorescent micrographs of the trabecular bone sections showing bone formation at the fourth vertebrae visualized by double calcein labels. Arrows indicated the spaces between the double calcein labeling. Scale bars, 50 µm. For **a** and **b**, each experiment was repeated independently for three times with similar results.

a

Item Name	Sequence
Loop3	IPDRYRAQRVQLLCPGGEAPRARKVRLVAS
Loop3-1	AAARYRAQRVQLLCPGGEAPRARKVRLVAS
Loop3-2	IPDAAAAQRVQLLCPGGEAPRARKVRLVAS
Loop3-3	IPDRYRAAAAQLLCPGGEAPRARKVRLVAS
Loop3-4	IPDRYRAQRVAAACPGGEAPRARKVRLVAS
Loop3-5	IPDRYRAQRVQLLAAAGEAPRARKVRLVAS
Loop3-6	IPDRYRAQRVQLLCPGAAAPRARKVRLVAS
Loop3-7	IPDRYRAQRVQLLCPGGEAAAARKVRLVAS
Loop3-8	IPDRYRAQRVQLLCPGGEAPRAAAARLVAS
Loop3-9	IPDRYRAQRVQLLCPGGEAPRARKVAAAAS
Loop3-10	IPDRYRAQRVQLLCPGGEAPRARKVRLVAA
Loop3m	IPDAAAAAAAQLLCPGAAAPRAAAARLVAS

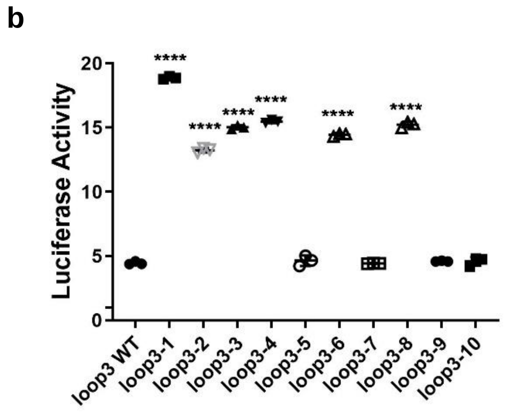

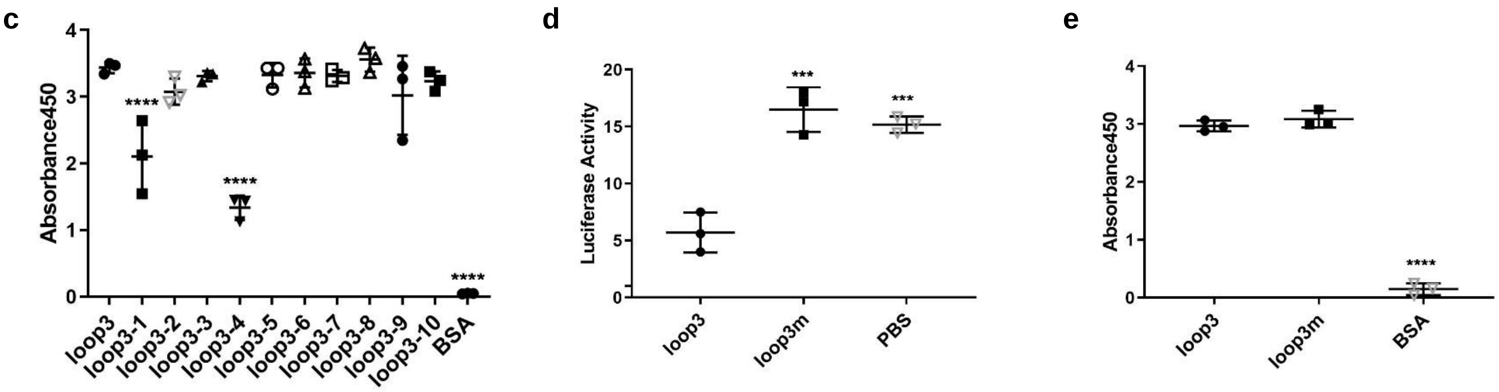

Supplementary Fig. 6 Determination of the functional sites on sclerostin loop3 participated in antagonizing Wnt signaling and in interacting with aptscl56. (a) The sequences of WT sclerostin loop3 and loop3 mutants. The mutation sites were shown in red font and underlined. **(b)** The effects of WT loop3/loop3 mutants on regulating Wnt signaling in MC3T3-E1 cells). Compared to that in MC3T3-E1 cells treated with WT loop3, the luciferase signals in MC3T3-E1 cells treated with mutants loop3-1 (I111A, P112A, D113A), loop3-2 (R114A, Y115A, R116A), loop3-3 (Q118A, R119A, V120A), loop3-4 (Q121A, L122A, L123A), loop3-6 (G127A, E128A) and loop3-8 (R133A, K134A, V135A) were significantly higher, indicating that residues I111, P112, D113, R114, Y115, R116, Q118, R119, V120, Q121, L122, L123, G127,E128, R133, K134 and V135 could be the functional sites participated in antagonizing Wnt signaling. $p<0.0001$ (loop3-1, loop3-2, loop3-3, loop3-4, loop3-6, loop3-8). **(c)** The binding abilities of sclerostin loop3 mutants to aptscl56. Compared to WT loop3, the binding abilities of mutants loop3-1 (I111A, P112A, D113A), and loop3-4 (Q121A, L122A, L123A) to aptscl56 were significantly lower, suggesting that residues I111, P112, D113, Q121, L122 and L123 could be the interaction sites on sclerostin loop3 to aptscl56. $p<0.0001$ (loop3-1, loop3-4, BSA). **(d)** The effects of sclerostin loop3m (R114A, Y115A, R116A, Q118A, R119A, V120A, G127A, R133A, K134A, V135A) on regulating Wnt signaling in MC3T3-E1 cells. Compared to that in MC3T1-E1 cells treated with WT loop3, the luciferase signal in MC3T3-E1 cells treated with loop3m was significantly higher, indicating that loop3m could not antagonize Wnt signaling in MC3T3-E1 cells. $p=0.0003$ (loop3m), $p=0.0006$ (PBS). (**e**) The binding ability of loop3m to aptscl56 were determined. There were no significant differences for the binding ability to aptscl56 between the WT loop3 and loop3m, demonstrating that loop3m could still bind to aptscl56. $p<0.0001$ (BSA). For **b** to **e**, data were expressed as the mean ± standard deviation. n=3 per group. *** $p < 0.005$; **** $p < 0.0001$ for one-way ANOVA with Tukey's post-hoc test *vs.* wild type (WT) loop3 was used to determine the inter-group differences. Source data are provided as a Source Data file.

a

Name	Sequence (5'-3')	Length (nt)
aptscl56	CGGGGTGTGGGTTCGTCGTTAGCTTGATTTGGCAGCTGCC	40
aptscl56-1	AAAGGTGTGGGTTCGTCGTTAGCTTGATTTGGCAGCTGCC	40
aptscl56-2	CGGAAAGTGGGTTCGTCGTTAGCTTGATTTGGCAGCTGCC	40
aptscl56-3	CGGGGTAAAGGTTCGTCGTTAGCTTGATTTGGCAGCTGCC	40
aptscl56-4	CGGGGTGTGAAATCGTCGTTAGCTTGATTTGGCAGCTGCC	40
aptscl56-5	CGGGGTGTGGGTAAATCGTTAGCTTGATTTGGCAGCTGCC	40
aptscl56-6	CGGGGTGTGGGTTCGAAATTAGCTTGATTTGGCAGCTGCC	40
aptscl56-7	CGGGGTGTGGGTTCGTCGAAAACTTGATTTGGCAGCTGCC	40
aptscl56-8	CGGGGTGTGGGTTCGTCGTTAGAAAGATTTGGCAGCTGCC	40
aptscl56-9	CGGGGTGTGGGTTCGTCGTTAGCTTAAAATGGCAGCTGCC	40
aptscl56-10	CGGGGTGTGGGTTCGTCGTTAGCTTGATTAAACAGCTGCC	40
aptscl56-11	CGGGGTGTGGGTTCGTCGTTAGCTTGATTTGGAAAATGCC	40
aptscl56-12	CGGGGTGTGGGTTCGTCGTTAGCTTGATTTGGCAGCAAAC	40
aptscl56-13	CGGGGTGTGGGTTCGTCGTTAGCTTGATTTGGCAGCTGCA	40

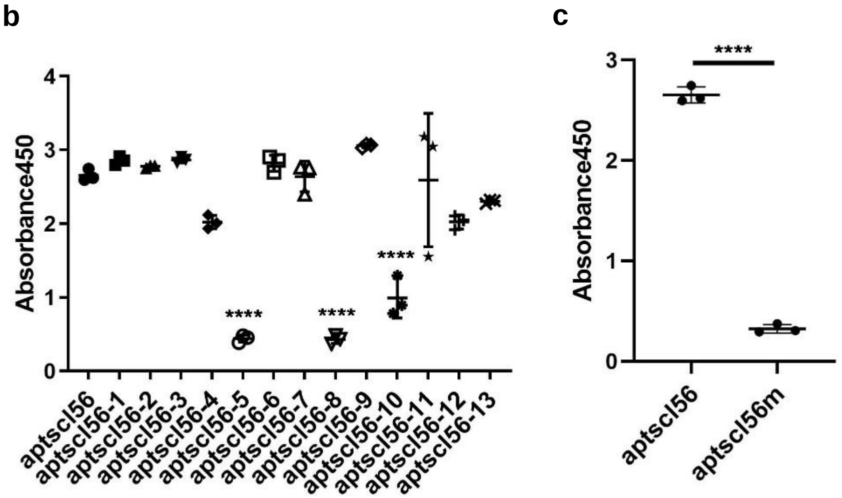

Supplementary Fig. 7 Binding abilities of the aptscl56 mutants to sclerostin. (a) The sequences of WT aptscl56 and aptscl56 mutants. The mutation sites were shown in red font and underlined. **(b)** The binding abilities of WT/aptscl56 mutants to sclerostin were determined. Data were expressed as mean ± standard deviation. One-way ANOVA with Tukey's post-hoc test *vs* WT aptscl56 was used to determine the inter-group differences, respectively). Compared to WT aptscl56, the binding abilities of mutants aptscl56-5 (T13A, C14A, G15A), aptscl56-8 (C23A, T24A, T25A), and aptscl56-10 (T30 A, G31A, G32A) to sclerostin were significantly lower, whereas the binding abilities of other aptamer mutants to sclerostin were not altered, suggesting that nucleotides T13C14G15, C23T24T25 and T30G31G32 could be the interaction sites of aptscl56 to sclerostin. p<0.0001 (aptscl56-5, aptscl56-8, aptscl56-10). Data were expressed as the mean ± standard deviation. n=3 per group. **** $p < 0.0001$ for one-way ANOVA with Tukey's post-hoc test *vs.* aptscl56 was used to determine the inter-group differences. **(c)** An aptscl56 mutant (aptscl56m) with simultaneous mutations (T13A, C14A, G15A, C23A, T24A, T25A, T30 A, G31A and G32A) were synthesized and the binding ability of aptscl56m to sclerostin was determined. Compared to WT aptscl56, the binding ability of aptscl56m was significantly lower, indicating that aptacl56m could not bind to sclerostin. p<0.0001. Data were expressed as mean ± standard deviation. n=3 per group. A two tailed unpaired t test was used to determine the differences between two groups. **** $p < 0.0 001$. Source data are provided as a Source Data file.

a

Item Name	Sequence
Loop 2	GPARLLPNAIGRGKWWRPSGPDFR
Loop 2-1	AAARLLPNAIGRGKWWRPSGPDFR
Loop 2-2	GPAAAAPNAIGRGKWWRPSGPDFR
Loop 2-3	GPARLLAAAIGRGKWWRPSGPDFR
Loop 2-4	GPARLLPNAAAAGKWWRPSGPDFR
Loop 2-5	GPARLLPNAIGRAAAWRPSGPDFR
Loop 2-6	GPARLLPNAIGRGKWAAASGPDFR
Loop 2-7	GPARLLPNAIGRGKWWRPAAADFR
Loop 2-8	GPARLLPNAIGRGKWWRPSGPAAA

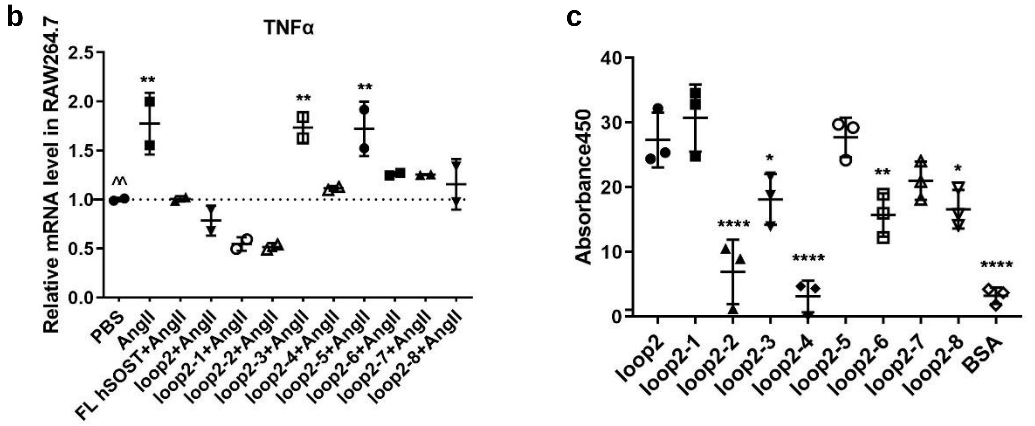

Supplementary Fig. 8 Determination of the functional sites on sclerostin loop2 participated in suppressing the expression of inflammatory cytokine and in interacting with therapeutic sclerostin antibody. (a) The sequences of WT sclerostin loop2 and loop2 mutants. The mutation sites were shown in red font and underlined. **(b)** The effects of WT sclerostin/loop2 mutants on suppressing the mRNA level of TNFα in RAW264.7 macrophages with AngII treatment *in vitro*. Compared to RAW 264.7 treated with WT loop2, mRNA level of TNFα were significantly higher in RAW 264.7 treated with mutants loop2-3 (P92A, N93A) and loop2-5 (G98A, K99A, W100A), indicating that residues P92, N93, G98, K99, W100 could be the functional sites on loop2 which participated in sclerostin's suppressive effect on expression of inflammatory cytokine. p=0.0740 (PBS *vs.* AngII), p=0.0030 (AngII), p=0.0047 (loop2-3+AngII), p=0.0054 (loop2-5+AngII). **(c)** The binding abilities of sclerostin loop2 mutants to therapeutic sclerostin antibody. Compared to WT loop2, the binding abilities of mutants loop2-2 (R89A, L90A, L91A), loop2-3 (P92A, N93A), loop2-4 (I95A, G96A, R97A), loop2-6 (W101A, R102A, P103A), loop2-8 (D107A, F108A, R109A) to therapeutic antibody were significantly lower, suggesting that residues R89, L90, L91, P92, N93, I95, G96, R97, W101, R102, P103, D107, F108, R109 could be the interaction sites on sclerostin loop2 to therapeutic antibody. Loop2-5 (G98A, K99A and W100A) which could not suppress the expression of inflammatory cytokine, but not affect the binding to therapeutic sclerostin antibody was name loop2m in the following studies. p<0.0001 (loop2-2, loop2-4, BSA), p=0.0352 (loop2-3), p=0.0059 (loop2-6), p=0.0117 (loop2-8). For **b** and **c**, data were expressed as the mean ± standard deviation. n=3 per group. ^^$p < 0.01$ for a comparison between PBS and AngII group by a two tailed unpaired *t*-test. * $p < 0.05$; ** $p < 0.01$; *** $p < 0.005$ and **** $p < 0.0001$ for one-way ANOVA with Tukey's post-hoc test *vs.* loop2 was used to determine the inter-group differences. Source data are provided as a Source Data file.

a 5’-C$_m$G$_m$G$_m$G$_m$GTGTGGGTTCGTCGTTAGCTTGATTTGGCAGCTG$_m$C$_m$C$_m$C$_m$-idT-3’

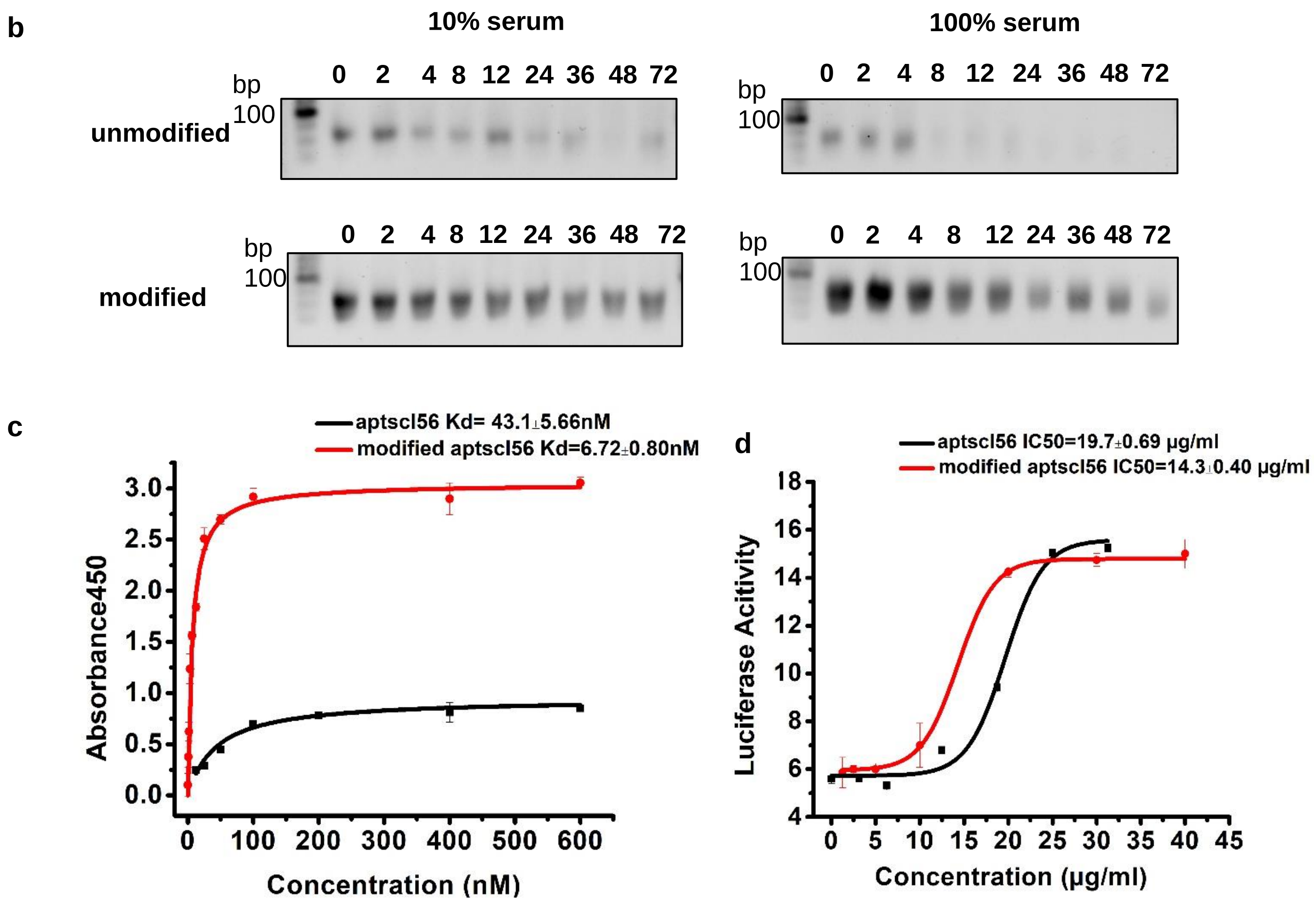

Supplementary Fig. 9 Chemical modifications on aptscl56 and characterizations of the modified aptscl56. (a) The chemical modification strategies for aptscl56. **(b)** The serum stability assay of the modified aptscl56 compared to the unmodified aptscl56. Each experiment was repeated independently for three times with similar results. **(c)** The binding affinity of the unmodified and modified aptscl56 against sclerostin, respectively. **(d)** The inhibitory potency of the unmodified and modified aptscl56 against sclerostin’s antagonistic effect on Wnt signaling, respectively. For **c** and **d**, data were expressed as the mean ± standard deviation. n=3 per group. Source data are provided as a Source Data file.

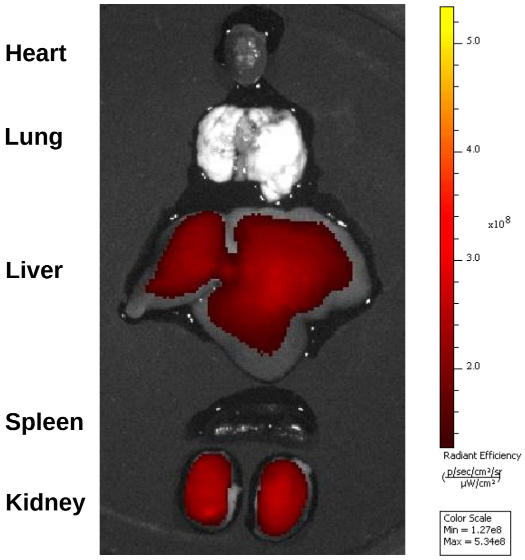

Supplementary Fig. 10 The distribution of PEG40k-aptscl56 (Apc001PE) in major viscera (heart, liver, spleen, lung, and kidney) at organ level 3 days after one single subcutaneous injection (25 mg/kg) of Apc001PE in mice visualized by biophotonic imaging. Experiment was repeated independently for three times with similar results. Source data are provided as a Source Data file.

a

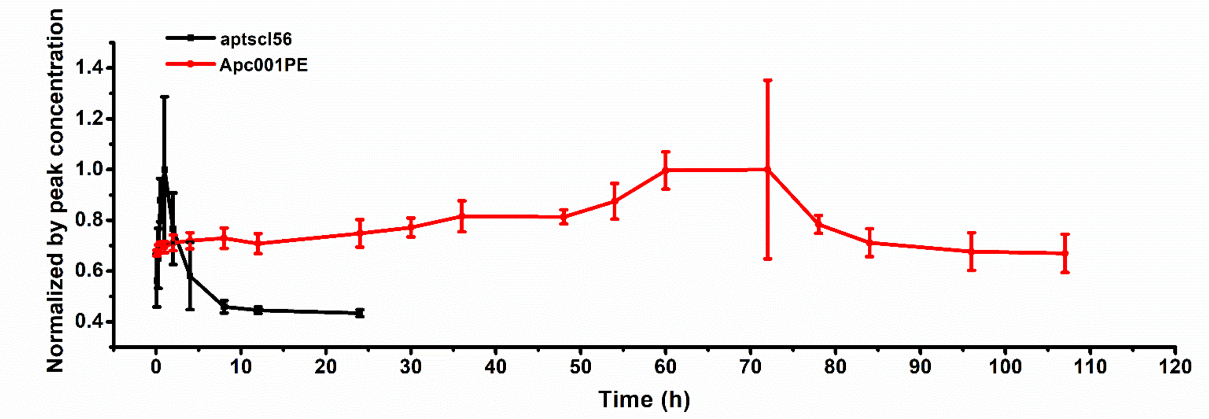

b

Parameter	Mean value		Unit
	aptscl56	Apc001PE	
T_{max}	0.5	72	h
AUC	1205	11802	(μg h)/ml
CL/F	0.004	0.001	L/h/kg
Elim $T_{1/2}$	1.8	66.9	h

Supplementary Fig. 11 Pharmacokinetic analysis of aptscl56 and PEG40k-aptscl56 (Apc001PE) after a single subcutaneous administration in rats. (a) The rat plasma concentrations for aptscl56 and Apc001PE were normalized by the corresponding peak concentration, respectively. Data were expressed as the mean ± standard deviation. n=3 per group. (b) Pharmacokinetic parameters of aptscl56 and PEG40k-aptscl56 (Apc001PE) calculated from the rat plasma concentrations. Source data are provided as a Source Data file.

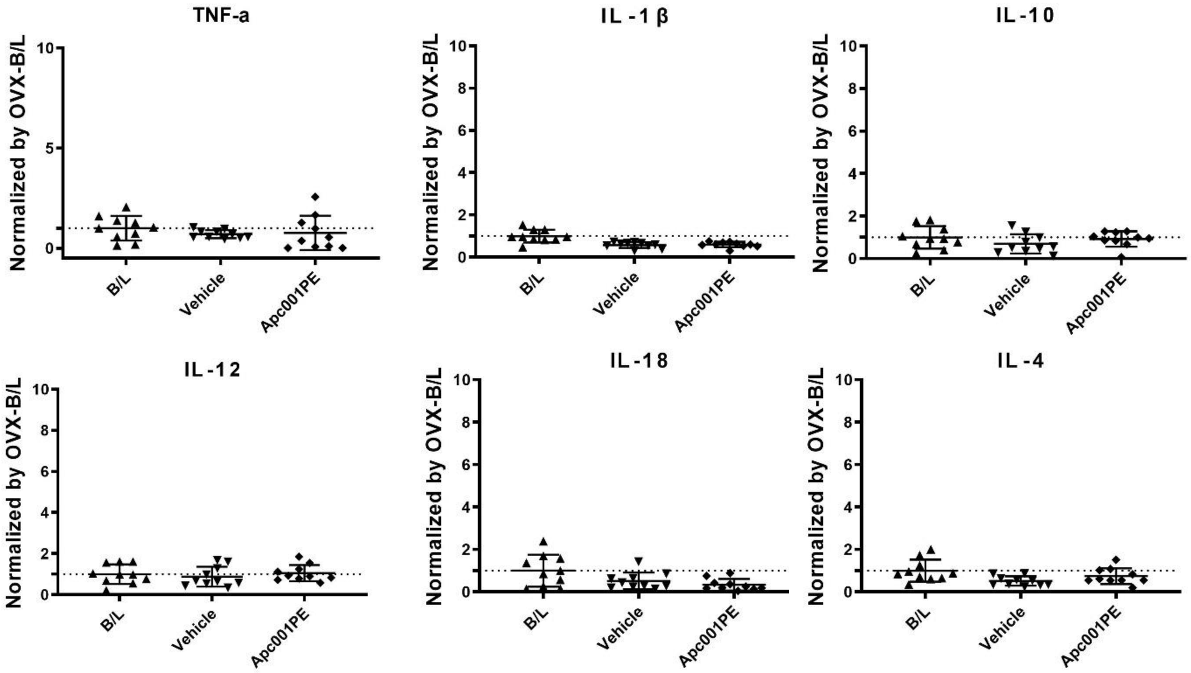

Supplementary Fig. 12 Serum levels of immune factors in healthy SD rats after 6-week administration of Apc001PE (12 mg/kg, twice/week). Serum levels of TNF-α, IL-1β, IL-10, IL-12, IL-18 and IL-4 were determined by ELISA and normalized to vehicle group, respectively. **Note:** TNF-α: tumor necrosis factor-α; IL-1β: interleukin-1β; IL-10: interleukin-10; IL-12: interleukin-12; IL-18: interleukin-18; IL-4: interleukin-4. Data were expressed as mean ± standard deviation by one-way ANOVA with Tukey's post-hoc test *vs* B/L, n = 10 per group. Source data are provided as a Source Data file.

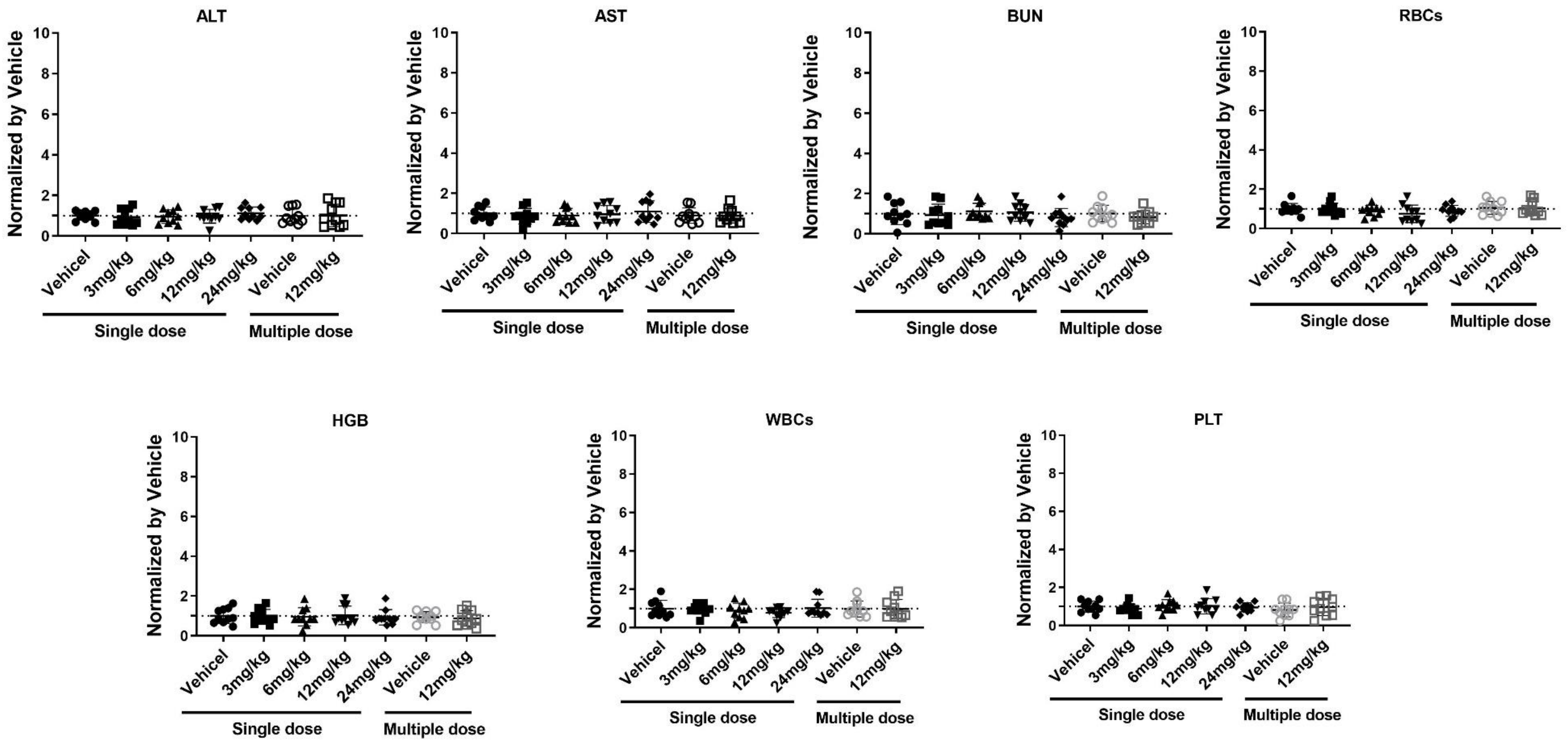

Supplementary Fig. 13 Biochemistry and hematology assays for toxicity evaluation in healthy SD rats after a single or multiple administration(s) of Apc001PE. ALT: alanine aminotransferase; AST: aspartate aminotransferase; BUN: blood urea nitrogen; RBCs: red blood cells; HGB: haemoglobin; WBCs: white blood cells; PLTs: platelets. Data were expressed as mean ± standard deviation followed by one-way ANOVA with Tukey's post-hoc test *vs* Vehicle, n = 10 per group. Source data are provided as a Source Data file.

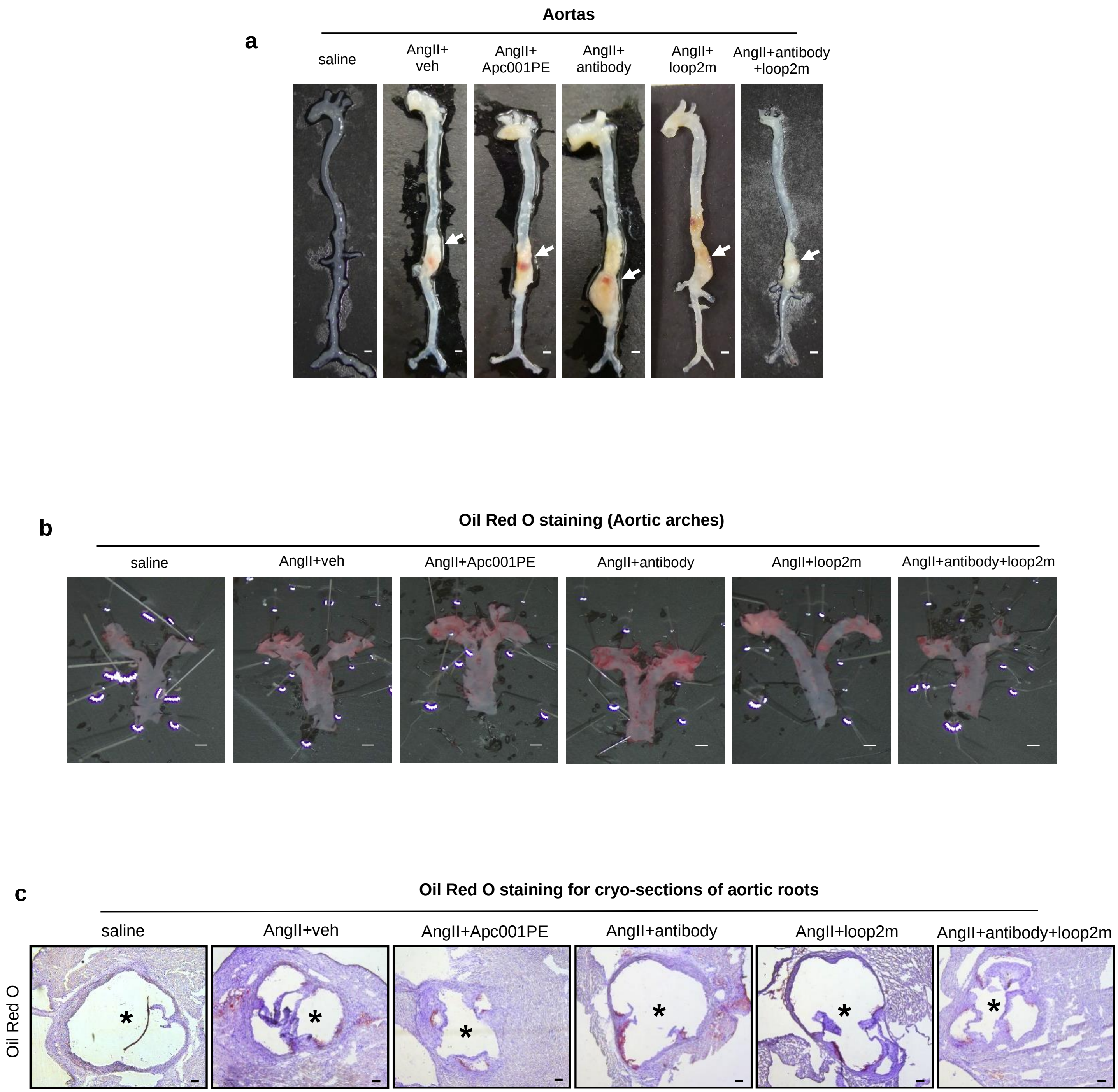
a
Aortas
saline
AngII+
veh
AngII+
Apc001PE
AngII+
antibody
AngII+
loop2m
AngII+antibody
+loop2m
b
Oil Red O staining (Aortic arches)
saline
AngII+veh
AngII+Apc001PE
AngII+antibody
AngII+loop2m
AngII+antibody+loop2m
c
Oil Red O staining for cryo-sections of aortic roots
saline
AngII+veh
AngII+Apc001PE
AngII+antibody
AngII+loop2m
AngII+antibody+loop2m
Oil Red O

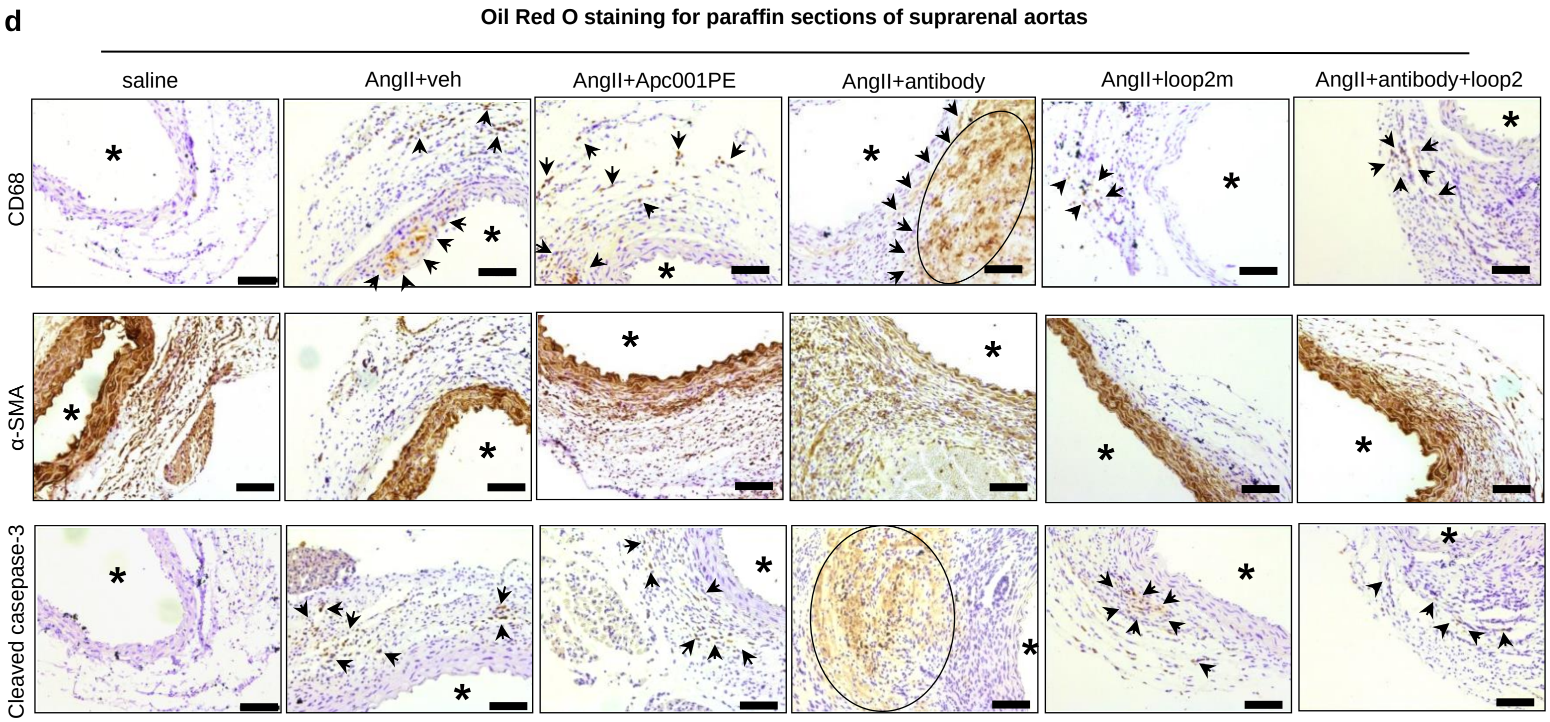
d
Oil Red O staining for paraffin sections of suprarenal aortas
saline
AngII+veh
AngII+Apc001PE
AngII+antibody
AngII+loop2m
AngII+antibody+loop2
CD68
α-SMA
Cleaved casepase-3

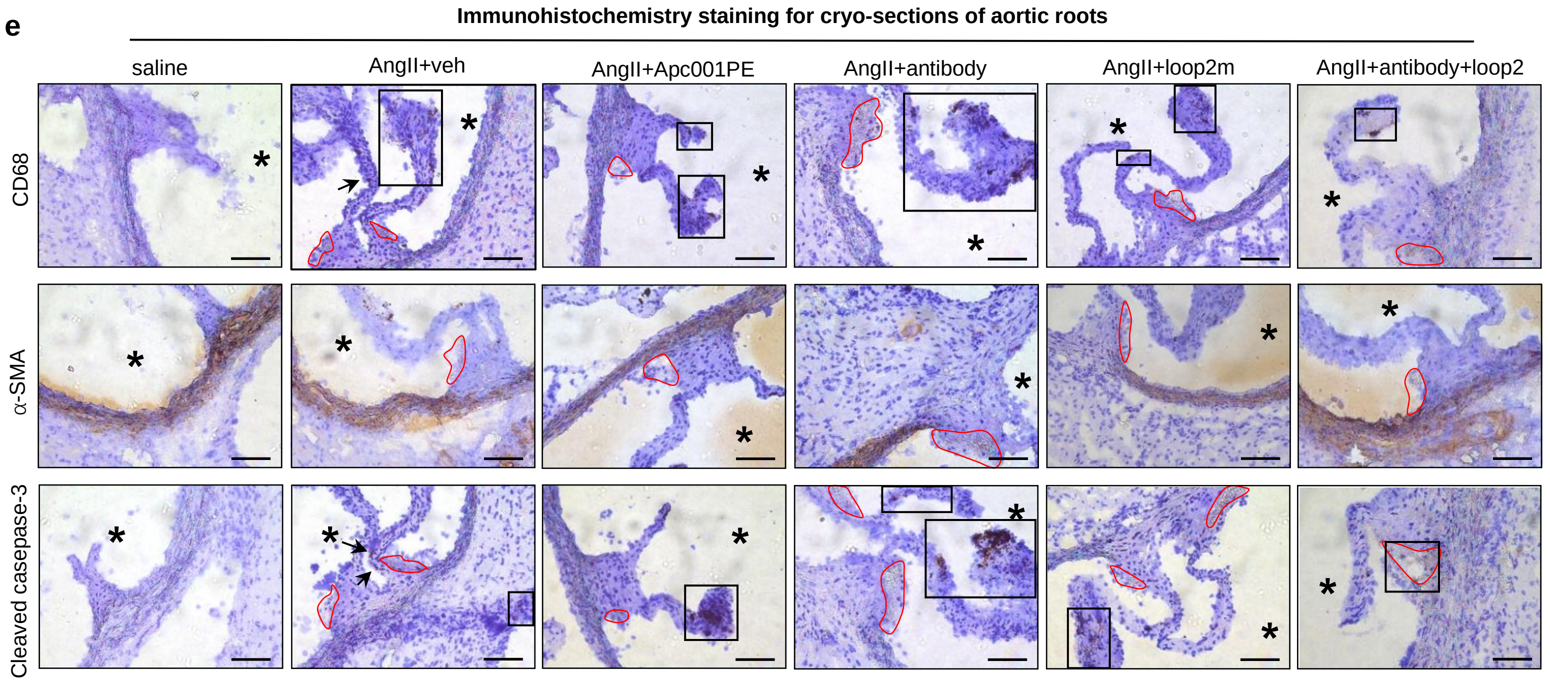
e
Immunohistochemistry staining for cryo-sections of aortic roots
saline
AngII+veh
AngII+Apc001PE
AngII+antibody
AngII+loop2m
AngII+antibody+loop2
CD68
α-SMA
Cleaved casepase-3

Supplementary Fig. 14 Evaluation of whether targeting sclerostin loop3 by the specific *in vivo* pharmacologic tool Apc001PE had effect on the cardiovascular events in *ApoE$^{-/-}$* mice with AngII infusion. *ApoE$^{-/-}$* mice with AngII infusion were subcutaneously administrated with vehicle, Apc001PE (12 mg/kg), humanized therapeutic sclerostin antibody (25 mg/kg), fatty acid-loop2m (6 mg/kg), and humanized therapeutic sclerostin antibody pretreated with fatty acid-loop2m (6 mg/kg), respectively, for four weeks. After administration, the aortas were harvested for analysis. **(a)** Representative images of aortas from each group. The white arrows indicated the locations of aortic aneurysm (AA). Scale bars, 1 mm. **(b)** Oil Red O staining of aortic arch for quantifying atherosclerosis in *ApoE$^{-/-}$* mice. Scale bars, 1 mm. **(c)** Representative micrographs of cryo-sections of aortic roots from *ApoE$^{-/-}$* mice stained with Oil Red O. Scale bars, 100 μm (*lumen). **(d)** Representative immunohistochemistry images for the expression of CD68, α-SMA and cleaved caspase-3 in suprarenal aortas (the black arrows and black circles indicated the locations of positive staining). Scale bar: 100 μm (*lumen). **(e)** Representative immunohistochemistry images for the expression of CD68, α-SMA and cleaved caspase-3 in aortic roots (the red circles indicated the locations of atherosclerotic plaque, the black arrows and black squares indicated the locations of positive staining. Scale bar: 100 μm (*lumen). For **a** to **e**, each experiment was repeated independently for three times with similar results. **Note**: AngII: Angiotensin II; IL-6: interleukin 6; MCP-1: monocyte chemoattractant protein-1; TNF-α: tumor necrosis factor alpha; CD68: macrophages biomarker; α-SMA: contractile cell biomarker; Cleaved caspase-3: apoptotic cell biomarker; loop2m: the loop2 mutant (GPARLLPNAIGRAAAWRPSGPDFR).

.

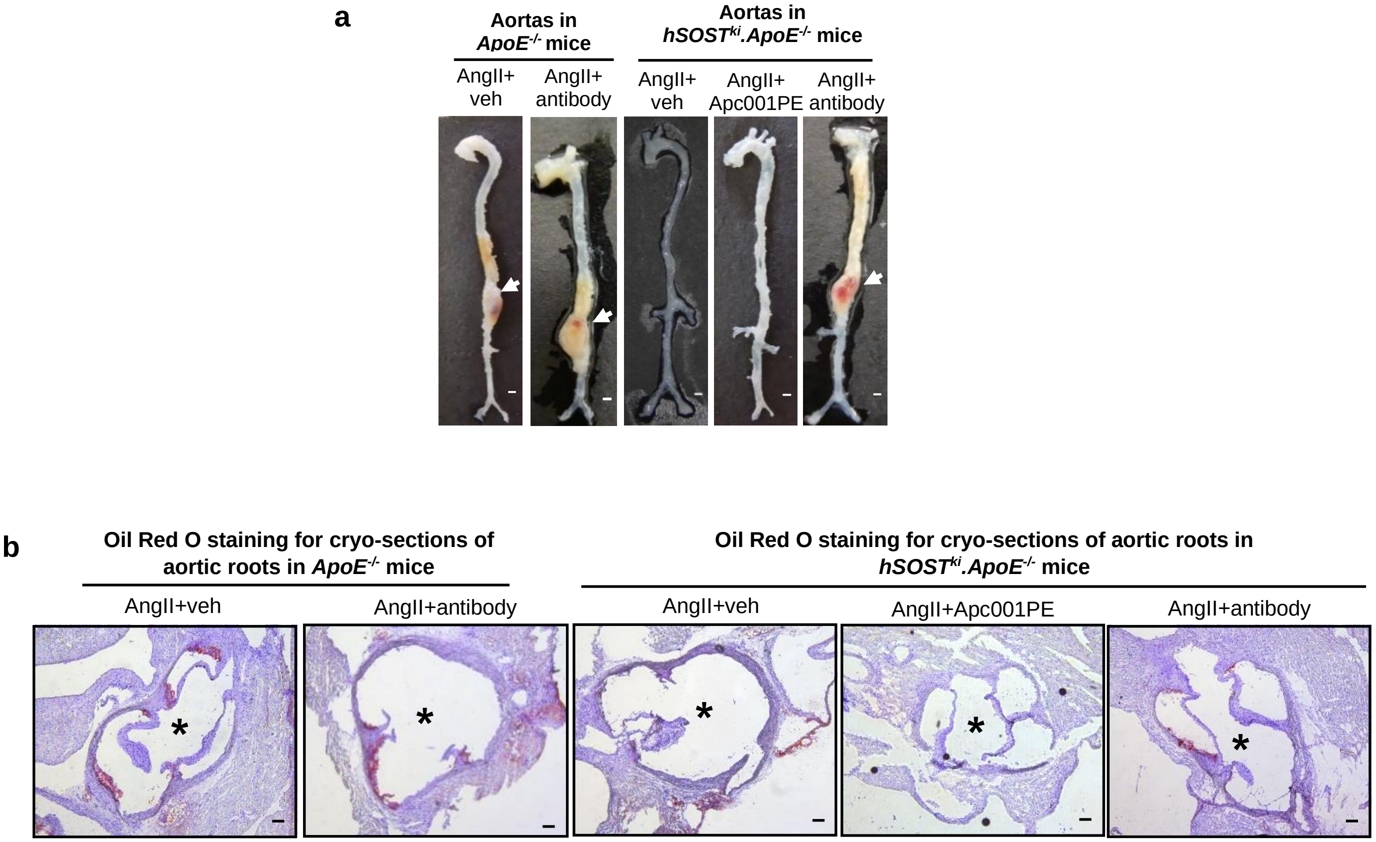

Supplementary Fig. 15 Determination of whether targeting sclerostin loop3 by the specific *in vivo* pharmacologic tool Apc001PE had influence on the protective effect of the overexpressed human sclerostin on cardiovascular system by evaluating cardiovascular events in *hSOST^{ki}.ApoE$^{-/-}$* mice with AngII infusion. *ApoE$^{-/-}$* or *hSOST^{ki}.ApoE$^{-/-}$* mice with AngII infusion were subcutaneously administrated with vehicle, Apc001PE (12 mg/kg) and humanized therapeutic sclerostin antibody (25 mg/kg), respectively. After administration, the aortas were harvested for analysis. **(a)** Representative images of aortas from each group. The white arrows indicated the locations of aortic aneurysm (AA). Scale bars, 1 mm. **(b)** Representative microphages of cryo-sections of aortic roots stained with Oil Red O. Scale bar, 100 μm (*lumen). For **a** and **b**, each experiment was repeated independently for three times with similar results. **Note**: AngII: Angiotensin II; IL-6: interleukin 6; MCP-1: monocyte chemoattractant protein-1; TNF-α: tumor necrosis factor alpha.

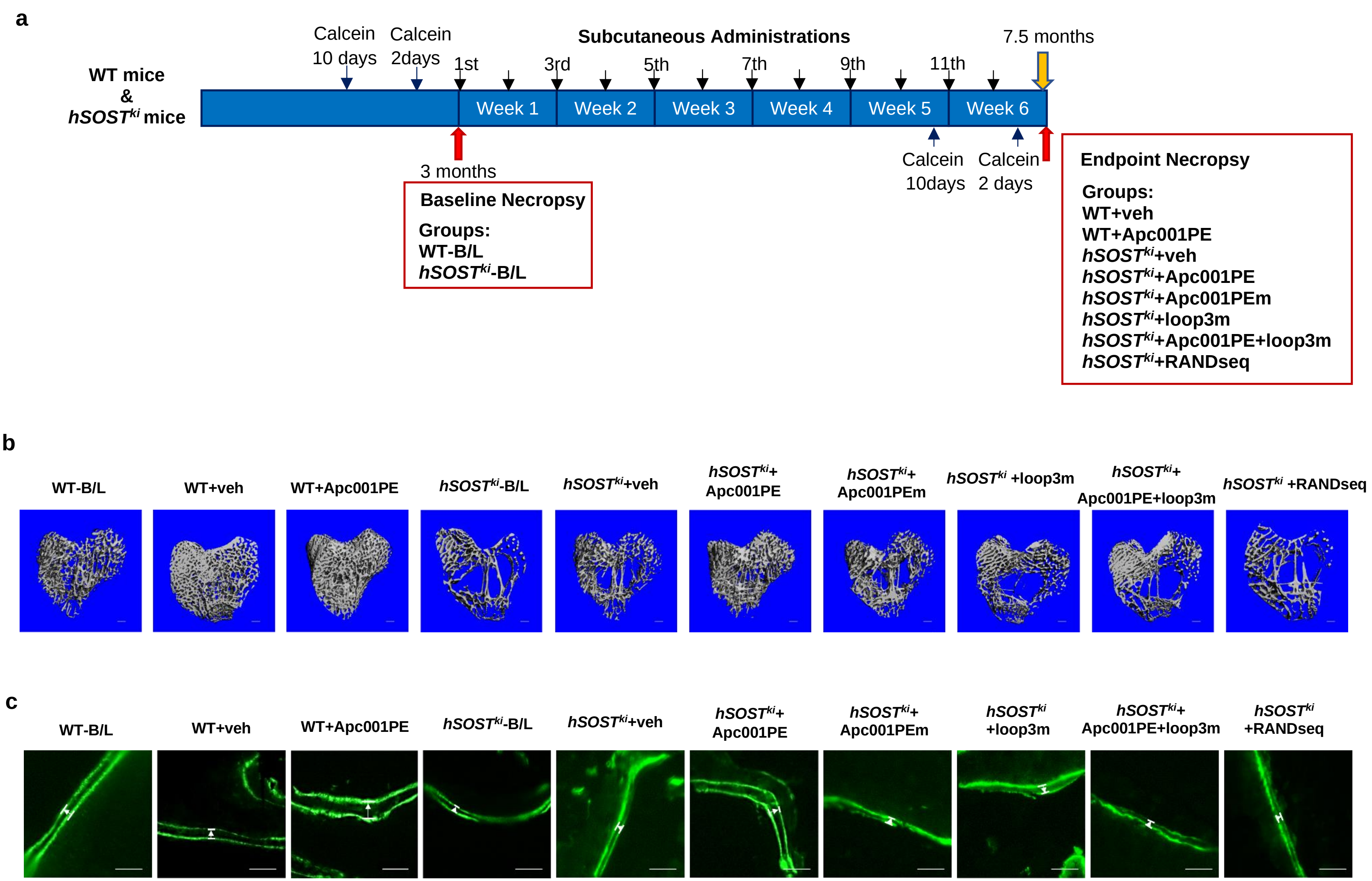
a
WT mice
&
hSOSTki mice
Calcein
10 days
Calcein
2days
Subcutaneous Administrations
1st
3rd
5th
7th
9th
11th
7.5 months
Week 1
Week 2
Week 3
Week 4
Week 5
Week 6
3 months
Baseline Necropsy
Groups:
WT-B/L
hSOSTki-B/L
Calcein
10days
Calcein
2 days
Endpoint Necropsy
Groups:
WT+veh
WT+Apc001PE
hSOSTki+veh
hSOSTki+Apc001PE
hSOSTki+Apc001PEm
hSOSTki+loop3m
hSOSTki+Apc001PE+loop3m
hSOSTki+RANDseq
b
WT-B/L
WT+veh
WT+Apc001PE
hSOSTki-B/L
hSOSTki+veh
hSOSTki+ Apc001PE
hSOSTki+ Apc001PEm
hSOSTki +loop3m
hSOSTki+ Apc001PE+loop3m
hSOSTki +RANDseq
c
WT-B/L
WT+veh
WT+Apc001PE
hSOSTki-B/L
hSOSTki+veh
hSOSTki+ Apc001PE
hSOSTki+ Apc001PEm
hSOSTki +loop3m
hSOSTki+ Apc001PE+loop3m
hSOSTki +RANDseq

Supplementary Fig. 16 Evaluation of whether targeting sclerostin loop3 by the specific *in vivo* pharmacologic tool Apc001PE could attenuate the inhibitory effect of sclerostin on bone formation in *hSOST*^{ki} mice. (a) A schematic diagram showing the experiment design of the study. Before treatment, ten 3-month-old wide-type C57BL/6J mice and ten 3-month-old *hSOST*^{ki} mice were euthanized as baseline (WT-B/L and *hSOST*^{ki}-B/L). The rest WT or *hSOST*^{ki} mice were subcutaneously injected twice weekly with vehicle (veh), Apc001PE (12 mg/kg), fatty acid-loop3m (6 mg/kg), Apc001PE+fatty acid-loop3m (12 mg/kg+ 6 mg/kg) and PEG40k-random DNA sequence (RANDseq, 12 mg/kg), respectively, for 6 weeks (n = 10 for each group). Before euthanasia, all the animals were injected intraperitoneally with calcein green (20 mg/kg) at 10 and 2 days, respectively. After euthanasia, the right proximal tibia was performed with micro-CT and histomorphometric analysis. **(b)** Representative images showing three-dimensional trabecular architecture by micro-CT reconstruction at the proximal tibia. Scale bars, 200 µm. **(c)** The representative fluorescent micrographs of the trabecular bone sections showing bone formation at the proximal tibia visualized by double calcein labels. Arrows indicated the spaces between the double calcein labeling. Scale bars, 50 µm. For **a** to **c**, each experiment was repeated independently for three times with similar results. **Note**: Apc001PEm: PEGylated aptscl56 mutant with T13A, C14A, G15A, C23A, T24A, T25A, T30A, G31A and G32A mutations.

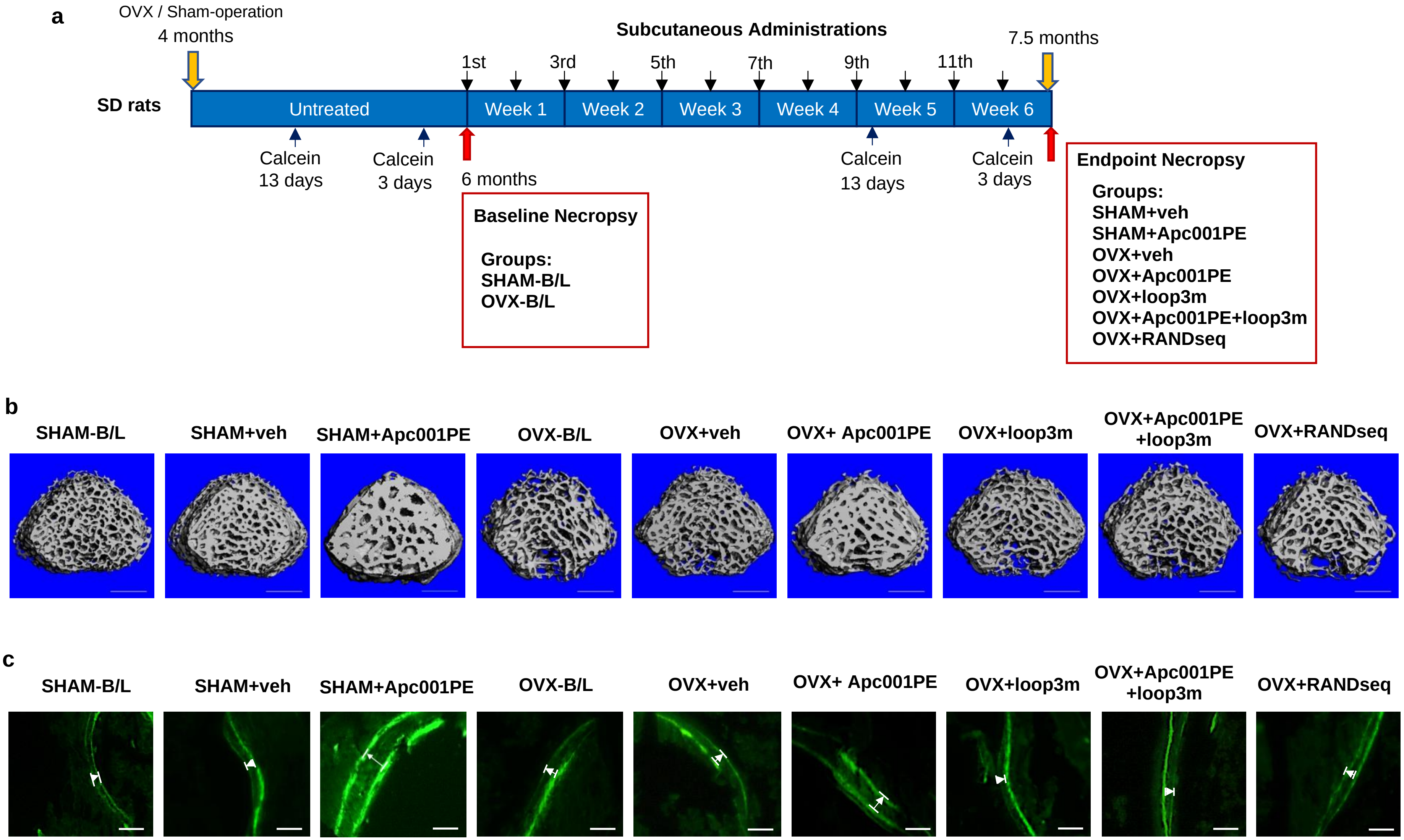
a
OVX / Sham-operation
4 months
Subcutaneous Administrations
7.5 months
1st
3rd
5th
7th
9th
11th
SD rats
Untreated
Week 1
Week 2
Week 3
Week 4
Week 5
Week 6
Calcein
13 days
Calcein
3 days
6 months
Calcein
13 days
Calcein
3 days
Baseline Necropsy
Groups:
SHAM-B/L
OVX-B/L
Endpoint Necropsy
Groups:
SHAM+veh
SHAM+Apc001PE
OVX+veh
OVX+Apc001PE
OVX+loop3m
OVX+Apc001PE+loop3m
OVX+RANDseq
b
SHAM-B/L
SHAM+veh
SHAM+Apc001PE
OVX-B/L
OVX+veh
OVX+ Apc001PE
OVX+loop3m
OVX+Apc001PE +loop3m
OVX+RANDseq
c
SHAM-B/L
SHAM+veh
SHAM+Apc001PE
OVX-B/L
OVX+veh
OVX+ Apc001PE
OVX+loop3m
OVX+Apc001PE +loop3m
OVX+RANDseq

Supplementary Fig. 17 Determination of whether targeting sclerostin loop3 by the specific *in vivo* pharmacologic tool Apc001PE could exert the bone anabolic potential via neutralizing the circulating sclerostin loop3 in ovariectomy-induced (OVX) osteoporotic rats. a A schematic diagram showing the experiment design of the study. Eighty four-month-old female Sprague Dawley rats were ovariectomized (OVX, n = 60) or sham-operated (SHAM, n = 20) and left untreated for 2 months. Ten OVX rats and ten SHAM-operated rats were euthanized before the treatment as baseline (OVX-B/L and SHAM- B/L). The rest SHAM or OVX rats were injected subcutaneously twice weekly with vehicle (veh), Apc001PE (12 mg/kg), fatty acid-loop3m (6 mg/kg), Apc001PE+fatty acid-loop3m (12 mg/kg+ 6 mg/kg) and PEG40k-random DNA sequence (RANDseq, 12 mg/kg), respectively, for 6 weeks (n = 10 for each group). The administration dose of Apc001PE and fatty acid-loop3m referred to the amount of aptscl56 and loop3m, respectively. All the animals were euthanized at the 6th week after the first injection. Before euthanasia, all the animals were injected intraperitoneally with calcein green (20 mg/kg) at 13 and 3 days, respectively. After euthanasia, the fifth lumbar were performed with micro-CT and bone histomorphometric analysis. **b** Representative images showing three-dimensional trabecular architecture at the fifth vertebrae. **c** The representative fluorescent micrographs showing bone formation of the trabecular bone sections at the fifth vertebrae visualized by double calcein labels. Arrows indicated the spaces between the double calcein labeling. Scale bars, 40 µm. For **b** and **c**, each experiment was repeated independently for three times with similar results.

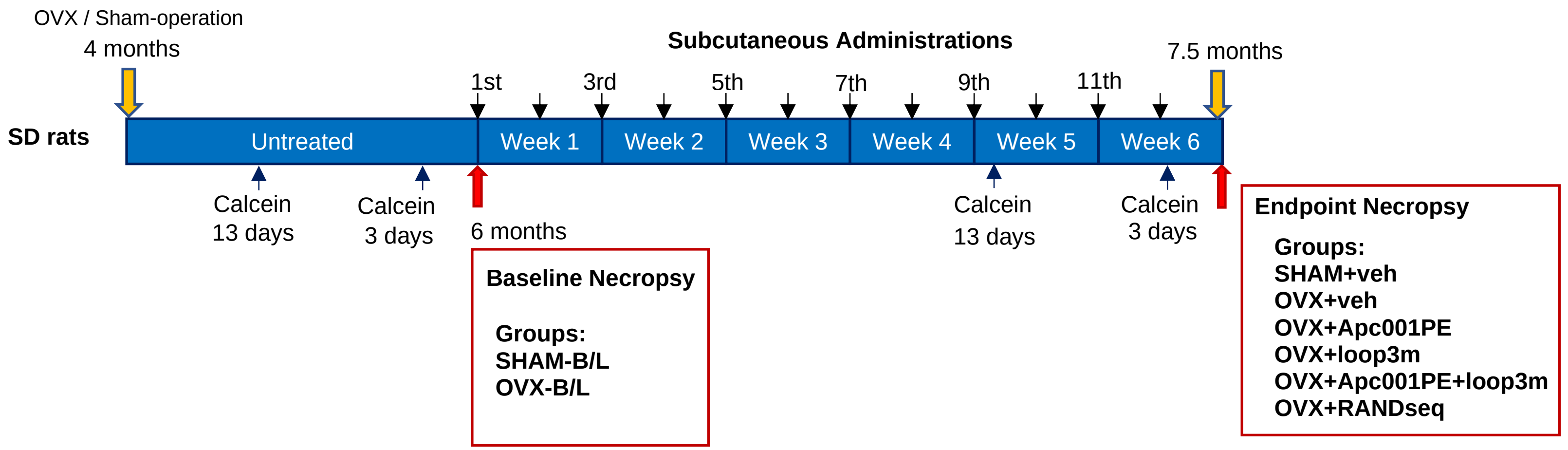

Supplementary Fig. 18 Determination of whether targeting sclerostin loop3 by the specific *in vivo* pharmacologic tool Apc001PE could exert the bone anabolic potential via neutralizing the circulating sclerostin loop3 in ovariectomy-induced (OVX) osteoporotic rats. a A schematic diagram showing the experiment design of the study. Eighty four-month-old female Sprague Dawley rats were ovariectomized or sham-operated and left untreated for 2 months. Ten OVX rats and ten SHAM-operated rats were euthanized before the treatment as baseline (OVX-B/L and SHAM- B/L). The rest SHAM or OVX rats were injected subcutaneously twice weekly with vehicle (veh), Apc001PE (12 mg/kg), fatty acid-loop3m (6 mg/kg), Apc001PE+fatty acid-loop3m (12 mg/kg+ 6 mg/kg) and PEG40k-random DNA sequence (RANDseq, 12 mg/kg), respectively, for 6 weeks (n = 10 for each group). The administration dose of Apc001PE and fatty acid-loop3m referred to the amount of aptscl56 and loop3m, respectively. Before euthanasia, all the animals were injected intraperitoneally with calcein green (20 mg/kg) at 13 and 3 days, respectively. After euthanasia, the right distal femoral metaphysis, the right proximal tibia metaphysis, the proximal tibia and the right femoral mid-shaft were performed with micro-CT and bone histomorphometric analysis. The fourth lumbar vertebrae and the left femora were used for mechanical test.

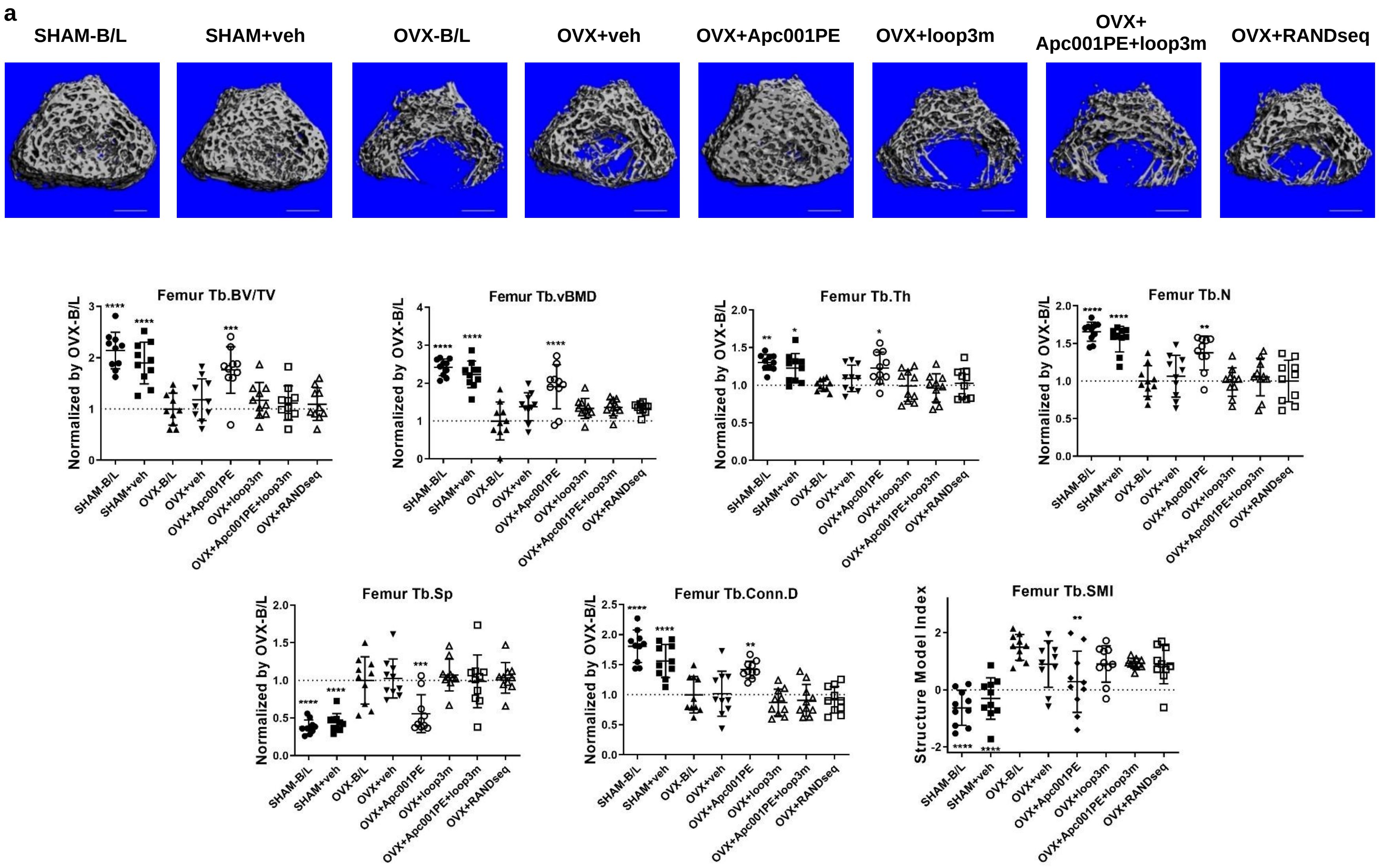
a
SHAM-B/L
SHAM+veh
OVX-B/L
OVX+veh
OVX+Apc001PE
OVX+loop3m
OVX+ Apc001PE+loop3m
OVX+RANDseq
Femur Tb.BV/TV
Femur Tb.vBMD
Femur Tb.Th
Femur Tb.N
Femur Tb.Sp
Femur Tb.Conn.D
Femur Tb.SMI
Normalized by OVX-B/L
Structure Model Index
OVX+Apc001PE+loop3m
OVX+RANDseq

b

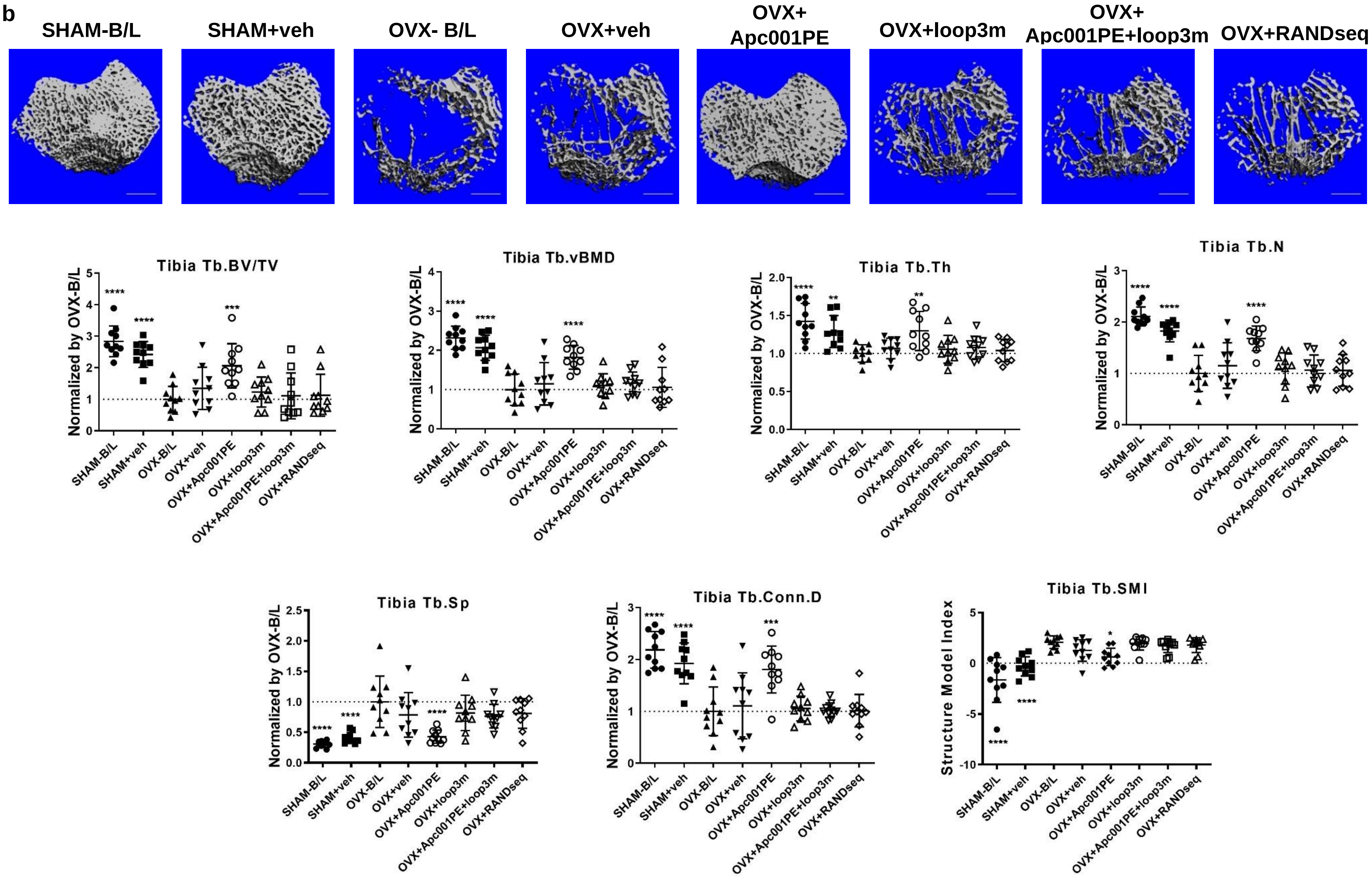
SHAM-B/L
SHAM+veh
OVX- B/L
OVX+veh
OVX+
Apc001PE
OVX+loop3m
OVX+
Apc001PE+loop3m
OVX+RANDseq
Tibia Tb.BV/TV
Tibia Tb.vBMD
Tibia Tb.Th
Tibia Tb.N
Tibia Tb.Sp
Tibia Tb.Conn.D
Tibia Tb.SMI
Normalized by OVX-B/L
Structure Model Index
SHAM-B/L
SHAM+veh
OVX-B/L
OVX+veh
OVX+Apc001PE
OVX+loop3m
OVX+Apc001PE+loop3m
OVX+RANDseq

c

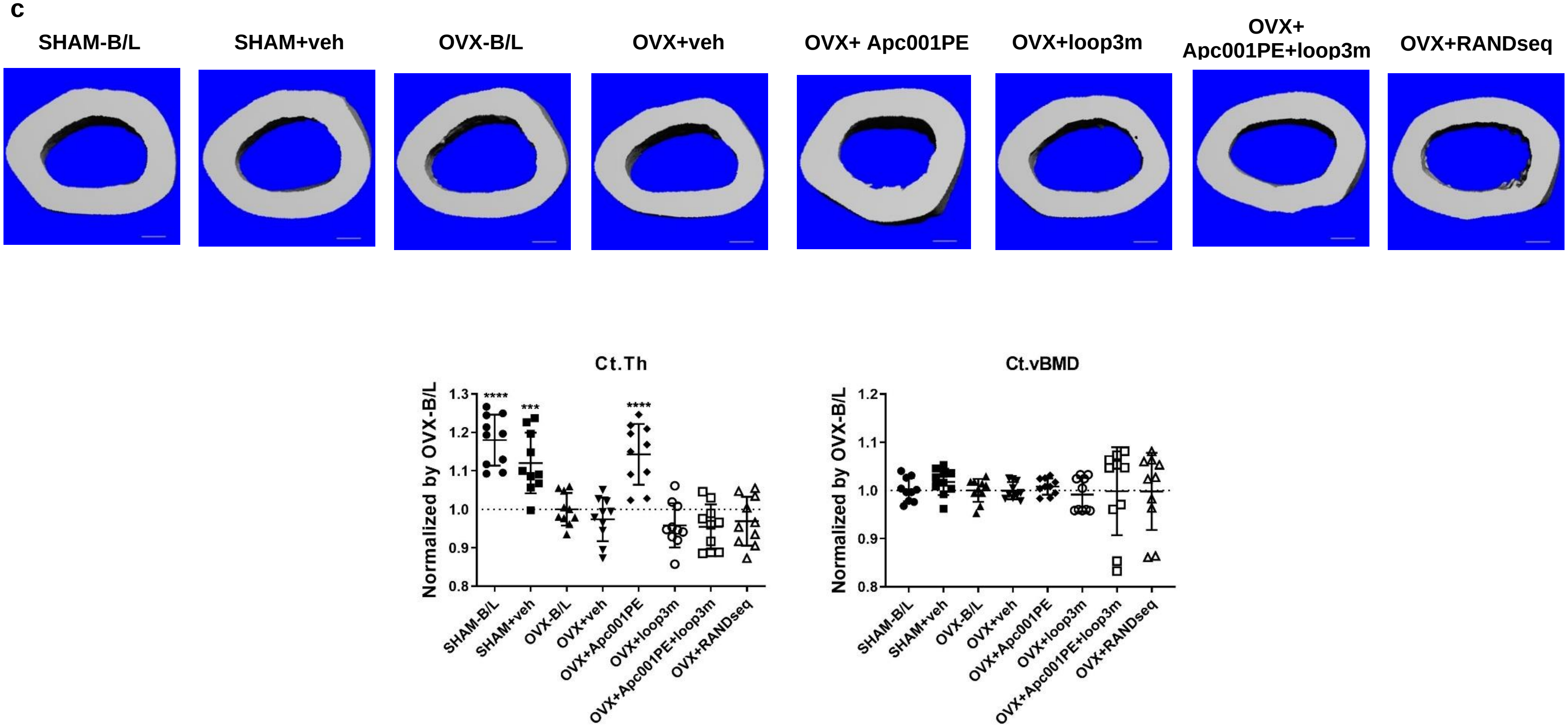
SHAM-B/L
SHAM+veh
OVX-B/L
OVX+veh
OVX+ Apc001PE
OVX+loop3m
OVX+ Apc001PE+loop3m
OVX+RANDseq
Ct.Th
Ct.vBMD
Normalized by OVX-B/L
****
***
****
1.3
1.2
1.1
1.0
0.9
0.8
SHAM-B/L
SHAM+veh
OVX-B/L
OVX+veh
OVX+Apc001PE
OVX+loop3m
OVX+Apc001PE+loop3m
OVX+RANDseq

Supplementary Fig. 19 Determination of whether targeting sclerostin loop3 by the specific *in vivo* pharmacologic tool Apc001PE could exert the bone anabolic potential via neutralizing the circulating sclerostin loop3 in ovariectomy-induced (OVX) osteoporotic rats by micro-CT analysis. a-c Representative images showing three-dimensional trabecular architecture and bar chars of the structural parameters at the distal femur **(a),** the proximal tibia **(b)** and cortical architecture at the femoral mid-shaft **(c)** by micro-CT reconstruction. Scale bars, 1 mm. For **a**, Tb.BV/TV: p<0.0001 (SHAM-B/L, SHAM+veh), p=0.0001 (OVX+Apc001PE); Tb.vBMD: p<0.0001 (SHAM-B/L, SHAM+veh, OVX+Apc001PE); Tb.Th: p=0.0015 (SHAM-B/L), p=0.0279 (SHAM+veh), p=0.0252 (OVX+Apc001PE); Tb.N: p<0.0001 (SHAM-B/L, SHAM+veh), p=0.0019 (OVX+Apc001PE); Tb.Sp: p<0.0001 (SHAM-B/L, SHAM+veh), p=0.0007 (OVX+Apc001PE); Tb.ConnD: p<0.0001 (SHAM-B/L, SHAM+veh), p=0.0049 (OVX+Apc001PE); Tb.SMI: p<0.0001 (SHAM-B/L, SHAM+veh), p=0.0013 (OVX+Apc001PE). For **b**, Tb.BV/TV: p<0.0001 (SHAM-B/L, SHAM+veh), p=0.0007 (OVX+Apc001PE); Tb.vBMD: p<0.0001 (SHAM-B/L, SHAM+veh, OVX+Apc001PE); Tb.Th: p<0.0001 (SHAM-B/L), p=0.0052 (SHAM+veh), p=0.0037 (OVX+Apc001PE); Tb.N: p<0.0001 (SHAM-B/L, SHAM+veh, OVX+Apc001PE); Tb.Sp: p<0.0001 (SHAM-B/L, SHAM+veh, OVX+Apc001PE); Tb.ConnD: p<0.0001 (SHAM-B/L, SHAM+veh), p=0.0002 (OVX+Apc001PE); Tb.SMI: p<0.0001 (SHAM-B/L, SHAM+veh), p=0.0229 (OVX+Apc001PE. For **c**, ct.Th: p<0.0001 (SHAM-B/L, OVX+Apc001PE), p=0.0005 (SHAM+veh). For **a** to **c**, all data were expressed as mean ± standard deviation followed by one-way ANOVA with Tukey's post-hoc test *vs.* OVX-B/L, n = 10 per group. * $p < 0.05$; ** $p < 0.01$; *** $p < 0.005$; **** $p < 0.0001$. **Note:** Tb.BV/TV: trabecular relative bone volume; Tb.vBMD: trabecular volumetric mineral density; Tb.Th: trabecular thickness; Tb.N: trabecular number; Tb.Sp: trabecular spacing; Tb.conn.D: trabecular connect density; Tb.SMI: trabecular structure model index; Ct.vBMD: cortical volumetric mineral density; Ct.Th: cortical thickness. Each representative image was repeated independently for three times with similar results. Source data are provided as a Source Data file.

a

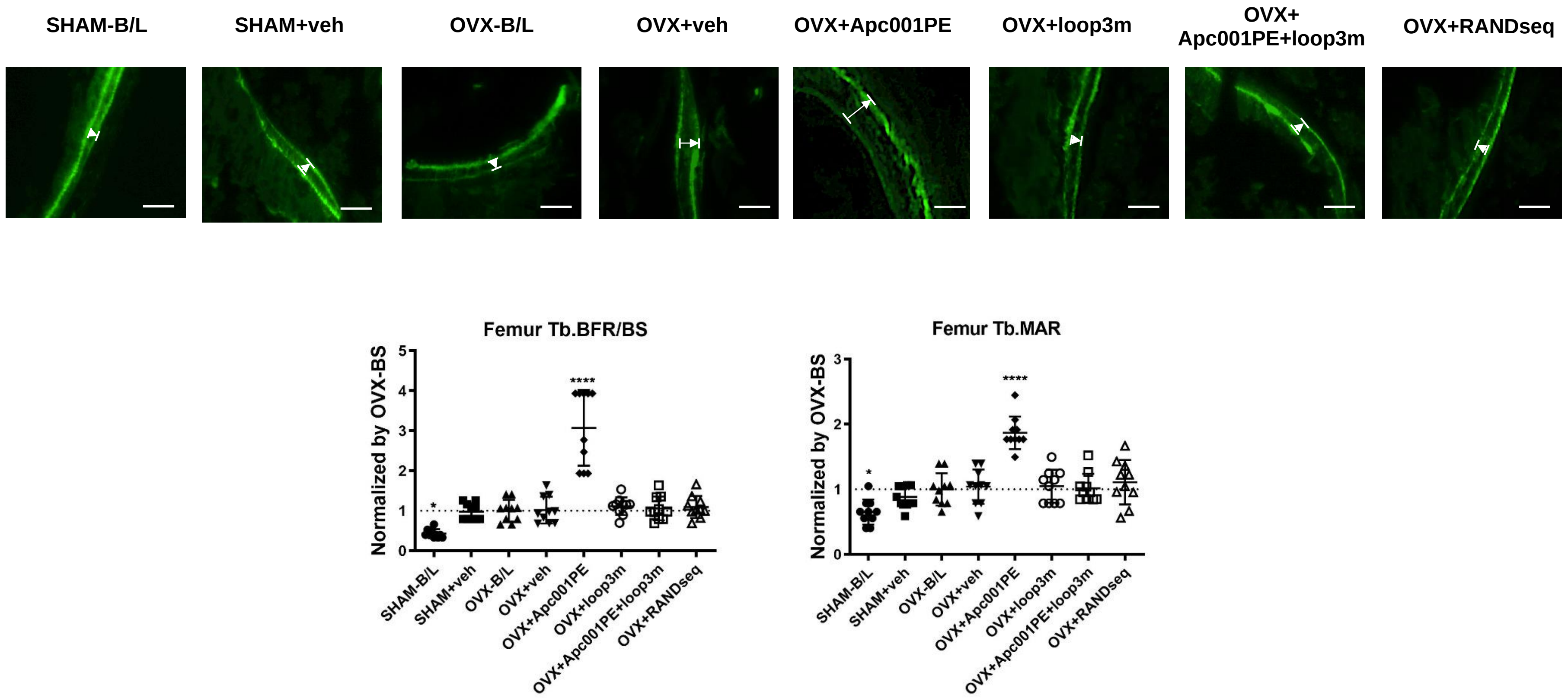

SHAM-B/L
SHAM+veh
OVX-B/L
OVX+veh
OVX+Apc001PE
OVX+loop3m
OVX+ Apc001PE+loop3m
OVX+RANDseq
Femur Tb.BFR/BS
Normalized by OVX-BS
****
*
Femur Tb.MAR

b

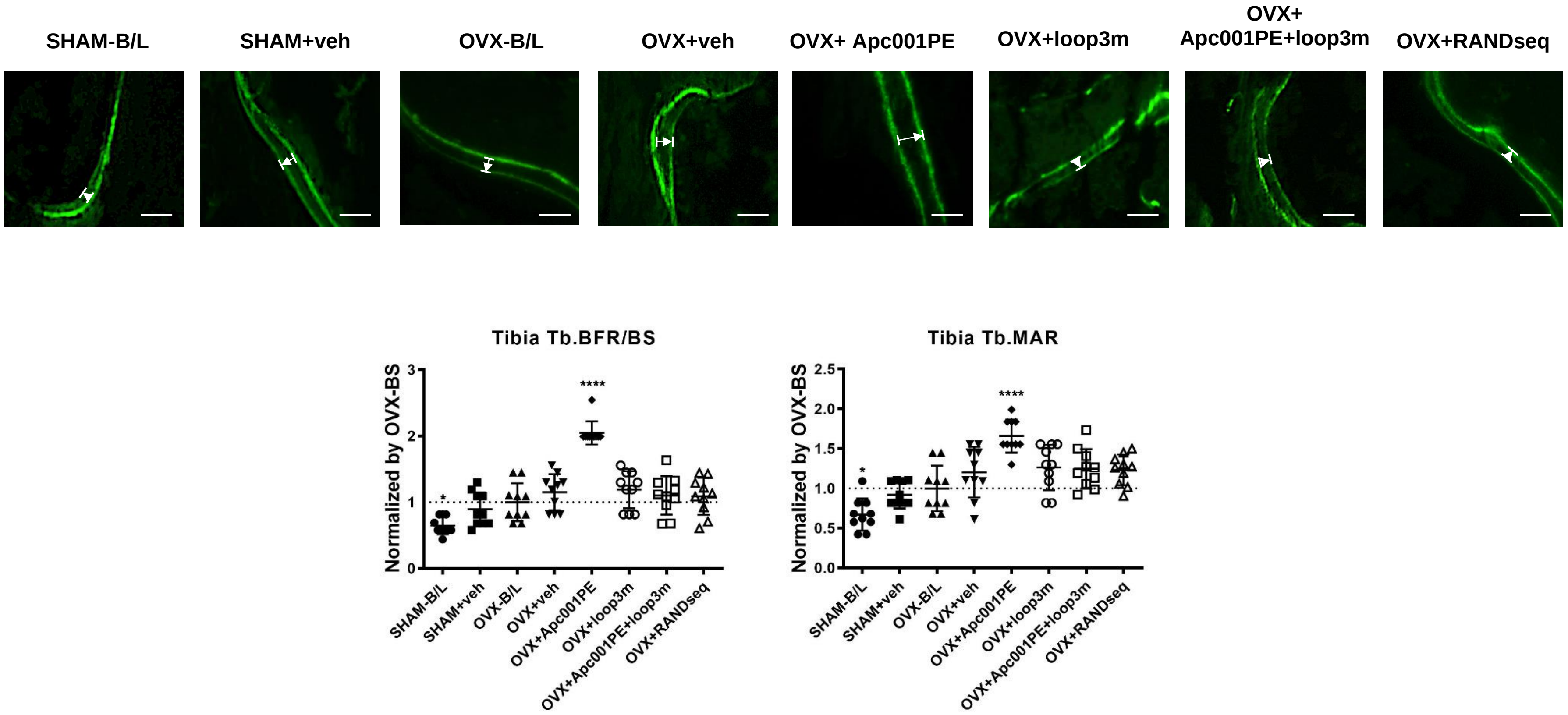
SHAM-B/L
SHAM+veh
OVX-B/L
OVX+veh
OVX+ Apc001PE
OVX+loop3m
OVX+
Apc001PE+loop3m
OVX+RANDseq
Tibia Tb.BFR/BS
Normalized by OVX-BS
Tibia Tb.MAR
Normalized by OVX-BS
SHAM-B/L
SHAM+veh
OVX-B/L
OVX+veh
OVX+Apc001PE
OVX+loop3m
OVX+Apc001PE+loop3m
OVX+RANDseq
*
****
0
1
2
3
0.0
0.5
1.0
1.5
2.0
2.5

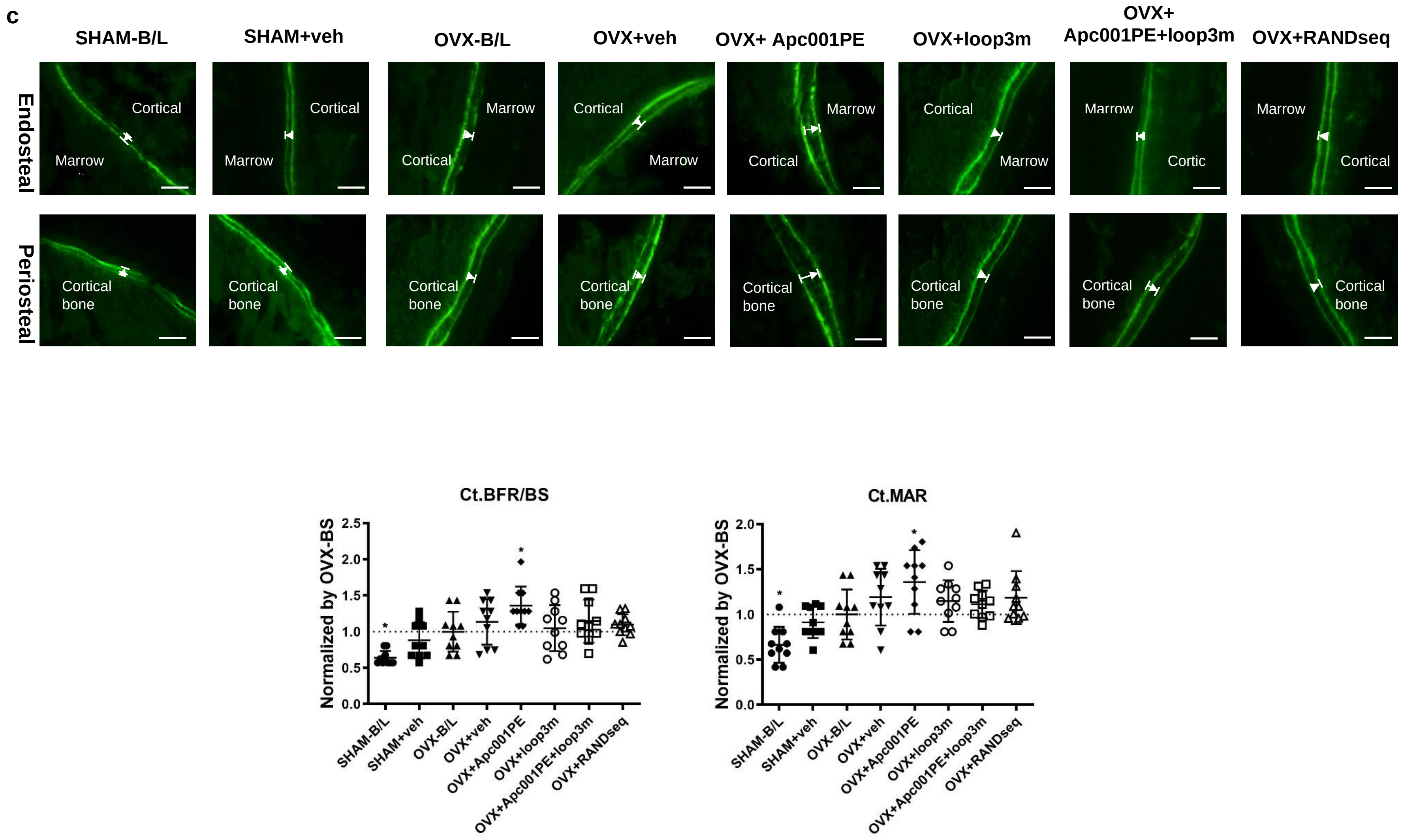
c
SHAM-B/L
SHAM+veh
OVX-B/L
OVX+veh
OVX+ Apc001PE
OVX+loop3m
OVX+
Apc001PE+loop3m
OVX+RANDseq
Endosteal
Periosteal
Cortical
Marrow
Cortical bone
Ct.BFR/BS
Ct.MAR
Normalized by OVX-BS
SHAM-B/L
SHAM+veh
OVX-B/L
OVX+veh
OVX+Apc001PE
OVX+loop3m
OVX+Apc001PE+loop3m
OVX+RANDseq

Supplementary Fig. 20 Determination of whether targeting sclerostin loop3 by the specific *in vivo* pharmacologic tool Apc001PE could exert the bone anabolic potential via neutralizing the circulating sclerostin loop3 in ovariectomy-induced (OVX) osteoporotic rats by histomorphometric analysis. a-c The representative fluorescent micrographs showing bone formation of the trabecular bone sections and bar charts of the dynamic bone histomorphometric parameters at the distal femur **(a)**, the proximal tibia **(b)** and the cortical bone sections at the femoral mid-shaft **(c)** visualized by double calcein labels. Arrows indicated the spaces between the double calcein labeling. Scale bars, 40 μm. For **a**, Tb.BFR/BS: p=0.0160 (SHAM-B/L), p<0.0001 (OVX+Apc001PE); Tb.MAR: p=0.0138 (SHAM-B/L), p<0.0001 (OVX+Apc001PE). For **b**, Tb.BFR/BS: p=0.0153 (SHAM-B/L), p<0.0001 (OVX+Apc001PE); Tb.MAR: p=0.0205 (SHAM-B/L), p<0.0001 (OVX+Apc001PE). For **c**, Tb.BFR/BS: p=0.0146 (SHAM-B/L), p=0.0145 (OVX+Apc001PE); Tb.MAR: p=0.0279 (SHAM-B/L), p=0.0158 (OVX+Apc001PE). **Note:** Tb.BFR/BS: trabecular bone formation rate; Tb.MAR: trabecular mineral apposition rate; Ct.BFR/BS: total (endocortical plus periosteal) cortical bone formation rate; Ct.MAR: total (endocortical plus periosteal) cotical mineral apposition rate. Each representative image was repeated independently for three times with similar results. Source data are provided as a Source Data file.

a

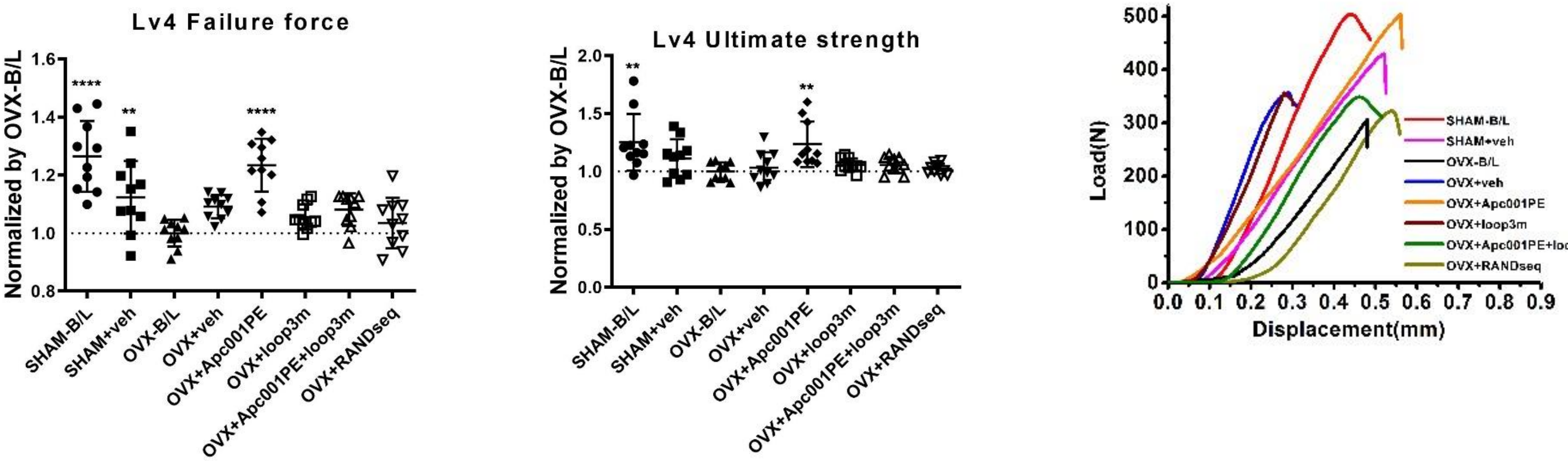
Lv4 Failure force
Normalized by OVX-B/L
Lv4 Ultimate strength
Normalized by OVX-B/L
SHAM-B/L
SHAM+veh
OVX-B/L
OVX+veh
OVX+Apc001PE
OVX+loop3m
OVX+Apc001PE+loop3m
OVX+RANDseq
Load(N)
Displacement(mm)

b

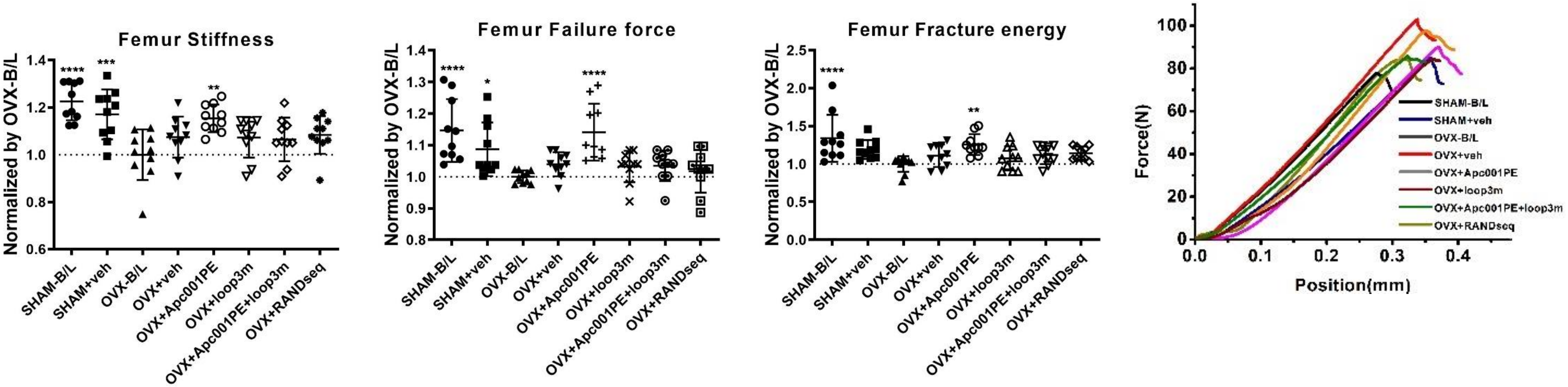
Femur Stiffness
Femur Failure force
Femur Fracture energy
Normalized by OVX-B/L
SHAM-B/L
SHAM+veh
OVX-B/L
OVX+veh
OVX+Apc001PE
OVX+loop3m
OVX+Apc001PE+loop3m
OVX+RANDseq
Force(N)
Position(mm)

c

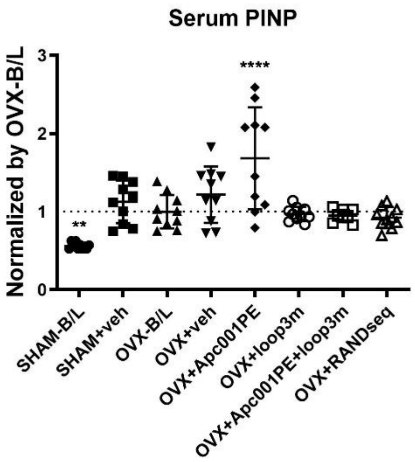

d

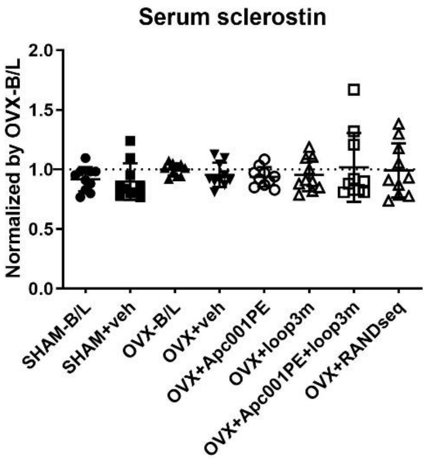

Supplementary Fig. 21 Determination of whether targeting sclerostin loop3 by the specific *in vivo* pharmacologic tool Apc001PE could exert the bone anabolic potential via neutralizing the circulating sclerostin loop3 in ovariectomy-induced (OVX) osteoporotic rats by mechanical test and bone formation marker analysis. a The compression test for the normalized value of failure force (left) and ultimate strength (middle) at the fourth vertebrae. Representative curves showing mechanical properties by compression test at the fourth vertebrae (right). Failure force: p<0.0001 (SHAM-B/L, OVX+Apc001PE), p=0.0082 (SHAM+veh); ultimate strength: p=0.0010 (SHAM-B/L), p=0.0024 (OVX+Apc001PE). **b** The three-point bending test for the normalized value of stiffness, failure force, fracture energy at the femoral mid-shaft, and representative curves showing mechanical properties at the femoral mid-shaft (right). Stiffness: p<0.0001 (SHAM-B/L), p=0.0003 (SHAM+veh), p=0.0014 (OVX+Apc001PE); failure force: p<0.0001 (SHAM-B/L, OVX+Apc001PE), p=0.0369 (SHAM+veh); fracture energy: p<0.0010 (SHAM-B/L), p=0.0043 (OVX+Apc001PE). **c** Analysis for the serum levels of bone formation marker PINP. p=0.0088 (SHAM-B/L), p<0.0001 (OVX+Apc001PE). **d** ELISA for determining the serum levels of sclerostin after treatments. For **a** to **c**, all data were expressed as mean ± standard deviation followed by one-way ANOVA with Tukey's post-hoc test *vs* OVX-B/L, n = 10 per group. * *p < 0.05; ** p < 0.01; *** p < 0.005; **** p < 0.0001.* **Note:** PINP: Terminal Propeptide of Type I Collagen. Source data are provided as a Source Data file.

Supplementary Table 1 The sequences of the aptamer candidates against sclerostin.

Name	Sequence	Specificity to sclerostin loop3	Kd (nM)	IC_{50} (μg/ml)
1	ATTGTTCATGTGTGAAGGGGCCGCGGAGGATTAGGCAAGC	No	-	-
2	GTCAGCTCTTGTGGTCCTCGCAGCGAAAAGGCTCTGGCGG	Yes	-	-
6	TGGAAGGGTGGGGGCGGGGGGTCCTCGCCTCGAACGTACG	Yes	4.2	36.8
9	TGGGGGTAGGGGGACCCTGGCTAGTTAGTCACCGTTTCGA	Yes	3.4	No Inhibition
11	AGGAGACAAATGGGGTAAGGGGCCCTCATTTCAGTACG	No	-	-
15	GAGGGGGCCAACTATGCTTAGTGGGGGGGTTGACCGTATC	Yes	4	No Inhibition
28	GTGGGTTCCAGAAAGGGGGTATCAGTTCGAATAAAGTACG	No	-	-
36	GGGGGTTTCCAGGGGGTCCTTGATGCTAGATTGTATG	Yes	N/A	-
46	GGGTGGATTAAGGGGGCCCCGTCTGTAGGCGATTGGCGAA	Yes	45.6	No Inhibition
51	TGGGGGTAGGGGGTCCTTGGCTACGGTGATTCGGATGTGA	Yes	62.2	No Inhibition
56	CGGGGTGTGGGTTCGTCGTTAGCTTGATTTGGCAGCTGCC	Yes	43.1	19.7
62	CCATGAGTGGGAAAGTTGGGTGGATACTTGGAGGTTGTCC	No	-	-
107	GGGTGGTTTATTGCGGGAGGGCACAGAGAGACGGAGAAGC	No	-	-
132	CCCAGACGAGACACCTCATGCTTTTCCCCGGGGGAGGGGTAT	Yes	42.2	No Inhibition
136	AGAGTTATAAATGGTAGAGGGAGCGGGCTAACCGGCTGTG	Yes	N/A	-
140	TAGGATTGGCGGGCATTGCACTCCTTCAGGAGGTACGGTC	Yes	N/A	-
antibody	--	No	3.8	86.7
