# Supplementary material for: Targeting loop3 of sclerostin preserves its cardiovascular protective action and promotes bone formation
Source: Nat Commun. 2022 Jul 22;13:4241. doi: 10.1038/s41467-022-31997-8 (PMC9307627; doi:10.1038/s41467-022-31997-8)
Supplement: Supplementary file 2 — Reporting Summary [file 41467_2022_31997_MOESM2_ESM.pdf]

nature research

Corresponding author(s): Ge Zhang, Yuanyuan Yu, Aiping Lu, Bao-Ting Zhang

Last updated by author(s): Jul 7, 2022

Reporting Summary

Nature Research wishes to improve the reproducibility of the work that we publish. This form provides structure for consistency and transparency in reporting. For further information on Nature Research policies, see our Editorial Policies and the Editorial Policy Checklist.

Statistics

For all statistical analyses, confirm that the following items are present in the figure legend, table legend, main text, or Methods section.

n/a	Confirmed	
☐	☒	The exact sample size (*n*) for each experimental group/condition, given as a discrete number and unit of measurement
☐	☒	A statement on whether measurements were taken from distinct samples or whether the same sample was measured repeatedly
☐	☒	The statistical test(s) used AND whether they are one- or two-sided<br>*Only common tests should be described solely by name; describe more complex techniques in the Methods section.*
☐	☒	A description of all covariates tested
☐	☒	A description of any assumptions or corrections, such as tests of normality and adjustment for multiple comparisons
☐	☒	A full description of the statistical parameters including central tendency (e.g. means) or other basic estimates (e.g. regression coefficient) AND variation (e.g. standard deviation) or associated estimates of uncertainty (e.g. confidence intervals)
☐	☒	For null hypothesis testing, the test statistic (e.g. *F*, *t*, *r*) with confidence intervals, effect sizes, degrees of freedom and *P* value noted<br>*Give P values as exact values whenever suitable.*
☒	☐	For Bayesian analysis, information on the choice of priors and Markov chain Monte Carlo settings
☒	☐	For hierarchical and complex designs, identification of the appropriate level for tests and full reporting of outcomes
☐	☒	Estimates of effect sizes (e.g. Cohen's *d*, Pearson's *r*), indicating how they were calculated

Our web collection on statistics for biologists contains articles on many of the points above.

Software and code

Policy information about availability of computer code

Data collection	Image J
Data analysis	GraphPad Prism (version 8), Origin 8.5, DAS 3.0 (BioGuider Co., Shanghai, China)

For manuscripts utilizing custom algorithms or software that are central to the research but not yet described in published literature, software must be made available to editors and reviewers. We strongly encourage code deposition in a community repository (e.g. GitHub). See the Nature Research guidelines for submitting code & software for further information.

Data

Policy information about availability of data

All manuscripts must include a data availability statement. This statement should provide the following information, where applicable:

- Accession codes, unique identifiers, or web links for publicly available datasets
- A list of figures that have associated raw data
- A description of any restrictions on data availability

All data generated or analysed during this study are included in this published article (and its supplementary information files). Source data are provided with this paper.

Field-specific reporting

Life sciences study design

All studies must disclose on these points even when the disclosure is negative.

Sample size	The calculation of the sample size in this study is according to the following formula: N=2[(Uα+Uβ) S/δ]2. S in the formula referred to the SD. δ in the formula represented the inter-group difference of the mean values. U0.05 = 1.960, ß = 0.10, U0.1 = 1.282.
Data exclusions	The animals in poor body condition were excluded.
Replication	Attempts of replication for all experiments were successful.
Randomization	The animals were grouped randomly .
Blinding	The animals were grouped blindly to researchers.

Reporting for specific materials, systems and methods

We require information from authors about some types of materials, experimental systems and methods used in many studies. Here, indicate whether each material, system or method listed is relevant to your study. If you are not sure if a list item applies to your research, read the appropriate section before selecting a response.

Materials & experimental systems

n/a	Involved in the study	
☐	☒	Antibodies
☐	☒	Eukaryotic cell lines
☐	☐	Palaeontology and archaeology
☐	☒	Animals and other organisms
☐	☐	Human research participants
☐	☐	Clinical data
☐	☐	Dual use research of concern

Methods

n/a	Involved in the study	
☐	☐	ChIP-seq
☐	☐	Flow cytometry
☐	☐	MRI-based neuroimaging

Antibodies

Antibodies used	Rabbit anti-CD68 antibody (Abcam, ab125212), Rabbit anti-α-SMA antibody (Abcam, ab5694), Rabbit anti-cleaved caspase-3 antibody (Abcam, ab2302), anti-sclerostin antibody (Abcam, ab85799/ab63097), anti-6xHis tag antibody (Abcam, ab245114), Goat anti-rabbit IgG (Abcam, ab205718). Antibodies from ELISA kits including ELISA kits for IL-6 (BMS603-2), TNF-α (BMS607-3), MCP-1 (BMS6005)9 from ThermoFisher, PINP (LS-F23905) from LifeSpan, sclersotin (DSST00) from R&D Systems. Serum levels of immune factors (TNF-α, IL-1β, IL-10, IL-12, IL-18 and IL-4) were determined using a tailored MILLIPLEX MAP rat cytokine kit (Millipore, Billerica, MA, USA).
Validation	Species of primary antibodies were validated using rat and mice tissues.

Eukaryotic cell lines

Policy information about cell lines

Cell line source(s)	MC3T3-E1 subclone 4 (mouse preosteoblast, ATCC CRL-2593), RAW 264.7 (macrophage, ATCC TIB-71), Human VSMC (aorta/smooth muscle, ATCC CRL-19
Authentication	MC3T3-E1 cells were authenticated by analyzing the total RNA with mouse cDNA probe (Wang et al, 1999). RAW 264.7 cells were authenticated by RT-PCR (Ralph et al, 1977). Human VSMC cells were authenticated by spectral karyotyping according to the manufacturer's protocol (Applied Spectral Imaging) (Lazar-Karsten et al., 2016).
Mycoplasma contamination	No mycoplasma infection was found for all cell cultures.
Commonly misidentified lines (See ICLAC register)	N/A

Palaeontology and Archaeology

Specimen provenance	N/A
Specimen deposition	N/A

Dating methods	N/A

☐ Tick this box to confirm that the raw and calibrated dates are available in the paper or in Supplementary Information.

Ethics oversight	N/A

Note that full information on the approval of the study protocol must also be provided in the manuscript.

Animals and other organisms

Policy information about studies involving animals; ARRIVE guidelines recommended for reporting animal research

Laboratory animals	Transgenic C57BL/6 mice expressing human sclerostin (B6. hSOSTki), 3-month, male and female; loop3 deficient human sclerostin (B6. Δloop3-hSOSTki), 3-month, male and female, and wide-type C57BL/6 mice, 3-month, male and female were used to evaluate the bone anabolic potential (n=10 per group). Sprague Dawley rats, 4-month, female, were used evaluate the bone anabolic potential, serum levels immune factors and toxicities (n=10 per group), and to perform the pharmacokinetic analysis (n=6 per group). Apolipoprotein E null (B6. ApoE-/-) mice (C57BL/6J background), 3-month, male; wide-type C57BL/6J, 3-month, male and female; hSOSTki. ApoE-/- mice (B6.hSOSTki mice crossed to B6. ApoE-/- mice), 3-month, male and female; Δloop3-hSOSTki. ApoE-/- mice (B6. Δloop3-hSOSTki mice crossed to B6. ApoE-/- mice), 3-month, male and female, and wide-type C57BL/6 mice, 3-month, male and female were used to evaluate the cardiovascular events (n=9 per group). Wide-type C57BL/6 mice, 3-month, male and female were used to determine the aptamer distribution in organs (n=6 per group).
Wild animals	N/A
Field-collected samples	N/A
Ethics oversight	The procedures of all in vivo studies have gained ethics approval by the Research Ethics Committee of the Hong Kong Baptist University.

Note that full information on the approval of the study protocol must also be provided in the manuscript.

Human research participants

Policy information about studies involving human research participants

Population characteristics	N/A
Recruitment	N/A
Ethics oversight	N/A

Note that full information on the approval of the study protocol must also be provided in the manuscript.

Clinical data

Policy information about clinical studies

All manuscripts should comply with the ICMJEguidelines for publication of clinical research and a completedCONSORT checklist must be included with all submissions.

Clinical trial registration	N/A
Study protocol	N/A
Data collection	N/A
Outcomes	N/A

Dual use research of concern

Policy information about dual use research of concern

Hazards

Could the accidental, deliberate or reckless misuse of agents or technologies generated in the work, or the application of information presented in the manuscript, pose a threat to:

No	Yes	
[x]	[ ]	Public health
[x]	[ ]	National security
[x]	[ ]	Crops and/or livestock
[x]	[ ]	Ecosystems
[x]	[ ]	Any other significant area

Experiments of concern

Does the work involve any of these experiments of concern:

No	Yes	
[x]	[ ]	Demonstrate how to render a vaccine ineffective
[x]	[ ]	Confer resistance to therapeutically useful antibiotics or antiviral agents
[x]	[ ]	Enhance the virulence of a pathogen or render a nonpathogen virulent
[x]	[ ]	Increase transmissibility of a pathogen
[x]	[ ]	Alter the host range of a pathogen
[x]	[ ]	Enable evasion of diagnostic/detection modalities
[x]	[ ]	Enable the weaponization of a biological agent or toxin
[x]	[ ]	Any other potentially harmful combination of experiments and agents

ChIP-seq

Data deposition

- [ ] Confirm that both raw and final processed data have been deposited in a public database such as GEO.
- [ ] Confirm that you have deposited or provided access to graph files (e.g. BED files) for the called peaks.

Data access links<br>*May remain private before publication.*	N/A
Files in database submission	N/A
Genome browser session<br>(e.g. UCSC)	N/A

Methodology

Replicates	N/A
Sequencing depth	N/A
Antibodies	N/A
Peak calling parameters	N/A
Data quality	N/A
Software	N/A

Flow Cytometry

Plots

Confirm that:

- [ ] The axis labels state the marker and fluorochrome used (e.g. CD4-FITC).
- [ ] The axis scales are clearly visible. Include numbers along axes only for bottom left plot of group (a 'group' is an analysis of identical markers).
- [ ] All plots are contour plots with outliers or pseudocolor plots.
- [ ] A numerical value for number of cells or percentage (with statistics) is provided.

Methodology

Sample preparation	N/A
Instrument	N/A
Software	N/A
Cell population abundance	N/A
Gating strategy	N/A

☐ Tick this box to confirm that a figure exemplifying the gating strategy is provided in the Supplementary Information.

Magnetic resonance imaging

Experimental design

Design type	N/A
Design specifications	N/A
Behavioral performance measures	N/A

Acquisition

Imaging type(s)	N/A
Field strength	N/A
Sequence & imaging parameters	N/A
Area of acquisition	N/A

Diffusion MRI ☐ Used ☐ Not used

Preprocessing

Preprocessing software	N/A
Normalization	N/A
Normalization template	N/A
Noise and artifact removal	N/A
Volume censoring	N/A

Statistical modeling & inference

Model type and settings	N/A
Effect(s) tested	N/A

Specify type of analysis: ☐ Whole brain ☐ ROI-based ☐ Both

Statistic type for inference (See Eklund et al. 2016)	N/A
Correction	N/A

Models & analysis

n/a	Involved in the study
☐	☐ Functional and/or effective connectivity
☐	☐ Graph analysis
☐	☐ Multivariate modeling or predictive analysis

Functional and/or effective connectivity	N/A
Graph analysis	N/A
Multivariate modeling and predictive analysis	N/A
